# The Resistance of *Drosophila melanogaster* to Oxidative, Genotoxic, Proteotoxic, Osmotic Stress, Infection, and Starvation Depends on Age According to the Stress Factor

**DOI:** 10.3390/antiox9121239

**Published:** 2020-12-07

**Authors:** Alexei A. Belyi, Alexey A. Alekseev, Alexander Y. Fedintsev, Stepan N. Balybin, Ekaterina N. Proshkina, Mikhail V. Shaposhnikov, Alexey A. Moskalev

**Affiliations:** 1 Laboratory of Geroprotective and Radioprotective Technologies, Institute of Biology, Komi Science Centre, Ural Branch, Russian Academy of Sciences, 28 Kommunisticheskaya st., 167982 Syktyvkar, Russia; alichio13@gmail.com (A.A.B.); alexanderfedintsev@yandex.ru (A.Y.F.); kateplus@mail.ru (E.N.P.); shaposhnikov@ib.komisc.ru (M.V.S.); 2 Department of Biophysics, Faculty of Physics, Lomonosov Moscow State University, 119991 Moscow, Russia; alekseev@physics.msu.ru (A.A.A.); sn.balybin@physics.msu.ru (S.N.B.)

**Keywords:** *Drosophila melanogaster*, aging, stress resistance, biomarkers of aging, modeling

## Abstract

We studied how aging affects the ability of *Drosophila melanogaster* to tolerate various types of stress factors. Data were obtained on the resistance of *D. melanogaster* to oxidative and genotoxic (separately paraquat, Fe^3+^, Cu^2+^, and Zn^2+^ ions), proteotoxic (hyperthermia, Cd^2+^ ions), and osmotic (NaCl) stresses, starvation, and infection with the pathological *Beauveria bassiana* fungus at different ages. In all cases, we observed a strong negative correlation between age and stress tolerance. The largest change in the age-dependent decline in survival occurred under oxidative and osmotic stress. In most experiments, we observed that young *Drosophila* females have higher stress resistance than males. We checked whether it is possible to accurately assess the biological age of *D. melanogaster* based on an assessment of stress tolerance. We have proposed a new approach for assessing a biological age of *D. melanogaster* using a two-parameter survival curve model. For the model, we used an algorithm that evaluated the quality of age prediction for different age and gender groups. The best predictions were obtained for females who were exposed to CdCl_2_ and ZnCl_2_ with an average error of 0.32 days and 0.36 days, respectively. For males, the best results were observed for paraquat and NaCl with an average error of 0.61 and 0.68 days, respectively. The average accuracy for all stresses in our model was 1.73 days.

## 1. Introduction

Aging is a result of the accumulation of damaging effects of metabolic errors and external stressors during an organism’s lifespan. This process is accompanied with the failure of homeostasis systems, which increases the probability of a number of diseases and death. During aging, negative changes in physiological functions and homeostasis occurs at all levels—from molecular to organismal [1]. At the molecular level, aging is associated with a discoordination of signaling pathways, an impairment of antioxidant defense, response to DNA and protein damages, a decrease in the efficiency of repair systems, epigenetic changes, and mitochondrial dysfunction, which results in an increased production of reactive oxygen species (ROS), accretion of cellular damages, and energy deficiency [2,3,4,5,6,7]. An organism accumulates senescent and malfunctioning cells, which negatively affects the functioning of organs and tissues, as well as general health [8,9]. Oxidative, genotoxic, and proteotoxic stresses increase with age and lead to a breakdown of homeostasis systems and a disruption of organism’s adaptive responses [7]. Regardless of a type of stress, many aspects of homeostasis are universal, as it is based on the combined actions of various signaling pathways. Various stressors, directly or indirectly through intermediate stages, lead to an increased oxidative stress and molecular damages in an organism. In turn, aging affects many systems [10].

Despite there being studies showing that resistance to stress and longevity can be experimentally separated, in most cases, higher resistance to various stressors is associated with a long lifespan [11]. This effect is observed in many species, including *Drosophila melanogaster* [12,13,14]. In contrast, accelerated aging is linked to reduced stress resistance [15]. At the same time, the gradual decline in the ability to respond to stress with age may be a general mechanism common for many living organisms [1,16]. Thus, aging-associated changes in the ability to tolerate environmental stresses are related to the aging rate and may be used to predict the organism’s age [17].

A comparison of an actual chronological age and a predicted age allows us to estimate an organism’s biological age. The biological age enables us to assess an aging rate and predict a lifespan better than the chronological age [18]. It can be also used as an indicator of the health status and effects of lifestyle changes on the aging rate. Thus, this knowledge can help to plan a treatment and to prevent a progression of age-dependent diseases [19,20].

Currently, a number of genetic and phenotypic biomarkers, which reflect the organism’s ability to maintain homeostasis and characterize its aging, are discovered in many organisms [21,22,23,24]. The examples of such markers include fecundity dynamics [25,26], physical activity [27], DNA damage markers (phosphorylated histone γ-H2AX, 8-hydroxy-2’-deoxyguanine) [28], the telomere shortening rate and expression levels of proteins associated with telomere dysfunction [28,29], DNA methylation in GpC regions [28,30], changes in expression patterns of non-coding RNAs [31,32,33], levels of heat shock proteins [34], and mitochondrial parameters (degree of oxidative phosphorylation, number of mtDNA copies, degree of heteroplasmy, expression of mitochondrial proteases, and others) [28]. In addition, specific changes are observed in transcriptome profiles. Analysis of the gene expression in fruit flies, nematodes, and some mammalian tissues showed that the expression of mitochondrial genes decreases with age, and the expression of genes involved in the innate immune response and response to oxidative and proteotoxic stress increases [35,36,37,38,39,40]. Expression of stress response genes can be a diagnostic marker for determining a biological age and an effectiveness of used geroprotectors [41].

In this work, we analyzed whether it is possible to estimate the functional state and age of fruit flies by their resistance to stress factors. The model gives an idea of the average biological age of *D. melanogaster* groups. This is a new approach of the *D. melanogaster*’s biological age estimation. We used oxidative and genotoxic stressors, starvation, proteotoxic stressors, osmotic stressors, and fungal infection (by *Beauveria bassiana*) to examine stress tolerance. In this study, we did not analyze age-related changes in tolerance to psychological stresses, such as sleep deprivation and social isolation. This lack is due to weakly pronounced effects of these stresses on survival and lifespan [42,43]. Resistance rates to stress factors, which have the strongest ability to predict the aging rate and lifespan of *D. melanogaster* groups, are determined in the work.

In the work, we used a two-parameter model of survival curves that describes mortality induced by various stressors to analyze our experimental data. For this model, we used an algorithm of the age evaluation, which compares the distribution of model parameters obtained for a) initial reference curves for flies with a known age and b) the curves which correspond to flies of an unknown age, which is being determined. For that, we made a generation of virtual data. That is, we have obtained the generated parameters of the model describing a survival of flies with a specific age, which we consider as unknown. The stability of predictions was assessed by bootstrapping. In addition, we proposed an algorithm for assessing the quality of the age predictions. Thus, obtained parameters of survival curves enabled us to identify the relationships among the chronological age and the rate of stress resistance changes.

## 2. Materials and Methods

### 2.1. Drosophila Rearing

The wild type *D. melanogaster* strain *Canton-S* (#64349, Bloomington *Drosophila* Stock Center, USA) was maintained for more than two years by mass transfer at 25 °C, 60% relative humidity, and 12-h lighting regime on food medium containing 1000 mL water, 7 g agar, 8 g dry yeast, 30 g sugar, 30 g semolina, and 3 mL propionic acid. Experimental animals were kept in a climate chamber Panasonic MIR-554-PE on the same food medium. To reduce condensation, the chamber was running in low humidity mode (relative humidity varied between 50–60%).

To avoid overpopulation in the experimental groups, 10 pairs of males and females per vial were used for 24-h egg-laying. After the imago hatching, we collected flies from the vials using diethyl ether anesthesia and separated them by the sex. For each experiment, the flies were kept in 45 mL vials with 5 mL of food medium.

### 2.2. Analysis of Stress Resistance

We tested stress resistance for 10 different age groups from 5 days to 50 days (the step between groups was 5 days). The non-virgin females and males were analyzed separately. We had 150 flies in the same age and sex group. For each experiment, 150 flies of one cohort were divided into five vials (30 individuals in each) to avoid the negative effects of overcrowding. We studied the following stresses: oxidative and genotoxic stress (20 mM paraquat, 10 and 15 mM FeCl_3_, 10 and 15 mM CuSO_4_, 5 and 10 mM ZnCl_2_), starvation, osmotic stress (400 mM NaCl), proteotoxic stress (hyperthermia with 35 °C, 1 and 5 mM CdCl_2_), and fungal infection by *B. bassiana*. When choosing stressors, we tried to cover a wide range of stressors depending on the damaging mechanism. The intensity of stress conditions was experimentally determined on the basis of the ability to induce flies’ death for 5–8 days. If flies die too quickly, then the accuracy of determining the mortality will worsen. If the death of individuals is too slow, it will be difficult to determine the effect of age on stress resistance.

The flies were kept under a stress condition until death. Every two days, the flies were transferred to a new medium. Twice a day, we counted a number of dead flies. To study flies’ mortality under hyperthermia, animals were kept in standard vials on agar-yeast medium at 35 °C. Hyperthermia treatment was performed using a temperature-controlled chamber. Dead flies were counted at room temperature. Our previous experiments demonstrated that, at 35 °C, flies can survive for 24–72 h (depending on the genotype) [44], which corresponds to our requirements in this study.

Under starvation, flies were placed in vials with a filter paper impregnated with 350 µL of distilled water. For treatment with solutions of paraquat and metal salts, flies were transferred in vials with a filter paper impregnated with 350 µL of 5% sucrose solution with a studied compound. For the infection with *B. bassiana*, flies were placed in a Petri dish with fungus in an active sporulation stage (strain F-145 or VKPM, Russia) and shaken for 60 s.

### 2.3. Analysis of Experimental Data

Sets of raw data were obtained for each stress factor and sex (in total, 26 datasets for different ages), which represent the number of dead flies at different time points of the experiment. The mean and median survival times, the time of a 90% mortality rate, and survival curves were calculated. The Pearson correlation coefficient between age and stress tolerance was estimated. We evaluated the impact of age, sex, and the modality of stress factors on the flies’ survival by using Cox proportional hazards models. Data analysis was performed in Statistica 12.0 (StatSoft), online application for survival analysis—OASIS [45], and R (quote).

We built an empirical model of survival under the influence of stress factors using the experimental results. We selected the function *f*(*t*) of two parameters that can serve to describe the experimental dataset.

$$f\left(t\right)=e^{-{\left(\lambda *t\right)}^{\nu }}$$

where *t* is the time at which we calculate the number of living individuals, *λ* > 0, ν > 0—parameters of the model.

For each variant, experimental fitting of the model to experimental data were promoted [46,47]. All obtained curves and the corresponding experimental points are shown in Online Resource 1. Next, we deployed a bootstrapping procedure in order to clarify the definition of the sustainability of the model parameters during the fitting and identifying areas in which the different variants (ages) are separated well (under the same stress factor and sex, but in different ages). We used the bootstrapping algorithm described in Online Resource 2. Thus, instead of a pair of parameters for each variant of impact (one point on the plane: Parameter 1—Parameter 2), a cloud of 50 points was obtained. The clouds of parameters are shown in Online Resource 3.

### 2.4. Age Estimation Algorithm for the Survival Data of Flies with an Unknown Age

To determine the possibility of the age estimation and the accuracy of this estimation for a variant of exposure with a priori unknown age when a stress was applied, the following algorithm has been implemented. We performed the generation of virtual data based on the distribution of the parameters of an adjacent age. That is, we have obtained the generated parameters of the model describing the survival of flies with a specific age, which we consider as unknown (a_v_). The algorithm is described in Online Resource 2. Furthermore, we made the bootstrapping of data with an unknown age of a stress onset and obtained 50 pairs of values for the parameters (par1, par2) and described this cloud of points by an ellipse. The approach to such a description is given in Online Resource 2. We have obtained the parameters of the intermediate ellipses for each variant and for each combination of two adjacent age variants. For each alternative exposure and in the whole range of ages from 5 to 50 days, we have received a set of 180 ellipses in which each is specified by a quintet of parameters. To each of these ellipses, we associated some intermediate age, which varies linearly in a series of ellipses. For each of the 180 ellipses, we calculated the area of intersection with the ellipse obtained by bootstrapping the variant with unknown age (a_v_). The algorithm is based on the Monte Carlo method and is described in Online Resource 2. Thus, the dependence between the intersection area and age, which correspond to ellipses was obtained. Furthermore, it is necessary to approximate the resulting dependence of the ellipses intersection area and age using the Gaussian function. As an estimate for the age unknown variant (a_estim_), we took the average value of the distribution.

Evaluation of the age prediction accuracy for the data with unknown age when the stress began to apply is based on generation virtual data sets, determining their age (a_estim_), and its comparison with the age for which the data were generated (a_v_).

## 3. Results

### 3.1. Age Dynamics of Stress Resistance

**Figure 1:**
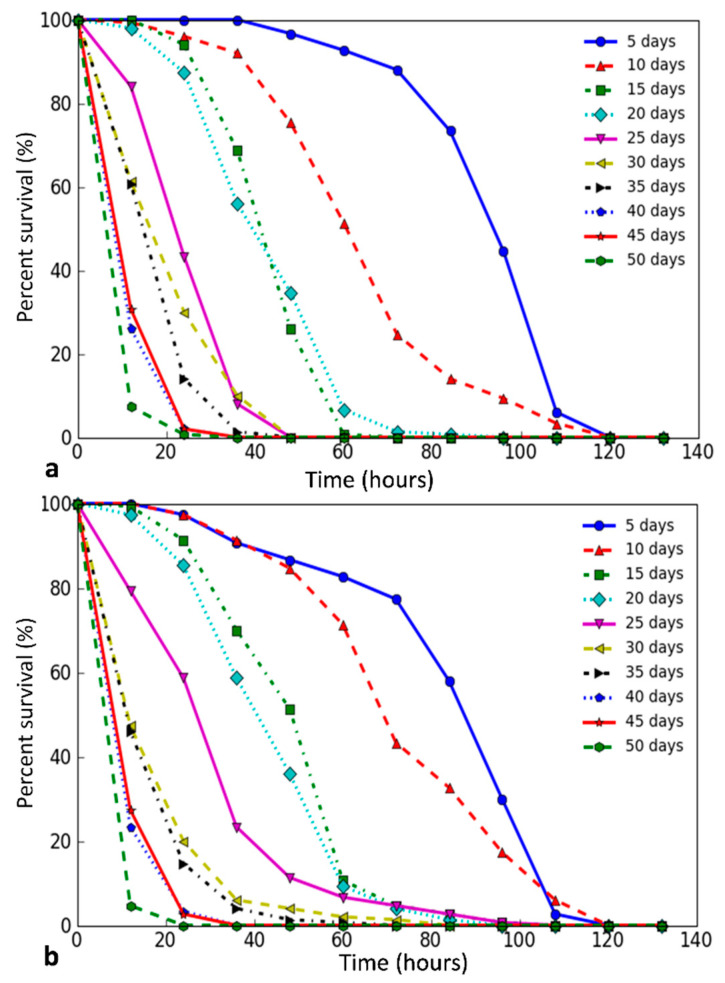
Survival curves of *D. melanogaster* at different ages. The graphs show the change in survival with an increasing age of flies (5, 10, …, 50 days) when exposed to 20 mM paraquat ((**a**)—males, (**b**)—females)).

We have studied the *D. melanogaster* stress resistance at different ages. In flies of both sexes, we observed the gradual and steady decrease in the mean and median survival times, including the time of 90% mortality with age under the influence of all stress factors used in the experiment (Online Resource 4, Table S1), which is well indicated by the variety of survival curves. An example of survival curves for paraquat is shown in Figure 1. All graphs are in Online Resource 4, Figures S1–S4. In all variants of the experiment, a high negative correlation between survival and age in which flies were exposed to stress was revealed (Pearson’s correlation coefficient > 0.7, *p* < 0.05).

**Figure 2:**
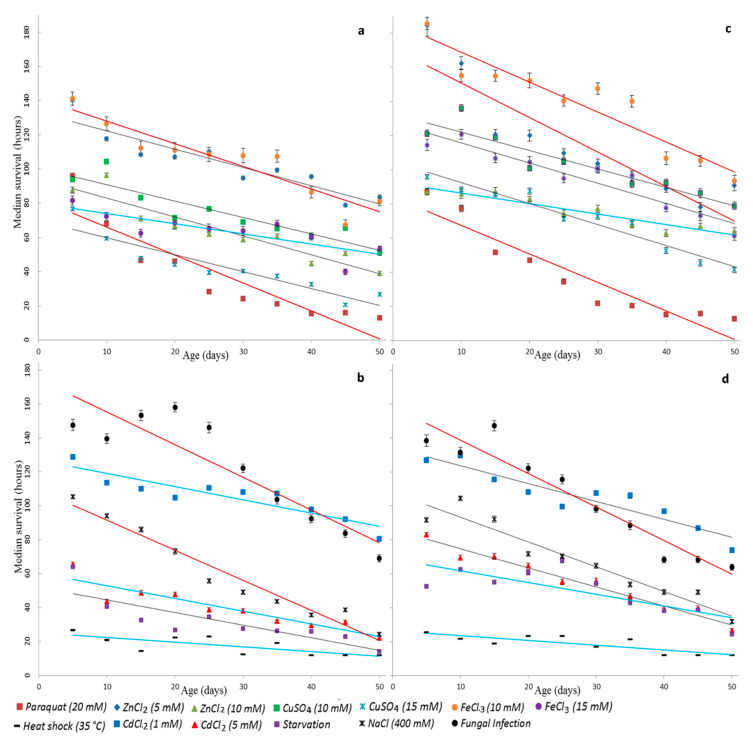
The relationship between the median survival time and the age of flies under the following stress conditions: oxidative and genotoxic stress ((**a**)—male, (**c**)—female), proteotoxic, osmotic stress, starvation, and response to infection ((**b**)—male, (**d**)—females). The lines mark linear regression: red lines show a strong change in survival, gray—middle, blue—slight.

We have plotted scatter diagrams and regression lines to present the relation between the survival time under stress conditions and age (Figure 2). Clouds of variables on scatter diagrams and linear regression have a negative slope in all variants. We have found the general slope of regression lines for all stress factors, and then compared regression coefficients for each variant with a common regression coefficient. During the test, statistically significant heterogeneity of slopes was found using the Student’s *t*-test (the significance level α = 0.05). The most significant age-associated changes in survival of males and females were observed in the case of oxidative and genotoxic stress under the treatment with 10 mM paraquat and iron ions as well as in response to fungal infection by *B. bassiana*. In males, significant changes were also found under osmotic stress, and, in females, significant changes were under the exposure by zinc ions (5 mM). The survival times of males at the variant of proteotoxic stress under the exposure by cadmium ions (1 mM and 5 mM) and hyperthermia as well as the exposure by iron ions (15 mM) were found to have a minimum variance with age. In females, the lowest changes of survival time were revealed under oxidative and genotoxic stress induced by zinc ions (10 mM) and under starvation as well as in males under hyperthermia.

**Figure 3:**
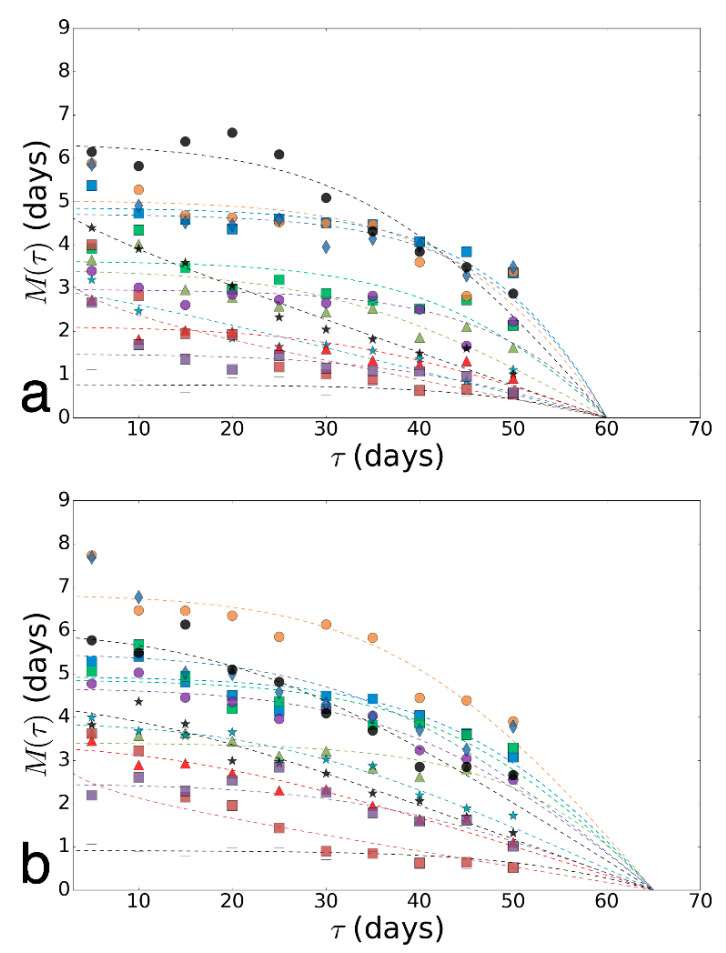
Results of Cox regression calculation for the *D. melanogaster* after exposure to different stressors regardless of the sex of the individual. (**a**)—indicates regression of the risk for flies under the influence of each stressor when the factor is solely the stress, excluding the age of the individual. We have accepted the exposing to paraquat males at the age of 5 days at a basic level. (**b**)—shows the regression of risk for flies under the influence of each of the stressors, with an estimate of the impact of interacting factors (stressor and age of flies). 1.00 hazard ratio, in this case, means no influences of age on the death risk ratio for each stressor.

To assess the influence of the factors and risks associated with them, suggesting only considered factor effects, we used Cox proportional hazards regression models. This method allows us to get the risk of death of an individual and to assess the impact of factors such as sex, age of an individual, and the type of stressor. The analysis results are shown in Figure 3. To estimate the impact of sex and each stress separately on the risk of death, we accepted the survival of five day-aged males under the treatment with paraquat as a basic level. The Cox analysis showed that the female sex is a factor that halves mortality (Hazard ratio = 0.5362, 95% confidence interval = 0.5249–0.5477). The analysis of the effect of each stress factors alone showed that the most powerful impacts are cadmium ions (5 mM), starvation, and hyperthermia, and the least dangerous—infection by *B. bassiana* as well as the impact of ferric (10 mM) and zinc ions (5 mM) (Figure 3).

Interaction of factors “stress” and “age” was further analyzed. The analysis showed that, for both sexes, age is an important risk factor that worsens the prognosis of survival time in all studied stress conditions (Figure 3). The most significant decrease in stress resistance with age occurred under the oxidative and genotoxic stress induced by paraquat (20 mM) at a lesser extent under the osmotic stress. Additionally, the age factor greatly increased the risk of death caused by the fungal infection and treatment with copper ions (15 mM), and a little less under starvation and treatment with cadmium ions (5 mM).

### 3.2. Prediction of the Biological Age

To assess the biological age and determine the accuracy of our predictions, we generated virtual data for survival curves, simulating data for intermediate ages (a_v_) based on the distribution parameters of adjacent ages. Virtual data for the variant of infection by a fungus *B. bassiana* illustrated in Online Resource 2 Figure S2. In our work, we used 100 random sets of virtual data to improve the reliability of evaluation. Then, we calculated the intersection area of ellipses and received average values of the dependence of ellipses’ intersection area(s), built on a bootstrapping cloud of virtual data, and the estimated age (a_estim_). The illustration of the determination of the intersection area of estimated ellipses and ellipse constructed with the virtual data for the case of paraquat exposure on females are shown in Online Resource 2, Figure S5.

We assumed that the maximum value of the intersection area can be obtained for values of the estimated age (a_estim_), which are close to the value of the corresponding intermediate age for a given set of virtual data a_v_, and their difference can be taken as a measure of the accuracy of predictions for a given age range. For the obtained distribution of intersection area from age, we find the first two moments of the distribution (mathematical expectation and variance). The expectation in this case is the sense of estimating age for a given set of virtual data. It allowed us to estimate the age of an unknown variant by the displacement of the mass center of the average intersection area of ellipses from the intermediate age of virtual data a_v_ (Table 1). We determine the deviation of virtual age estimation, which means how well the estimated age a_estim_ and virtual intermediate age coincide. Thus, we evaluated the accuracy rate of the age prediction. This indicator is useful for estimating the age prediction error. Therefore, the value of this index is equal to the prediction error in days. Using this indicator, the different stress factors in different age ranges can be compared to each other with high accuracy by the grade of the prediction quality.

Having obtained the prediction error for different virtual ages (a_v_), different stress factors, and sexes, it can be concluded that resistance to the stressor can give the best prediction in different ranges of ages. We compared the different variants for different ages using the error in days and percentages (Table 1).

1. 5–15 days. The best results were in the range of small ages obtained for oxidative and genotoxic stress in females when exposed by paraquat (Table 1) with an accuracy of age predicting—0.008 days, 0.02%, 0.037 days, 0.07%, and 0.38 days, 0.77% (referred to in parenthesis) and proteotoxic stress when treated with 1 mM CdCl_2_ (0.153 days, 0.31%) and 5 mM CdCl_2_ (0.442 days, 0.88%) (Table 1). For males, the best quality of the predictions obtained under oxidative and genotoxic stress induced by paraquat (0.57 days, 1.15%), 15 mM CuSO_4_ (0.296 days, 0.59%), 10 mM ZnCl_2_ (0.382 days, 0.76%), and osmotic stress (0.405 days, 0.81%).

2. 20–35 days. For females, the best results were obtained in variants with oxidative and genotoxic stress when exposed by paraquat (0.414 days, 0.83%), osmotic stress (0.11 days, 0.23%), response to infection (0.277 days, 0.55%), and under the treatment with cadmium ion at the concentration of 5 mM (0.209 days, 0.42%). For males as well as for females in this range, good results are obtained for the oxidative and genotoxic treatment (paraquat—0.33 days, 0.66%) as well as under osmotic stress (0.358 days, 0.72%).

3. 40–50 days. Consistently high prediction quality for females can be seen under oxidative and genotoxic stress induced by 10 mM FeCl_3_ (0.056 days 0.11%), 15 mM FeCl_3_ (0.095 days, 0.19%), and 15 mM CuSO_4_ (0.285 days, 0.57%). In the variants of the experiment with males, the response to fungal infection should be noted (0.57 days, 1.13%).

The relatively poor quality of the predictions in the whole range of ages obtained by oxidative and genotoxic stress when exposing females by zinc ions at the concentration of 10 mM (1.76 days, 3.5%), 10 mM copper in both sexes (females—1.421 days, 2.84% males—1.116 days, 2.23%), and under proteotoxic stress for hyperthermia (female—5.621 days, 11.24% males—7.185 days, 14.37%). In the variants of the experiment with males, poor quality was observed when treated with proteotoxic cadmium ions at the concentration of 5 mM (1.932 days, 3.86%) and under starvation (1.948 days, 3.9%).

## 4. Discussion

Pathways that regulate the lifespan, aging, and organism’s ability to resist internal or external stressors have similar mechanisms [48,49,50]. The complex action of different signaling pathways leads to maintenance of homeostasis under stress conditions. At the same time, aging as a complex process simultaneously affects many molecular processes, systems, and organs of an organism, leading to deterioration of their functioning. All nine stress factors in our study showed a strong negative correlation between age and stress tolerance. Differential changes in the expression of a wide range of genes play an important role in this reduction in stress resistance. These genes are involved in detoxification of free radicals, xenobiotics, and toxic metals, a response to DNA damage and unfolded proteins, mitochondrial function, regulation of lipid metabolism, and immune and inflammatory responses [35,36,37,38,39,40,51,52,53,54,55]. Aging is associated with a decrease in the efficiency of repair systems and antioxidant defense [2,3,54,55]. This leads to a damage accumulation with age and a tension in the work of compensatory mechanisms. In particular, the accumulation of DNA damages causes cellular aging and metabolic changes that contribute to an impaired tissue function, decreased stress tolerance, and an increased risk of age-related pathological processes [55,56]. Mitochondrial dysfunction also makes a significant contribution to the aging process and age-dependent depletion of stress resistance. On the one hand, it is accompanied by a decrease in the production of ATP and bioenergetic insufficiency of a cell, which causes inhibition of cellular functions, including the work of defense systems. On the other hand, it is aggravated by an increased production of ROS as well as oxidative damage to mitochondrial and cellular macromolecules [57,58,59]. It should be noted that changes in stress response reactions are characterized not so much by their decrease as by general deregulation [6,7,8,59]. As a result, there is a general aging-related deterioration in maintaining physiological functions and homeostasis in an organism [48,60].

In our study, at the early ages, *Drosophila* females have a higher resistance to stress than males. A similar effect of sex was observed for female cells in humans and rodents, which was exposed by oxidative stress [7,61]. Differences are also observed at the molecular level. For example, females of various animals are characterized by a higher antioxidant, detoxifying and proteolytic capacity, and inducibility of defense mechanisms compared to males. As a result, sex differences are observed in the effectiveness of eliminating damaging effects (including the accumulation of xenobiotics in an organism) and the level of damaged macromolecules [7,62,63,64,65]. In humans, the distinction in responses to stress associated with sex observed in all age groups and mediated by the action of hormones and genes in sex chromosomes [65,66,67,68]. In addition, sex differences may be partly caused by asymmetries in mitochondrial inheritance [7].

Despite the fact that dynamics of stress resistance reduction with age was clearly traced in all exposed variants in our study, we observed significant differences associated with the modality of a stress factor. These differences in the dynamics can be explained by the participation of various mechanisms in the stress response and the fact that aging exerts on them an unequal effect [36]. Our research showed that the strongest changes in survival with age for males and females under oxidative and genotoxic stress were observed for paraquat, ferric, and zinc ions in low concentrations and high concentrations of copper ions. Various stressors, directly or indirectly through intermediate stages, lead to increased oxidative stress and molecular damage in an organism. ROS, which provoke oxidative stress, pose a danger to DNA, proteins, and lipids. In particular, ROS modify DNA bases, leading to genome instability and mutation [69]. On the other hand, the observed dynamics is possibly associated with a decrease in the expression of mitochondrial genes with age, and this can lead to an increase in oxidative, genotoxic, and proteotoxic stress in a cell [4,5].

Our results demonstrate that the resistance to the treatment with zinc and ferric ions at the high concentrations varies slightly with age, remaining low throughout the *Drosophila* life. The smallest changes of survival in our study were observed under proteotoxic stress induced by cadmium ions and hyperthermia. Although the proteostasis efficiency decreases with age [6,70], this effect can likely be explained by a high toxicity of stressors or an excessive dose.

Our study of the dynamics of survival changes with age showed a strong decrease in the resistance to fungal infection by *B. bassiana* both for *Drosophila* males and females. It was shown that the immune system deteriorates with age in humans and experimental animals [71,72,73,74]. There is also a destruction of protective barriers against pathogens such as intestinal and tracheoles epithelium. Such abnormality in the immune system likely leads to an increase in the pathogenic load with age that, in turn, enhances the activity of nuclear factor kappa-light-chain-enhancer of activated B cells (NF-κB) and other genes associated with innate immunity [75]. For example, a significant increase in mRNA levels of antimicrobial peptides (AMPs) was shown. It is possible to predict the duration of an individual life by the level of expression of certain AMP genes in young flies [35,76].

Another stress at which the survival rate changed significantly with age is starvation. Likely, the reduction of stress resistance with age that we observed under dehydration and starvation was associated with a change in the energy reserve volume, the energy consumption rate during starvation, and the minimum energy level required for the organism’s survival [77,78]. However, it was found that the content of lipids is increased with age in *Drosophila* females that correlates with increased resistance to starvation [79]. However, the overall ability of an organism to synthesize proteins, maintain energy resources during aging, and the ability of an organism to resist starvation may decrease [80]. According to published data, it is known that there is a relationship between the resistance to dehydration, starvation, oxidative stress, and immune response [78,80,81]. In a recent study, it was shown that the resistance to dehydration and oxidative stress is maximum in the early days of adult life and further dramatically reduced, but resistance to infection and starvation show no significant dynamics in young animals [82]. Thus, even if the stress response has common elements, specificity still occurs because the types of macromolecular damage depend on the type of stress.

We observed a high dynamic of changes in resistance to osmotic stress. Osmotic or salt stress occurs because of abrupt changes in the water balance of an organism. A rapid decline in the resistance to osmotic stress during aging can be attributed to a general decline of the body ability to synthesize metabolites including osmolytes as well as a violation of the expression of stress response genes, such as immune defense genes or heat shock proteins’ genes [83,84,85]. During aging, a protective function of the lipid layer of the cell membrane and cuticle are disrupted as well as a consequence of the decreased activity of antioxidant systems during aging. The supply of water in in an organism also decreases, leading to a more rapid onset of a water deficit in tissues [86,87].

Thus, our study allowed us to estimate changes of stress resistance with age to a wide range of factors. Although certain processes during aging can be specific to insects and flies, their investigation by using *D. melanogaster* provides an understanding of aging mechanisms common to all living organisms. Changes in stress resistance can be used as a biomarker of aging, as it fulfills the following criteria, such as occurring during normal aging, experimental reduction of stress resistance accelerating aging, and an experimental increase of stress resistance slowing the aging process and, consequently, prolonging a healthy life [48,88,89,90].

Biological age in its essence reflects the functional state of an individual, regardless of the chronological age [91]. Chronological age conveys a rough assessment of the state of an organism, whereas biological aging is a consequence of the interaction of genetic, ecological, and behavioral factors, and diseases [92]. Thus, the sub-population of individuals of *Drosophila* with one chronological age will be heterogeneous in the physiological state of individuals with different biological ages. However, it is expected that the influence of a strong factor, such as aging or pharmacological agents, can shift the overall functional state of individuals in a group and, therefore, biological age, even if there is heterogeneity within that group. As our results showed, during a growth in age, the ability to resist the action of stress factors decreases and this allows us to judge the biological age of a particular group of individuals. If any factor that reduces or improves the physiological state of the organism acts on the fruit fly group, it is expected that, in all individuals in this group, the biological age will be correspondingly high or less than the chronological age. Our predictive model allows us to determine the biological age by the mortality rate at studied stressful conditions.

In this work, we proposed an approach for modeling the empirical survival curves as well as a theoretical method for predicting a priori unknown age of flies’ group, according to the results of stress experiments. Based on the developed age determination approach for each data set, we provided a method of quality evaluation of predictions in the range of ages for experiments with the selected stressors and sexes. This method is based on obtaining the “virtual” data for survival curves, which simulate data for intermediate ages. In particular, using data for the ages of 5 and 10 days, it is possible to get a “virtual” data comparable with the age of 7.5 days. Furthermore, for these data, we performed the prediction of age. Then the stress began, and, via dispersion and displacement of the age estimation obtained from this prediction, we have introduced the quality index prediction of age in the vicinity of this age (example—7.5 days) at the specified stress and animals.

As can be seen based on the results, the lowest predicted error of age was received for oxidative and genotoxic stressors, in particular the impact of paraquat, ferric ions in both doses and copper ions in high dose. On average, we have obtained a higher age prediction accuracy for females compared to males. The analysis showed a good prediction accuracy for females under proteotoxic stress induced by cadmium ions, despite the fact that, in this case, there is a low-stress resistance dynamic with aging. Likely, this is due to the smoothness of *Drosophila* stress resistance changes under the treatment with cadmium ions. The best quality predictions for females were under the treatment with CdCl_2_ and ZnCl_2_. For males, the best results were obtained for paraquat and NaCl. In general, none of the stressors were noted with consistently high prediction accuracy for the whole range of ages. Such differences can be explained by different rates of death of males and females under different conditions. Therefore, it is convenient to use certain stressors or their groups on the age interval of interest and on one of sexes. For example, in the young flies, more stable quality was observed when exposed by paraquat, zinc, and cadmium ions. In middle-aged flies, good results were obtained by the action of paraquat, NaCl, and fungal infection. Mainly in the older age groups, good predictions quality was received in variants with osmotic stress, infection, and starvation. The unevenness prediction accuracy can be attributed to different rates of stress resistance changes with age in different periods of life. An improvement of the prediction quality is possible by increasing the frequency of stress resistance measurement points.

To reliably detect changes in an organism with age, our method of determining the functional state of an organism can be used in conjunction with a wide range of biomarkers of aging. Known aging biomarkers include epigenetic changes [93,94], changes of transcriptome [95,96], metabolome [97], the shortening of telomere length [98,99], or changes in signaling pathways during the aging process and in age-related diseases [100]. In aging flies, different molecular changes that may reflect disturbances in the state and functioning of an organism were discovered, in particular protein modifications and aggregation carbonylation [101,102,103,104] as well as lipid peroxidation [105]. The study of the intestinal epithelium status allowed tracking age-related changes of the innate immune and inflammation signaling pathways or the dysregulation of stem cells [106,107,108]. The values of age predicting quality indicators for virtual data allow us to estimate the accuracy of age predictions, which could serve as a basis for selecting the best experimental conditions, i.e., conditions under which the accuracy and uniqueness of biological age predictions maximize in an age range of interest. The created prognostic model enables us, along with other methods of prediction of biological age, to determine factors that accelerate or slow down aging. This method is characterized by a high prediction accuracy, the relative simplicity of use, and low cost. This approach of the assessment of the biological age prediction accuracy will help study the geroprotector effect on populations with different ages, as it allows us to choose specific stress and sex for each of these populations. This permit to give the most accurate assessment of changes in biological age.

It would be important to test the advantages of a described mathematical model on a relevant biological system different from *Drosophila*. For example, taking into account that replicative lifespan of human fibroblasts in culture correlates with donor age [109], we may expect that cell stress resistance estimated by survival will also predict the biological age of humans. However, further studies are required to test this suggestion. It is worth noting that recent research has opened new prospects for the use of artificial neural networks and deep learning in the definition of human biological age [110]. For instance, one of the methods allows inexpensively and quickly determining an age based on different metabolites derived from a sample of peripheral blood, such as albumin [111], glucose [112], and many others [113,114].

## 5. Conclusions

Thus, in this study, we investigated age-related changes in the resistance of *D. melanogaster* to various types of stress factors, including oxidative and genotoxic (separately paraquat, Fe^3+^, Cu^2+^, and Zn^2+^ ions), proteotoxic (hyperthermia, Cd^2+^ ions), and osmotic (NaCl) stresses, starvation, and infection with the pathological *B. bassiana* fungus. In all variants, we observed a high negative correlation between age and stress tolerance. *Drosophila* females have a higher resistance to stress than males (Hazard ratio = 0.5362). The largest age-dependent changes of survival were observed in *Drosophila* under oxidative and genotoxic stress as well as under osmotic stress. The most significant changes in stress resistance were observed under proteotoxic stress and treatment with ferric and zinc ions in high concentrations. The algorithm for assessment of biological age by using a two-parameter model of survival curve has been created. The algorithm is based on a comparison of the distribution of the model parameters obtained for “reference” curves of survival and the survival curve for flies with unknown age of onset of stress. We proposed an algorithm for assessing the quality of biological age predictions. This allows us to obtain prediction accuracy rates for various stresses and sexes. The best quality predictions for females were under exposure by CdCl_2_ and ZnCl_2_ with a mean error for the period—0.32 days and 0.36 days, respectively. For males, the best results were obtained for paraquat and NaCl with a mean error for the period—0.61 days and 0.68 days. On average, for all the stresses, our model determines the biological age with accuracy—1.73 days, 3.47%. Thus, we showed that resistance to stress can serve as an indicator of the lifespan in *Drosophila*. Since there is the evolutionary heredity of stress-resistance mechanisms, we expect that our method can be used in studying the survival of primary cultures of human cells, such as fibroblasts, to predict the biological age of humans.

## Figures and Tables

**Table 1:** The table with the values of the deviation of virtual age estimation for all variants of the experiment shows the age prediction error.

Stressor	Sex	Intermediate Ages (days) ^1^								
		7.5	12.5	17.5	22.5	27.5	32.5	37.5	42.5	47.5
Paraquat	male	0.021	1.458	0.242	0.574	0.41	0.008	1.785	0.554	0.426
Paraquat	female	0.026	0.689	0.436	0.17	0.372	0.701	0.662	0.914	1.909
Starvation	male	1.044	0.894	5.964	2.321	3.24	0.522	1.423	0.273	1.856
Starvation	female	0.149	1.627	0.971	0.835	10.282	0.546	1.421	0.046	0.377
Heat shock	male	11.005	1.842	3.169	2.293	12.721	17.619	11.005	1.842	3.169
Heat shock	female	0.714	10.676	5.475	0.714	10.676	5.475	0.714	10.676	5.475
Fungal infection	male	5.567	0.364	0.976	3.381	0.596	0.101	0.251	0.346	0.169
Fungal infection	female	1.253	0.472	4.116	0.026	0.413	0.392	0.356	0.71	1.476
NaCl (400 mM)	male	0.777	0.156	0.284	0.255	0.29	0.529	3.531	0.274	0.06
NaCl (400 mM)	female	1.477	0.999	0.569	0.127	0.03	0.193	1.634	0.389	0.085
ZnCl_2_ (5 mM)	male	0.166	5.121	2.37	2.181	0.856	0.927	1.232	0.112	0.764
ZnCl_2_ (5 mM)	female	0.008	0.196	1.088	0.445	0.17	0.2	0.25	0.579	0.315
ZnCl_2_ (10 mM)	male	0.401	0.465	0.28	0.312	2.067	1.102	4.551	1.875	0.075
ZnCl_2_ (10 mM)	female	3.388	0.378	0.745	0.445	0.188	0.207	3.247	1.969	5.283
CuSO_4_ (10 mM)	male	0.77	0.684	1.575	0.206	2.014	0.509	3.152	0.849	0.286
CuSO_4_ (10 mM)	female	1.651	2.315	1.201	1.078	3.545	0.384	0.385	1.568	0.667
CuSO_4_ (15 mM)	male	0.405	0.226	0.257	2.355	0.847	1.77	0.096	0.611	2.619
CuSO_4_ (15 mM)	female	0.256	2.042	2.519	0.365	1.039	0.608	0.21	0.252	0.393
CdCl_2_ (1 mM)	male	0.248	2.211	9.968	4.063	2.996	5.207	0.478	0.541	0.623
CdCl_2_ (1 mM)	female	0.067	0.144	0.25	10.195	2.957	1.883	2.348	0.297	0.404
CdCl_2_ (5 mM)	male	7.518	0.085	2.084	4.069	0.419	0.628	2.112	0.166	0.308
CdCl_2_ (5 mM)	female	0.131	1.119	0.076	0.507	0.079	0.042	0.036	0.556	0.314
FeCl_3_ (10 mM)	male	0.184	0.097	3.583	0.114	1.98	0.168	0.069	0.186	0.806
FeCl_3_ (10 mM)	female	0.385	1.201	1.514	2.423	1.443	3.772	0.001	0.144	0.025
FeCl_3_ (15 mM)	male	0.203	11.538	6.478	0.295	3.773	7.816	0.507	0.184	2.977
FeCl_3_ (15 mM)	female	0.691	0.037	0.867	5.516	1.732	2.774	0.28	0.001	0.006

^1^ The values in the cells—deviation of virtual age estimation (days). The intermediate ages correspond to the values a_v_.

## Supplementary Materials

The following are available online at https://www.mdpi.com/2076-3921/9/12/1239/s1. Online Resource 1: Contains the figures that show the fitting of the model to experimental data. Red line shows the original reference points, the red bar line connects mean values, the blue dotted line—the model curve, Online Resource 2: Description of the approach of empirical modeling of survival curves as well as the theoretical method of predicting a priori an unknown age group of individuals, according to the results of stress experiments. Online Resource 3: Shows the results of the original data bootstrapping. Online Resource 4: The data on *Drosophila* melanogaster stress resistance at different ages.
