# Supplementary material for: The Resistance of Drosophila melanogaster to Oxidative, Genotoxic, Proteotoxic, Osmotic Stress, Infection, and Starvation Depends on Age According to the Stress Factor
Source: Antioxidants (Basel). 2020 Dec 7;9(12):1239. doi: 10.3390/antiox9121239 (PMC7762242; doi:10.3390/antiox9121239)
Supplement: Supplementary file 1 [file antioxidants-09-01239-s001.zip › Online Resource 1.pdf]

The resistance of *Drosophila melanogaster* to oxidative, genotoxic, proteotoxic, osmotic stress, infection and starvation depends on age according to the stress factor

Alexei A. Belyi, Alexey A. Alekseev, Alexander Y. Fedintsev, Stepan N. Balybin, Ekaterina N. Proshkina, Mikhail V. Shaposhnikov and Alexey A. Moskalev

Online Resource 1 contains the figures that show the fitting of the model to experimental data. Red line shows the original reference points, the red bar line connects mean values, the blue dotted line - the model curve.

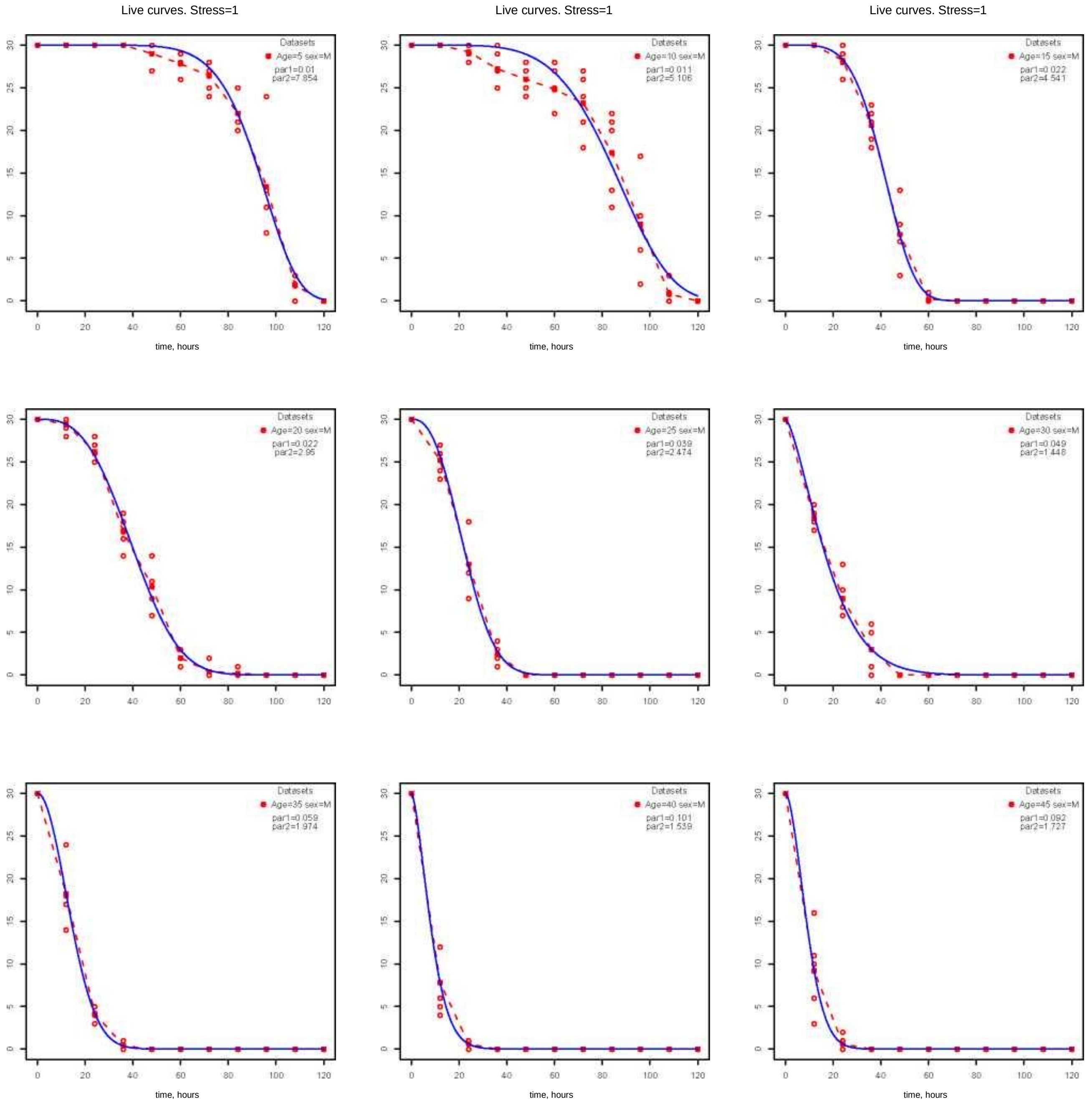
Live curves. Stress=1
Datasets
Age=5 sex=M
par1=0.01
par2=7.854
Live curves. Stress=1
Datasets
Age=10 sex=M
par1=0.011
par2=5.106
Live curves. Stress=1
Datasets
Age=15 sex=M
par1=0.022
par2=4.541
Datasets
Age=20 sex=M
par1=0.022
par2=2.95
Datasets
Age=25 sex=M
par1=0.039
par2=2.474
Datasets
Age=30 sex=M
par1=0.049
par2=1.448
Datasets
Age=35 sex=M
par1=0.059
par2=1.974
Datasets
Age=40 sex=M
par1=0.101
par2=1.539
Datasets
Age=45 sex=M
par1=0.092
par2=1.727
time, hours

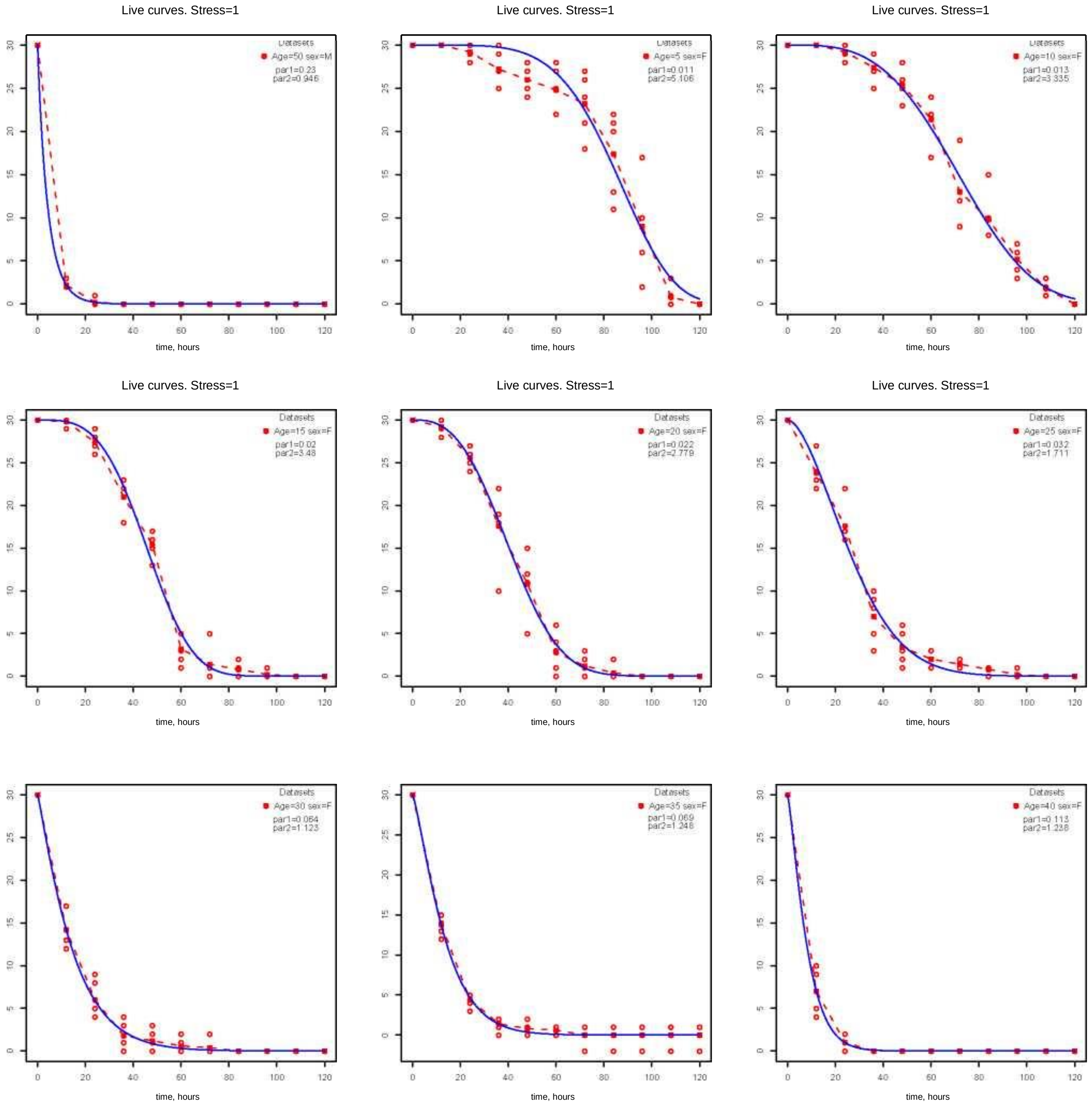
Live curves. Stress=1
Datasets
Age=50 sex=M
par1=0.23
par2=0.946
time, hours
Live curves. Stress=1
Datasets
Age=5 sex=F
par1=0.011
par2=5.106
time, hours
Live curves. Stress=1
Datasets
Age=10 sex=F
par1=0.013
par2=3.335
time, hours
Live curves. Stress=1
Datasets
Age=15 sex=F
par1=0.02
par2=3.48
time, hours
Live curves. Stress=1
Datasets
Age=20 sex=F
par1=0.022
par2=2.779
time, hours
Live curves. Stress=1
Datasets
Age=25 sex=F
par1=0.032
par2=1.711
time, hours
Datasets
Age=30 sex=F
par1=0.064
par2=1.123
time, hours
Datasets
Age=35 sex=F
par1=0.069
par2=1.248
time, hours
Datasets
Age=40 sex=F
par1=0.113
par2=1.238
time, hours

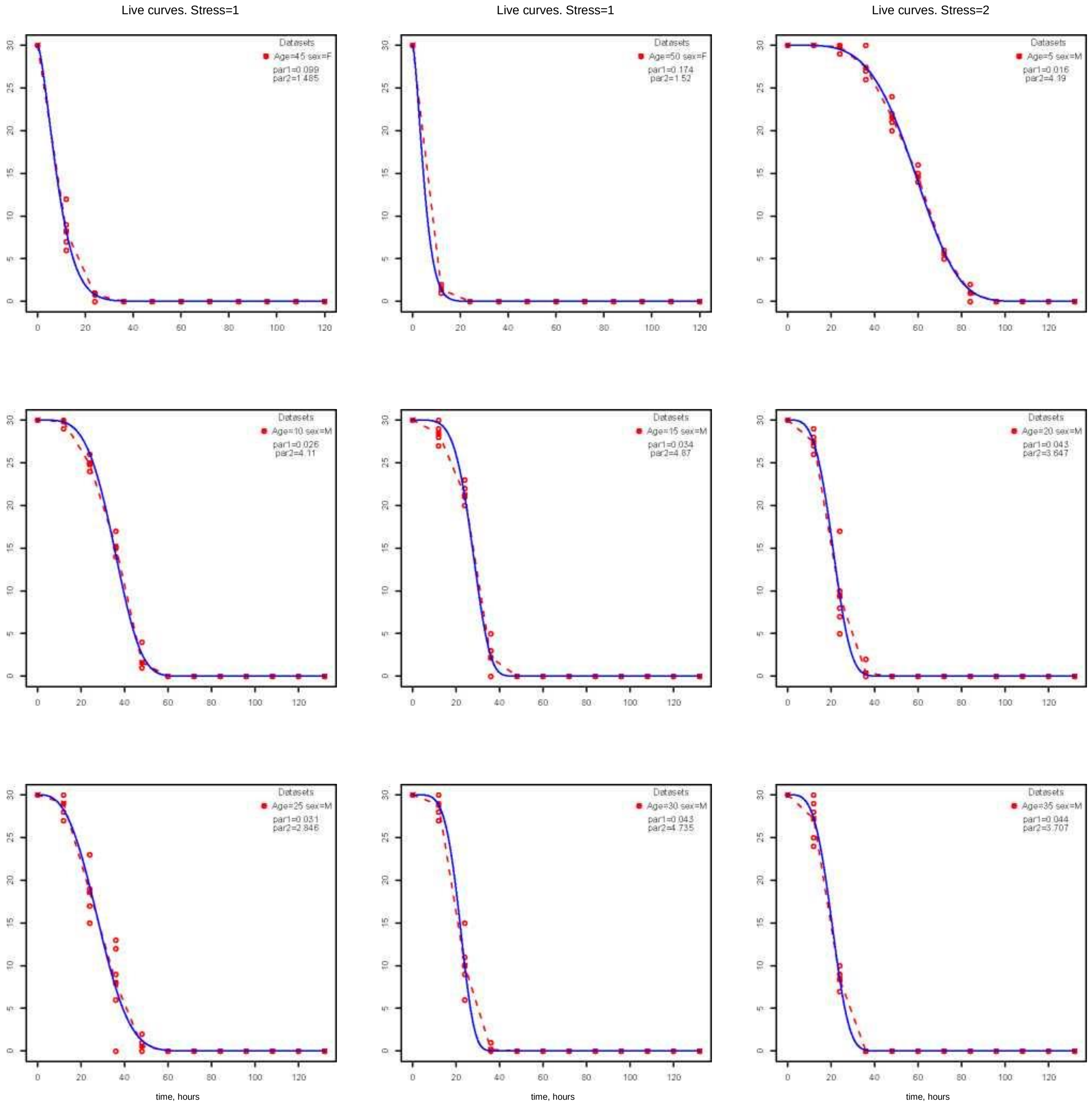

Live curves. Stress=1
Live curves. Stress=1
Live curves. Stress=2
Datasets
Age=45 sex=F
par1=0.099
par2=1.485
Datasets
Age=50 sex=F
par1=0.174
par2=1.52
Datasets
Age=5 sex=M
par1=0.016
par2=4.19
Datasets
Age=10 sex=M
par1=0.026
par2=4.11
Datasets
Age=15 sex=M
par1=0.034
par2=4.87
Datasets
Age=20 sex=M
par1=0.043
par2=3.647
Datasets
Age=25 sex=M
par1=0.031
par2=2.846
Datasets
Age=30 sex=M
par1=0.043
par2=4.735
Datasets
Age=35 sex=M
par1=0.044
par2=3.707
time, hours
time, hours
time, hours

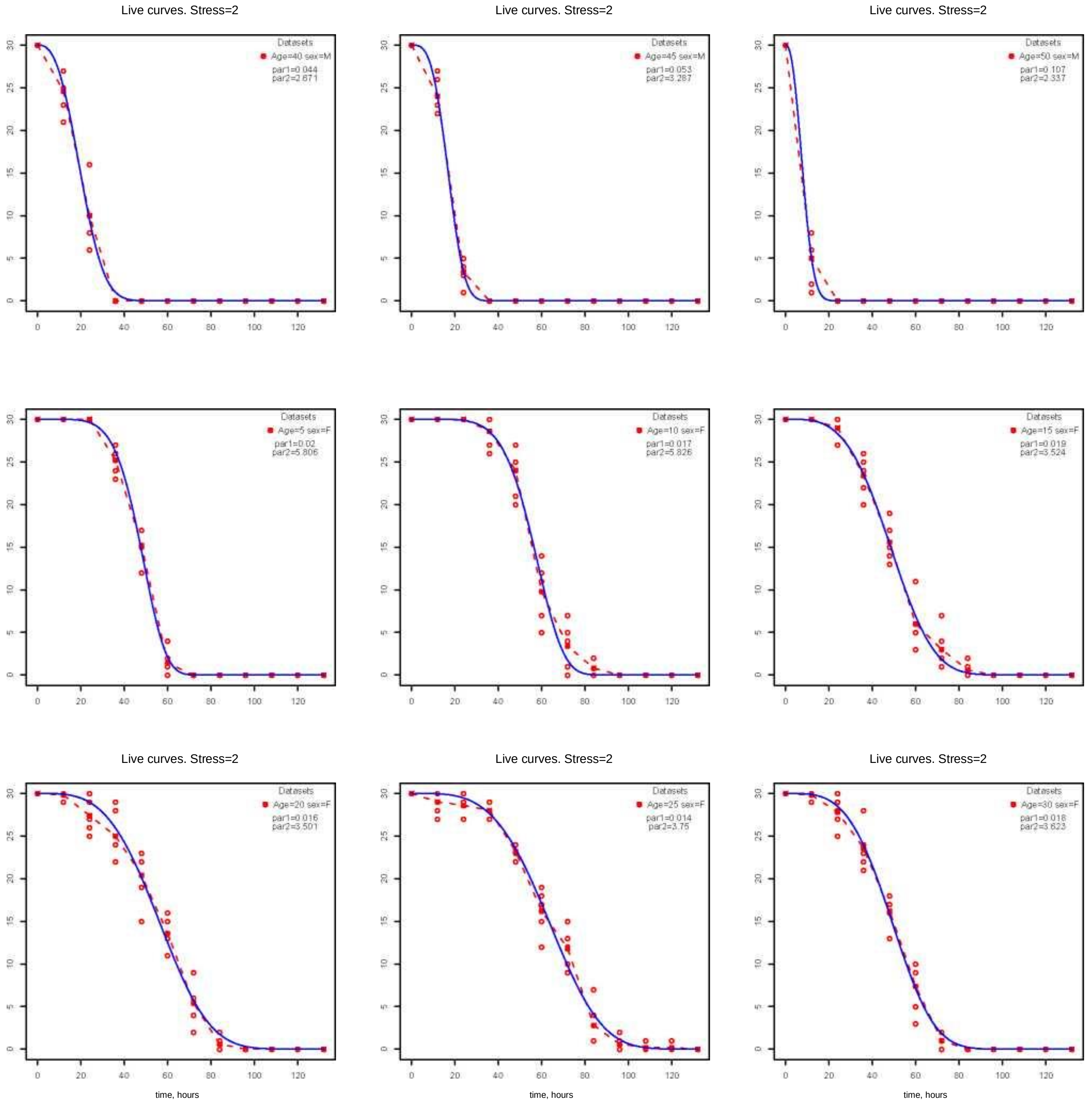

Live curves. Stress=2
Datasets
Age=40 sex=M
par1=0.044
par2=2.671
Live curves. Stress=2
Datasets
Age=45 sex=M
par1=0.053
par2=3.287
Live curves. Stress=2
Datasets
Age=50 sex=M
par1=0.107
par2=2.337
Datasets
Age=5 sex=F
par1=0.02
par2=5.806
Datasets
Age=10 sex=F
par1=0.017
par2=5.826
Datasets
Age=15 sex=F
par1=0.019
par2=3.524
Live curves. Stress=2
Datasets
Age=20 sex=F
par1=0.016
par2=3.501
time, hours
Live curves. Stress=2
Datasets
Age=25 sex=F
par1=0.014
par2=3.75
time, hours
Live curves. Stress=2
Datasets
Age=30 sex=F
par1=0.018
par2=3.623
time, hours

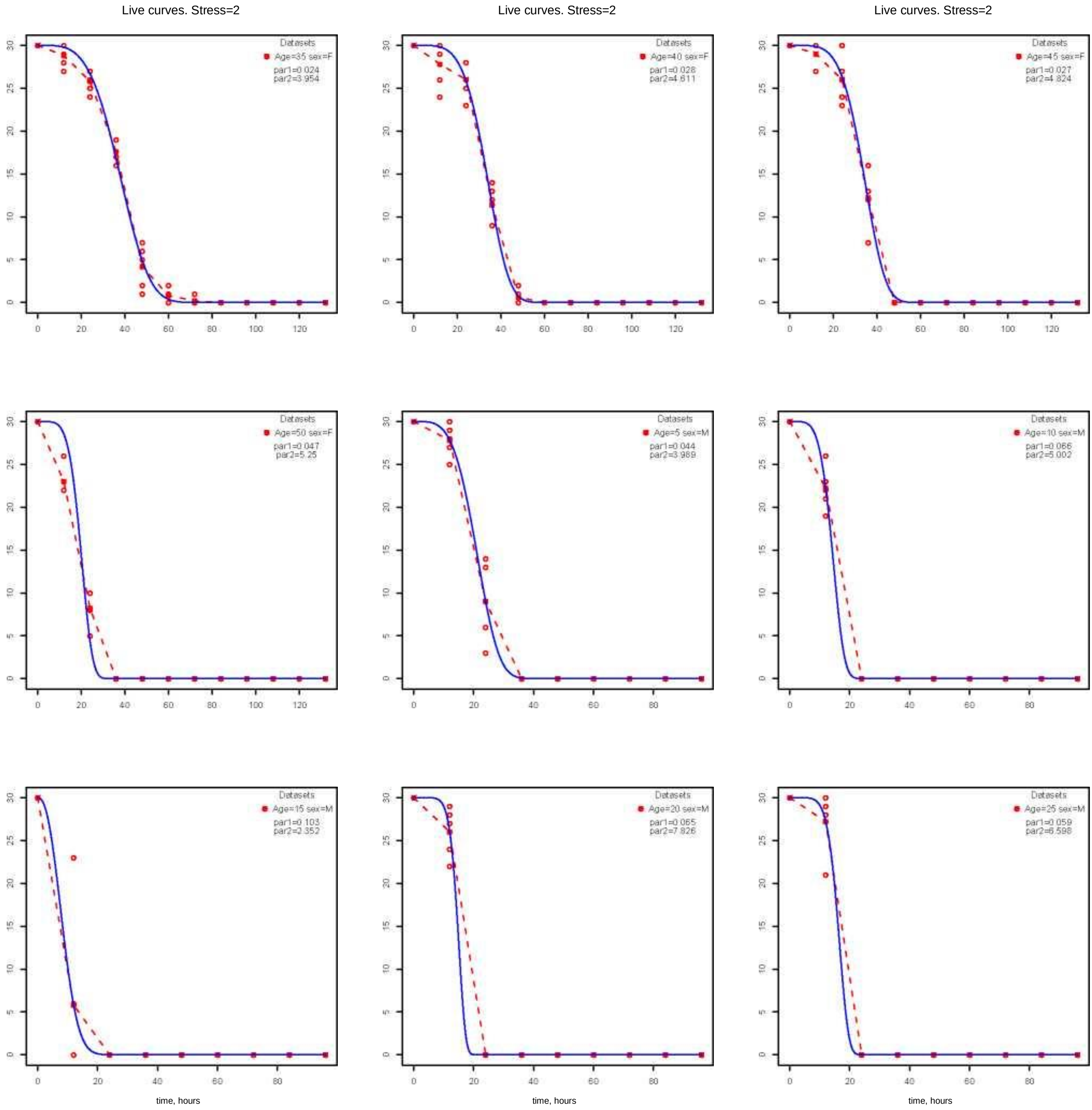
Live curves. Stress=2
Live curves. Stress=2
Live curves. Stress=2
Datasets
Age=35 sex=F
par1=0.024
par2=3.954
Datasets
Age=40 sex=F
par1=0.028
par2=4.611
Datasets
Age=45 sex=F
par1=0.027
par2=4.824
Datasets
Age=50 sex=F
par1=0.047
par2=5.25
Datasets
Age=5 sex=M
par1=0.044
par2=3.989
Datasets
Age=10 sex=M
par1=0.066
par2=5.002
Datasets
Age=15 sex=M
par1=0.103
par2=2.352
Datasets
Age=20 sex=M
par1=0.065
par2=7.826
Datasets
Age=25 sex=M
par1=0.059
par2=6.598
time, hours
time, hours
time, hours

Live curves. Stress=3

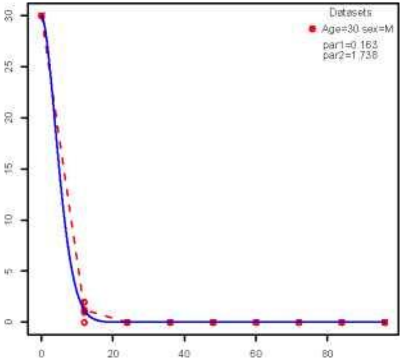

Live curves. Stress=3

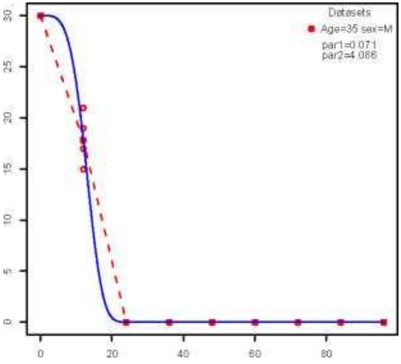

Live curves. Stress=3

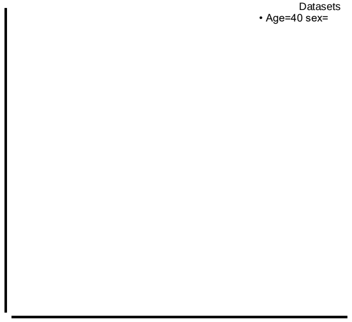

Live curves. Stress=3

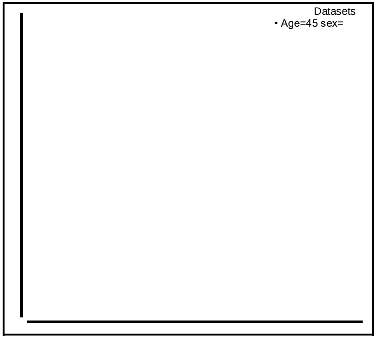

Live curves. Stress=3

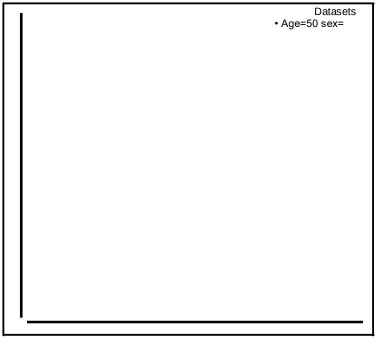

Live curves. Stress=3

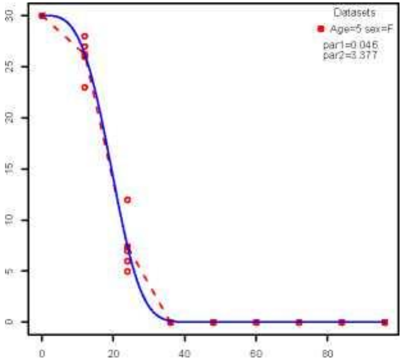

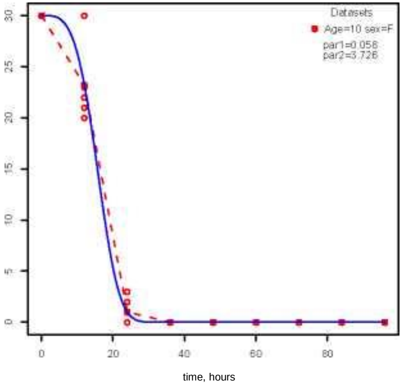

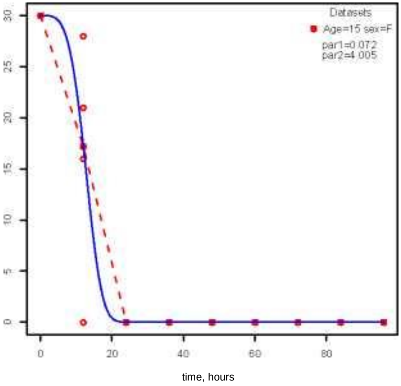

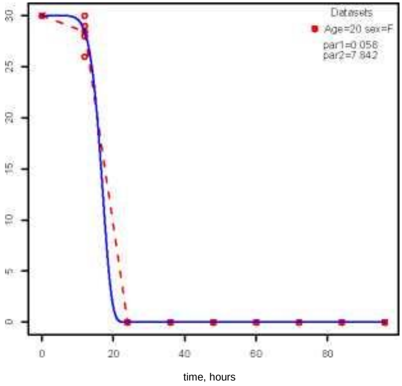

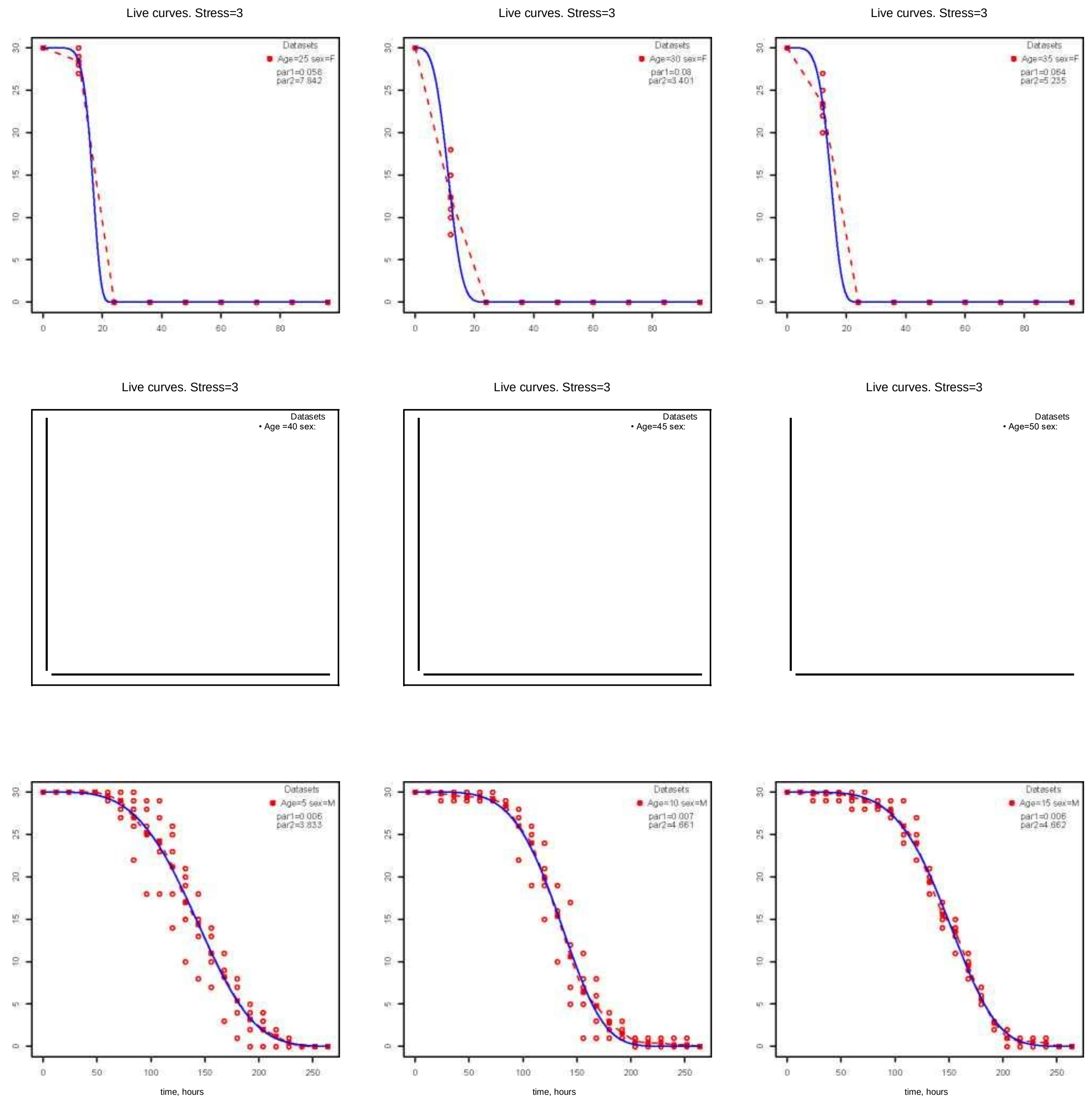
Live curves. Stress=3
Datasets
Age=25 sex=F
par1=0.058
par2=7.842
Live curves. Stress=3
Datasets
Age=30 sex=F
par1=0.08
par2=3.401
Live curves. Stress=3
Datasets
Age=35 sex=F
par1=0.064
par2=5.235
Live curves. Stress=3
Datasets
• Age =40 sex:
Live curves. Stress=3
Datasets
• Age=45 sex:
Live curves. Stress=3
Datasets
• Age=50 sex:
Datasets
Age=5 sex=M
par1=0.006
par2=3.833
time, hours
Datasets
Age=10 sex=M
par1=0.007
par2=4.661
time, hours
Datasets
Age=15 sex=M
par1=0.006
par2=4.662
time, hours

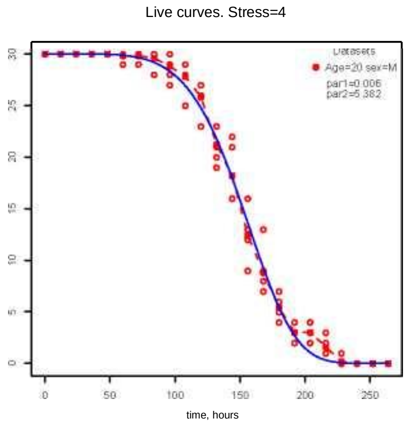
Live curves. Stress=4
Datasets
Age=20 sex=M
par1=0.006
par2=5.382
time, hours

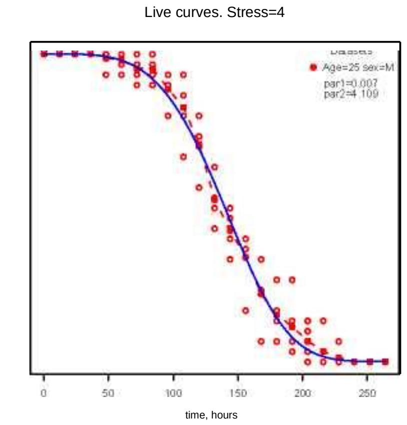
Live curves. Stress=4
Datasets
Age=25 sex=M
par1=0.007
par2=4.109
time, hours

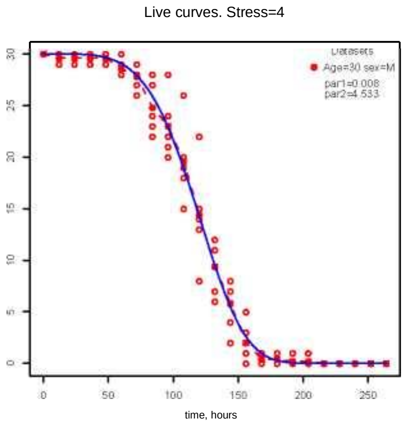
Live curves. Stress=4
Datasets
Age=30 sex=M
par1=0.008
par2=4.533
time, hours

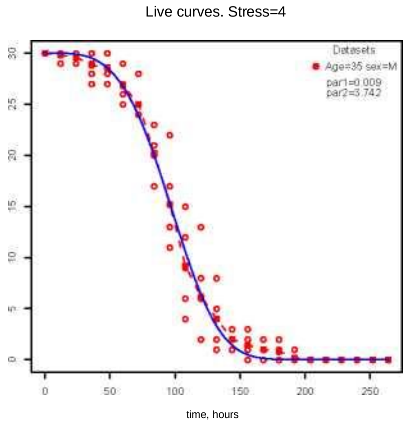
Live curves. Stress=4
Datasets
Age=35 sex=M
par1=0.009
par2=3.742
time, hours

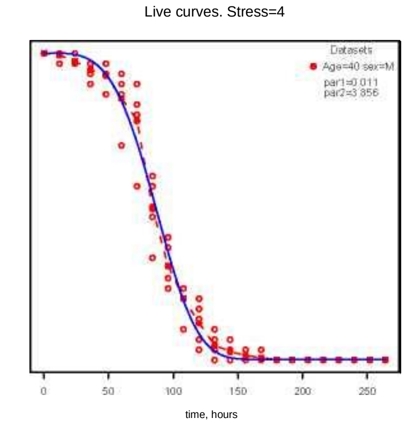
Live curves. Stress=4
Datasets
Age=40 sex=M
par1=0.011
par2=3.856
time, hours

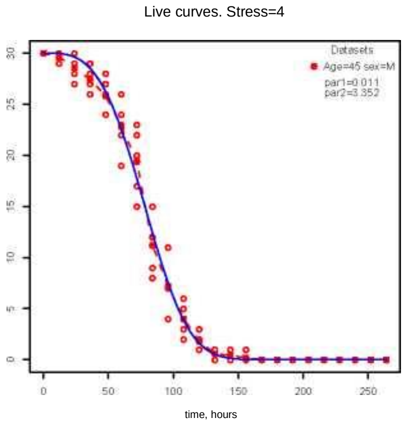
Live curves. Stress=4
Datasets
Age=45 sex=M
par1=0.011
par2=3.352
time, hours

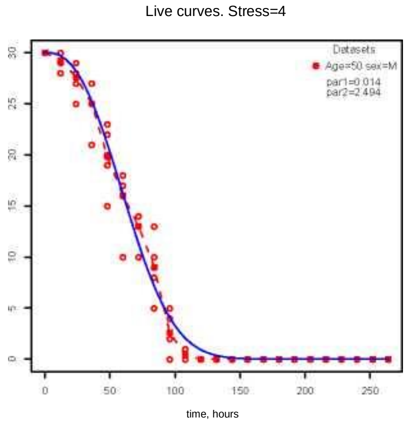
Live curves. Stress=4
Datasets
Age=50 sex=M
par1=0.014
par2=2.494
time, hours

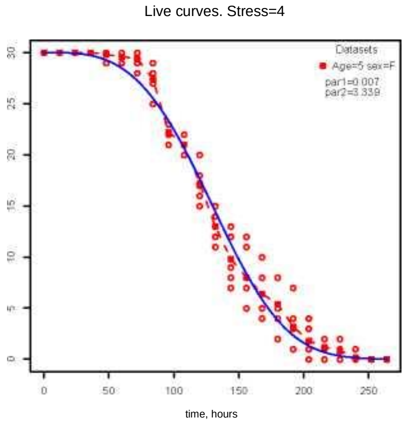
Live curves. Stress=4
Datasets
Age=5 sex=F
par1=0.007
par2=3.339
time, hours

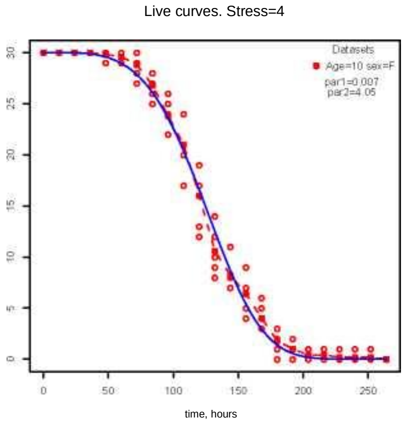
Live curves. Stress=4
Datasets
Age=10 sex=F
par1=0.007
par2=4.05
time, hours

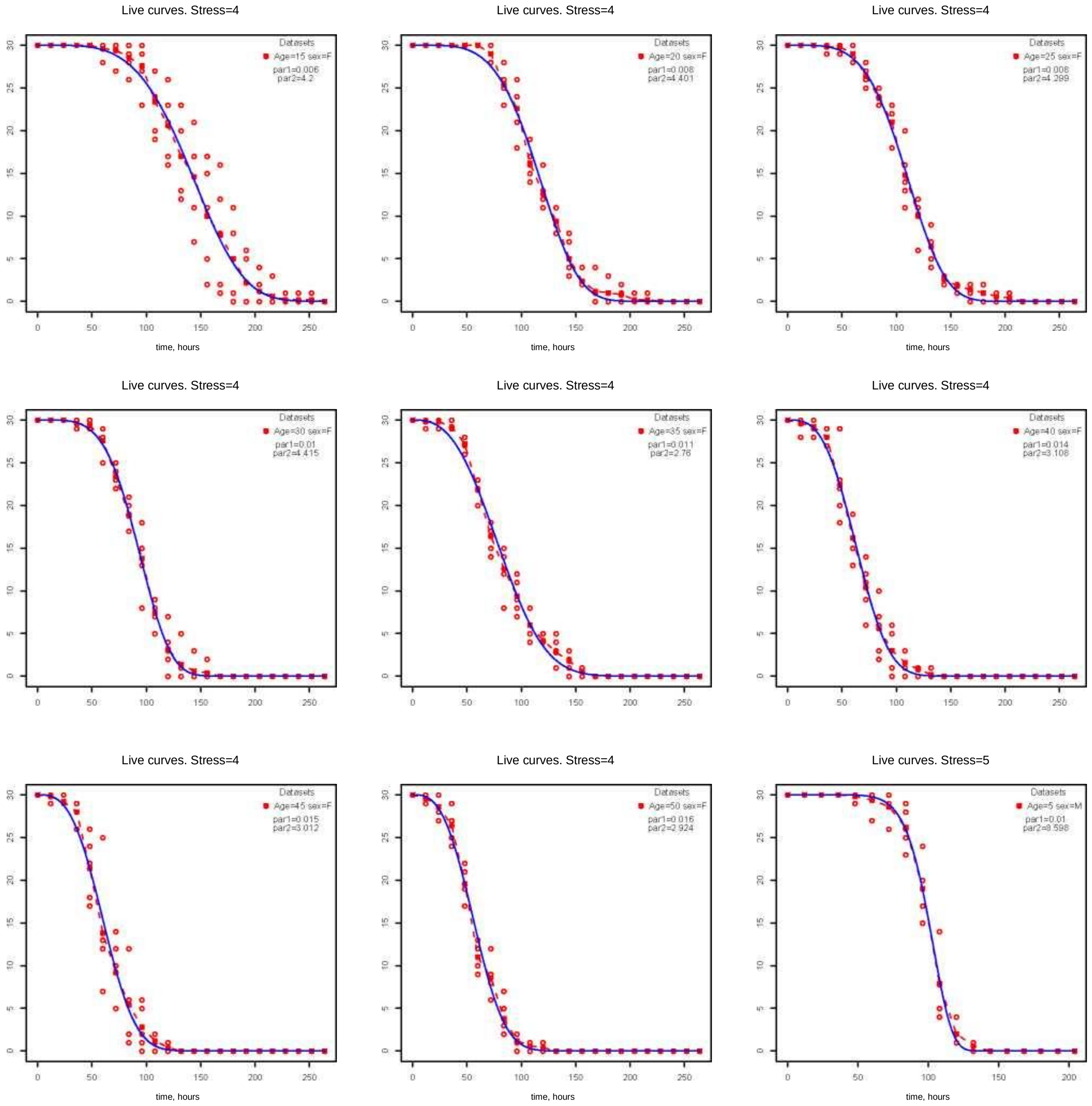

Live curves. Stress=4
Datasets
Age=15 sex=F
par1=0.006
par2=4.2
time, hours
Live curves. Stress=4
Datasets
Age=20 sex=F
par1=0.008
par2=4.401
time, hours
Live curves. Stress=4
Datasets
Age=25 sex=F
par1=0.008
par2=4.299
time, hours
Live curves. Stress=4
Datasets
Age=30 sex=F
par1=0.01
par2=4.415
Live curves. Stress=4
Datasets
Age=35 sex=F
par1=0.011
par2=2.76
Live curves. Stress=4
Datasets
Age=40 sex=F
par1=0.014
par2=3.108
Live curves. Stress=4
Datasets
Age=45 sex=F
par1=0.015
par2=3.012
time, hours
Live curves. Stress=4
Datasets
Age=50 sex=F
par1=0.016
par2=2.924
time, hours
Live curves. Stress=5
Datasets
Age=5 sex=M
par1=0.01
par2=8.598
time, hours

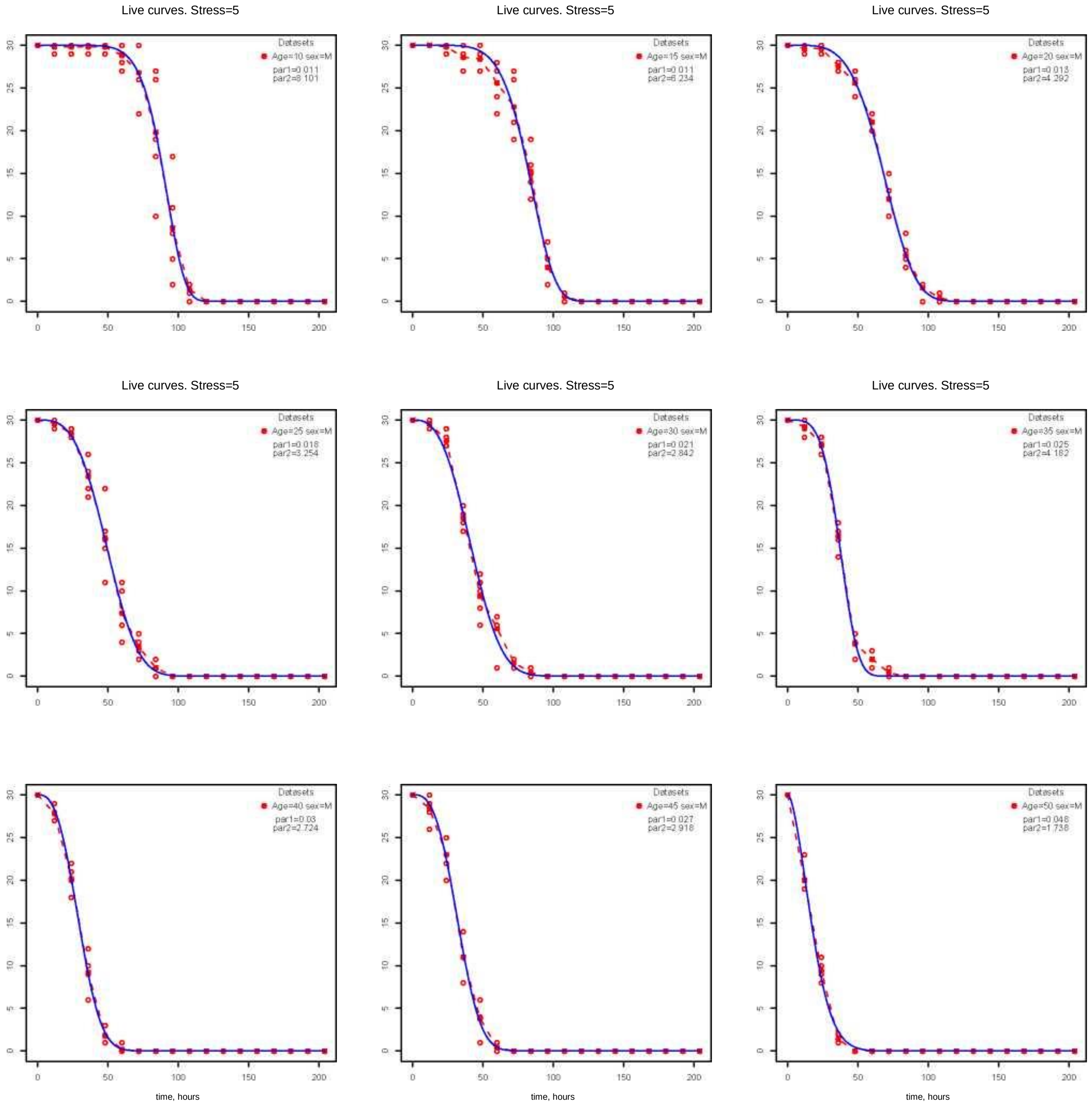
Live curves. Stress=5
Datasets
Age=10 sex=M
par1=0.011
par2=8.101
Live curves. Stress=5
Datasets
Age=15 sex=M
par1=0.011
par2=6.234
Live curves. Stress=5
Datasets
Age=20 sex=M
par1=0.013
par2=4.292
Live curves. Stress=5
Datasets
Age=25 sex=M
par1=0.018
par2=3.254
Live curves. Stress=5
Datasets
Age=30 sex=M
par1=0.021
par2=2.842
Live curves. Stress=5
Datasets
Age=35 sex=M
par1=0.025
par2=4.182
Datasets
Age=40 sex=M
par1=0.03
par2=2.724
Datasets
Age=45 sex=M
par1=0.027
par2=2.918
Datasets
Age=50 sex=M
par1=0.048
par2=1.738
time, hours
time, hours
time, hours

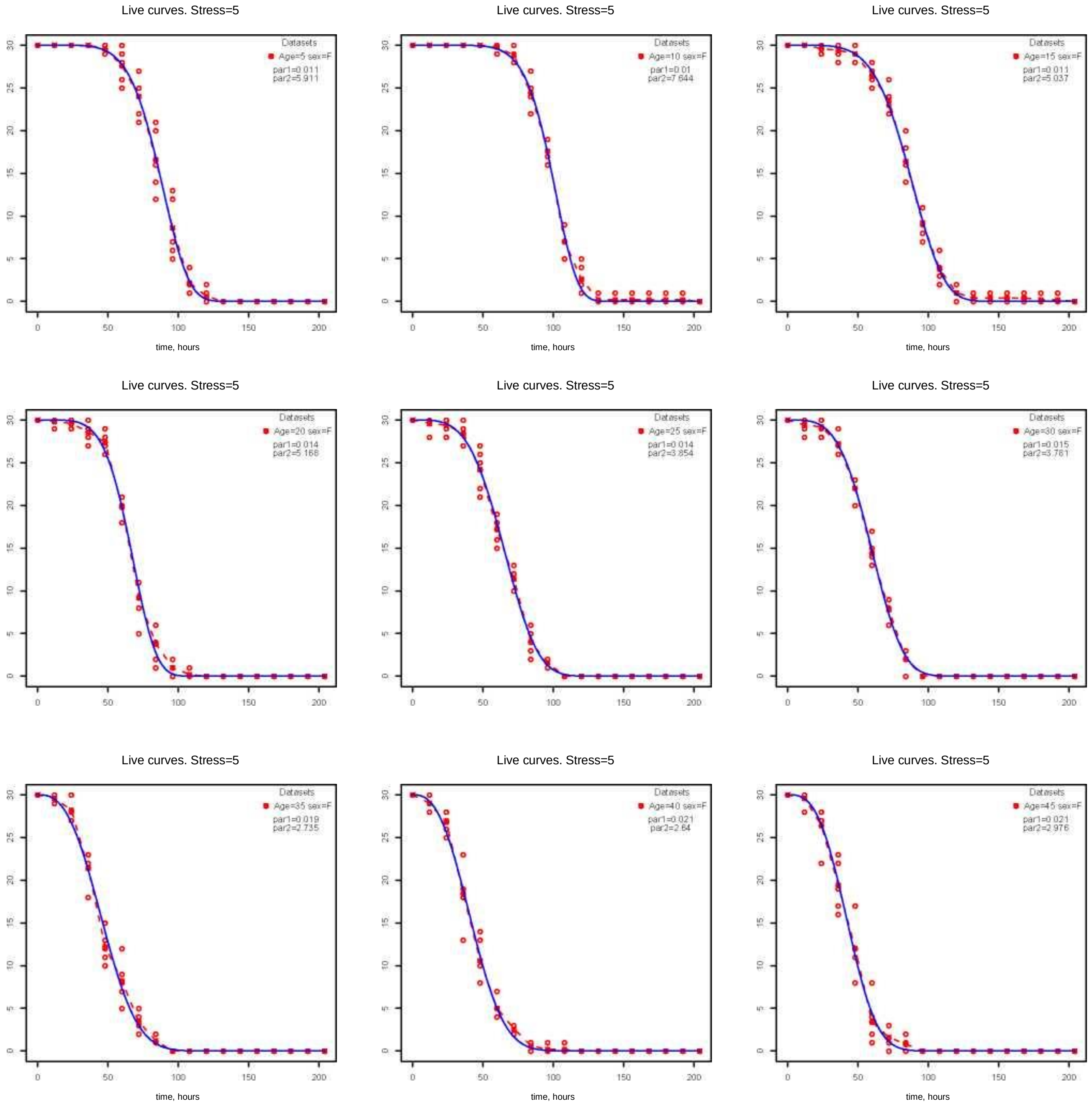
Live curves. Stress=5
Datasets
Age=5 sex=F
par1=0.011
par2=5.911
time, hours
Live curves. Stress=5
Datasets
Age=10 sex=F
par1=0.01
par2=7.644
time, hours
Live curves. Stress=5
Datasets
Age=15 sex=F
par1=0.011
par2=5.037
time, hours
Live curves. Stress=5
Datasets
Age=20 sex=F
par1=0.014
par2=5.166
Live curves. Stress=5
Datasets
Age=25 sex=F
par1=0.014
par2=3.854
Live curves. Stress=5
Datasets
Age=30 sex=F
par1=0.015
par2=3.761
Live curves. Stress=5
Datasets
Age=35 sex=F
par1=0.019
par2=2.735
time, hours
Live curves. Stress=5
Datasets
Age=40 sex=F
par1=0.021
par2=2.64
time, hours
Live curves. Stress=5
Datasets
Age=45 sex=F
par1=0.021
par2=2.976
time, hours

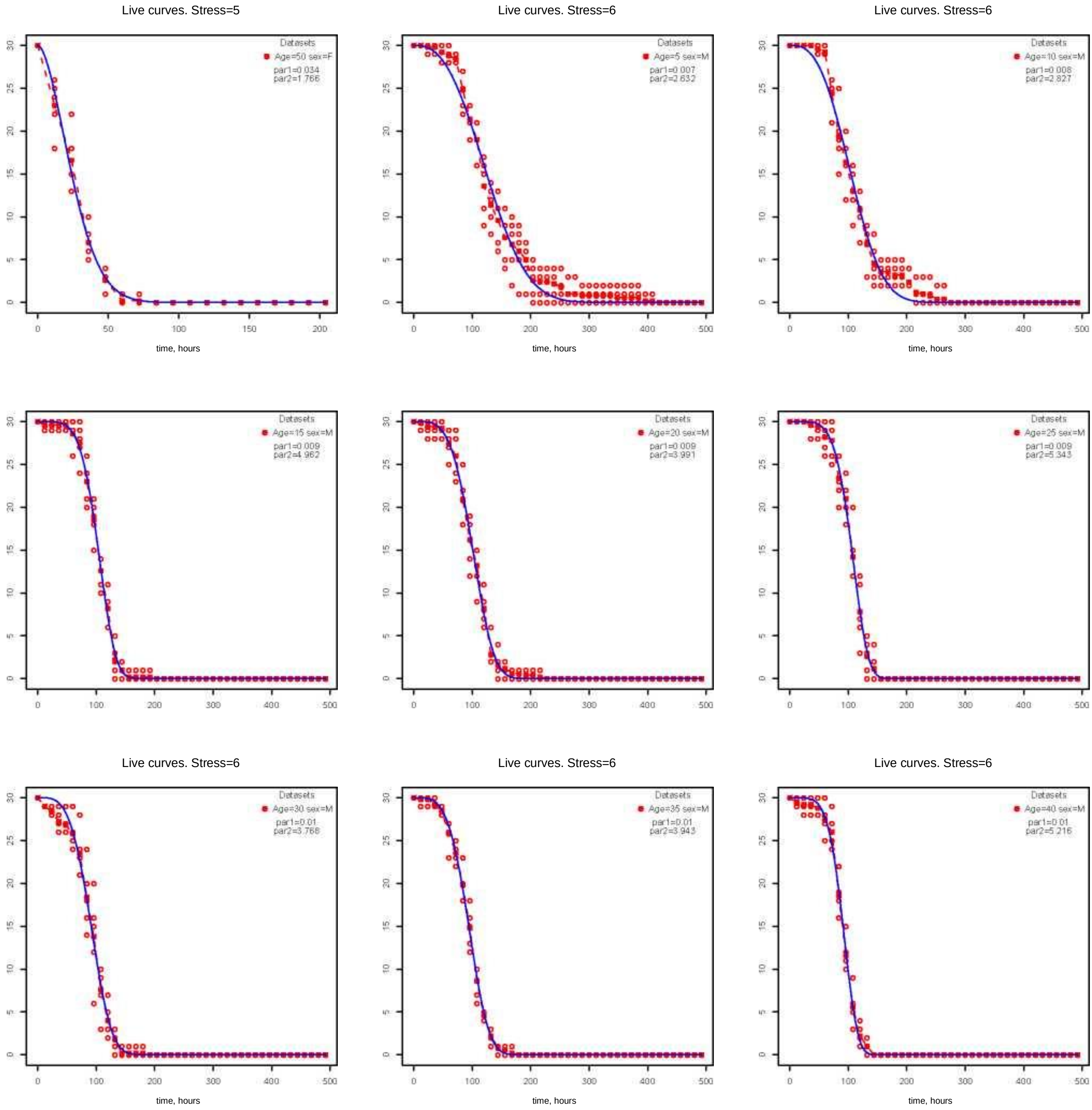
Live curves. Stress=5
Datasets
Age=50 sex=F
par1=0.034
par2=1.766
time, hours
Live curves. Stress=6
Datasets
Age=5 sex=M
par1=0.007
par2=2.632
time, hours
Live curves. Stress=6
Datasets
Age=10 sex=M
par1=0.008
par2=2.827
time, hours
Datasets
Age=15 sex=M
par1=0.009
par2=4.962
Datasets
Age=20 sex=M
par1=0.009
par2=3.991
Datasets
Age=25 sex=M
par1=0.009
par2=5.343
Live curves. Stress=6
Datasets
Age=30 sex=M
par1=0.01
par2=3.768
time, hours
Live curves. Stress=6
Datasets
Age=35 sex=M
par1=0.01
par2=3.943
time, hours
Live curves. Stress=6
Datasets
Age=40 sex=M
par1=0.01
par2=5.216
time, hours

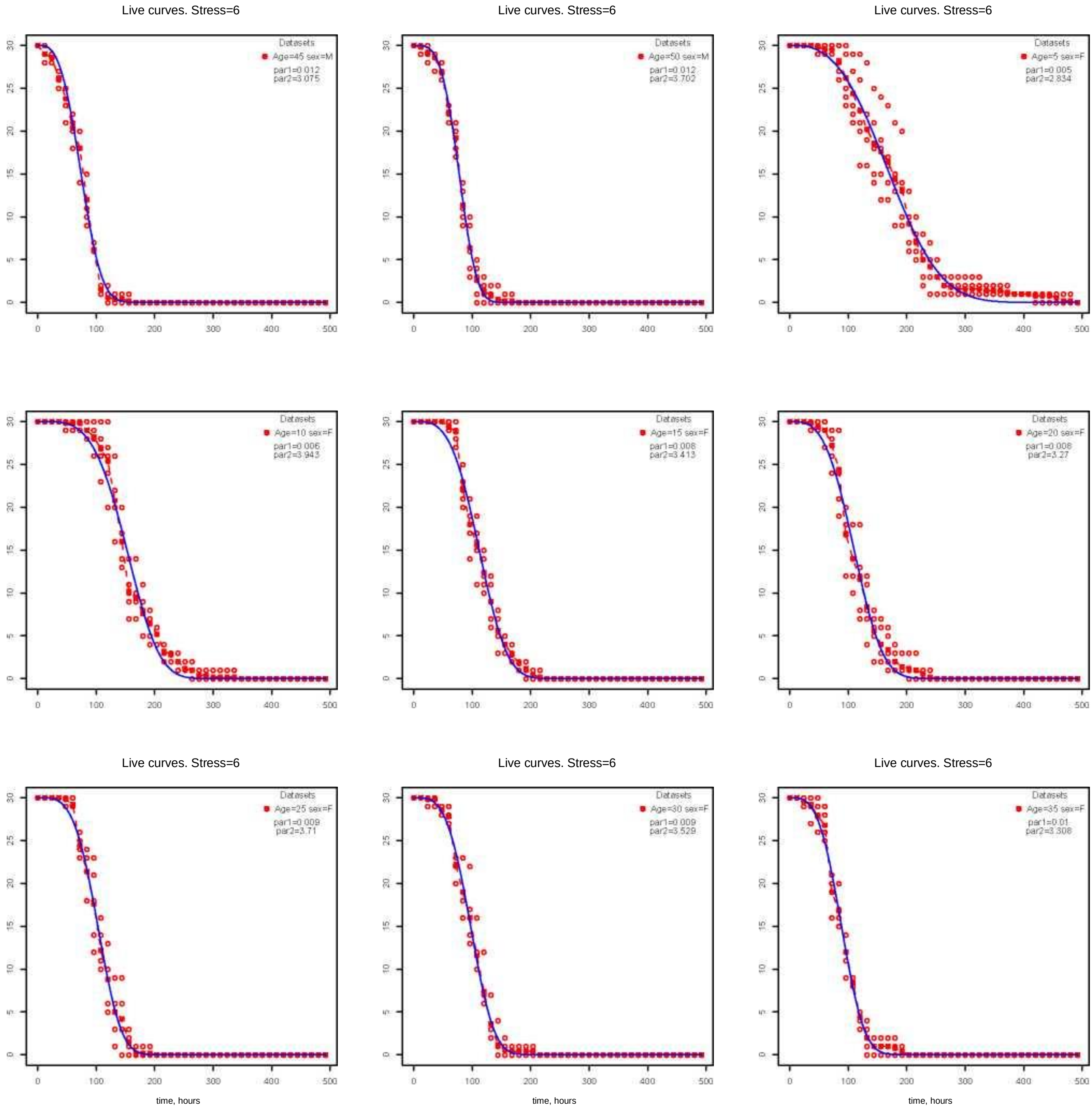
Live curves. Stress=6
Datasets
Age=45 sex=M
par1=0.012
par2=3.075
Live curves. Stress=6
Datasets
Age=50 sex=M
par1=0.012
par2=3.702
Live curves. Stress=6
Datasets
Age=5 sex=F
par1=0.005
par2=2.834
Datasets
Age=10 sex=F
par1=0.006
par2=3.943
Datasets
Age=15 sex=F
par1=0.008
par2=3.413
Datasets
Age=20 sex=F
par1=0.008
par2=3.27
Live curves. Stress=6
Datasets
Age=25 sex=F
par1=0.009
par2=3.71
time, hours
Live curves. Stress=6
Datasets
Age=30 sex=F
par1=0.009
par2=3.529
time, hours
Live curves. Stress=6
Datasets
Age=35 sex=F
par1=0.01
par2=3.308
time, hours

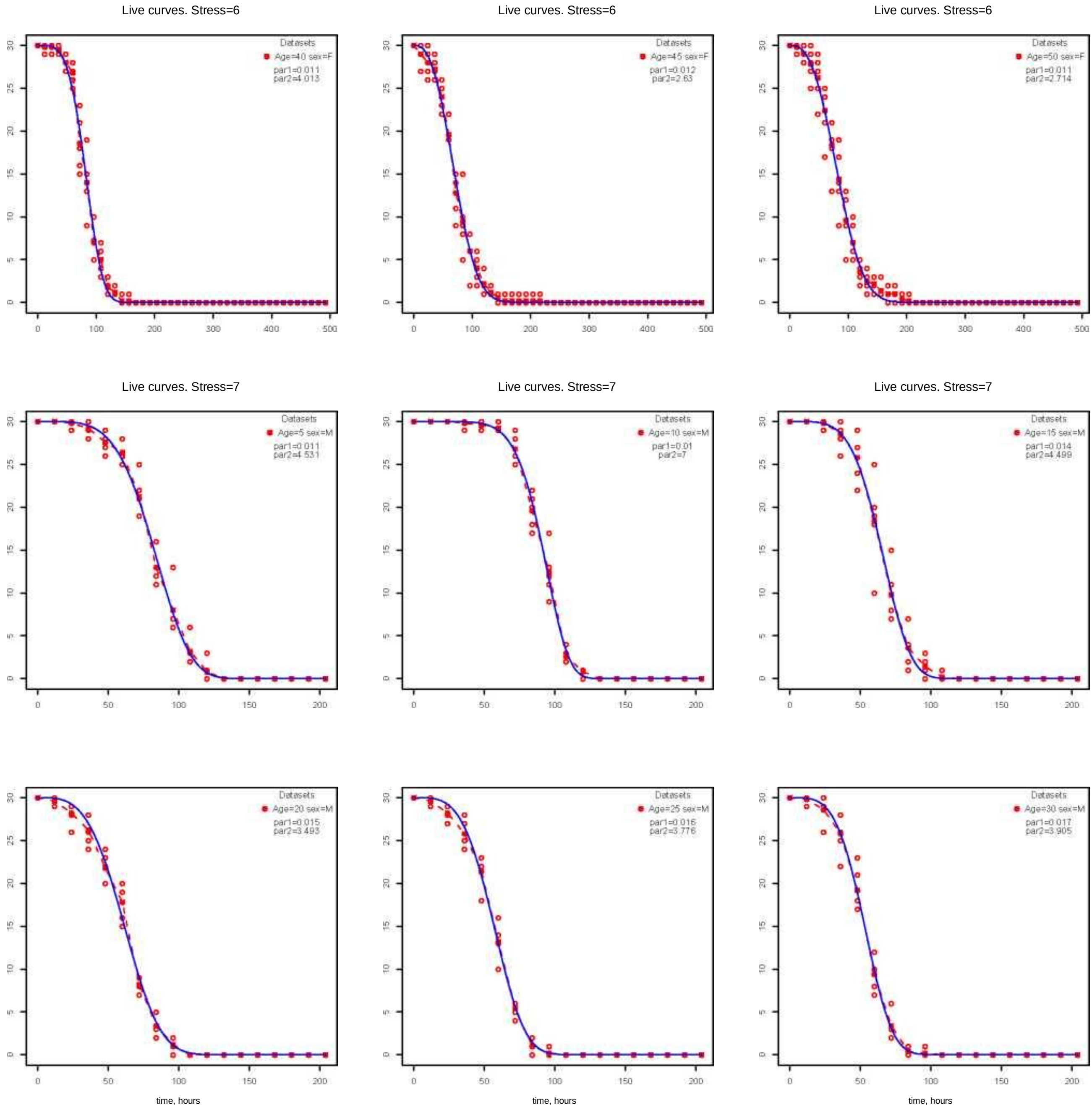

Live curves. Stress=6
Datasets
Age=40 sex=F
par1=0.011
par2=4.013
Live curves. Stress=6
Datasets
Age=45 sex=F
par1=0.012
par2=2.63
Live curves. Stress=6
Datasets
Age=50 sex=F
par1=0.011
par2=2.714
Live curves. Stress=7
Datasets
Age=5 sex=M
par1=0.011
par2=4.531
Live curves. Stress=7
Datasets
Age=10 sex=M
par1=0.01
par2=7
Live curves. Stress=7
Datasets
Age=15 sex=M
par1=0.014
par2=4.499
Datasets
Age=20 sex=M
par1=0.015
par2=3.493
Datasets
Age=25 sex=M
par1=0.016
par2=3.776
Datasets
Age=30 sex=M
par1=0.017
par2=3.905
time, hours
time, hours
time, hours

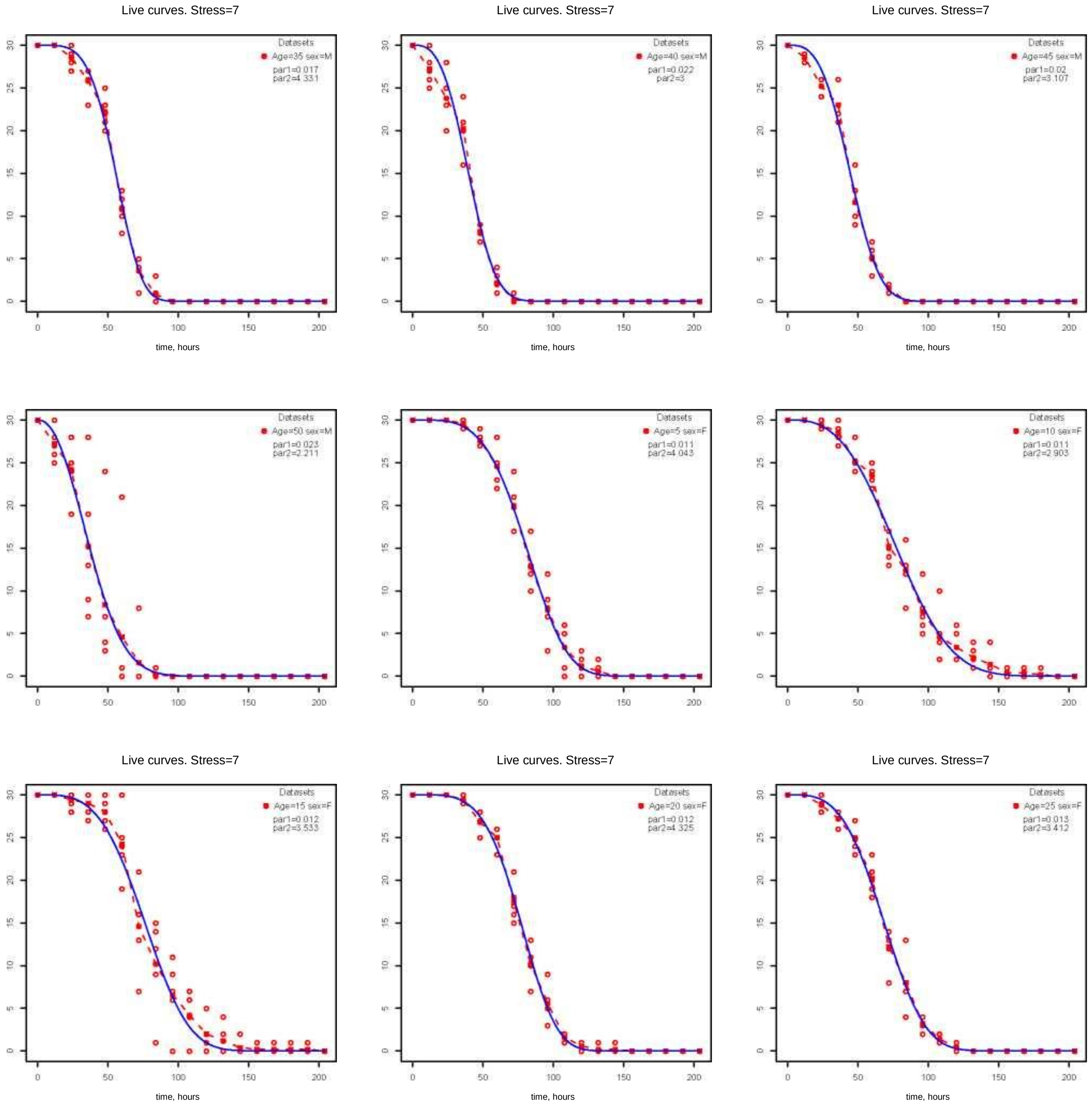

Live curves. Stress=7
Datasets
Age=35 sex=M
par1=0.017
par2=4.331
time, hours
Live curves. Stress=7
Datasets
Age=40 sex=M
par1=0.022
par2=3
time, hours
Live curves. Stress=7
Datasets
Age=45 sex=M
par1=0.02
par2=3.107
time, hours
Datasets
Age=50 sex=M
par1=0.023
par2=2.211
Datasets
Age=5 sex=F
par1=0.011
par2=4.043
Datasets
Age=10 sex=F
par1=0.011
par2=2.903
Live curves. Stress=7
Datasets
Age=15 sex=F
par1=0.012
par2=3.533
time, hours
Live curves. Stress=7
Datasets
Age=20 sex=F
par1=0.012
par2=4.325
time, hours
Live curves. Stress=7
Datasets
Age=25 sex=F
par1=0.013
par2=3.412
time, hours

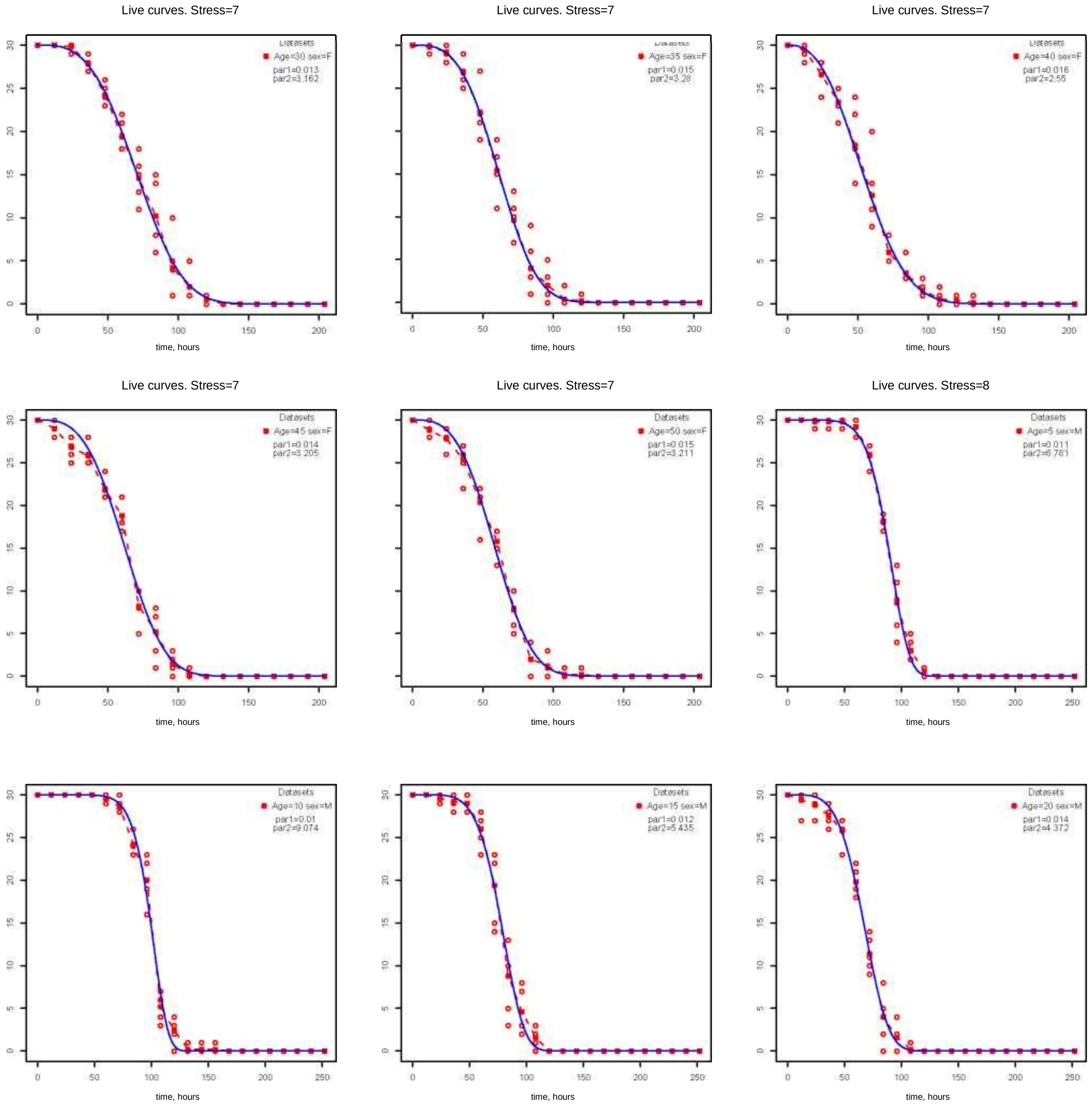

Live curves. Stress=7
Datasets
Age=30 sex=F
par1=0.013
par2=3.162
time, hours
Live curves. Stress=7
Datasets
Age=35 sex=F
par1=0.015
par2=3.28
time, hours
Live curves. Stress=7
Datasets
Age=40 sex=F
par1=0.016
par2=2.55
time, hours
Live curves. Stress=7
Datasets
Age=45 sex=F
par1=0.014
par2=3.205
time, hours
Live curves. Stress=7
Datasets
Age=50 sex=F
par1=0.015
par2=3.211
time, hours
Live curves. Stress=8
Datasets
Age=5 sex=M
par1=0.011
par2=6.781
time, hours
Datasets
Age=10 sex=M
par1=0.01
par2=9.074
time, hours
Datasets
Age=15 sex=M
par1=0.012
par2=5.435
time, hours
Datasets
Age=20 sex=M
par1=0.014
par2=4.372
time, hours

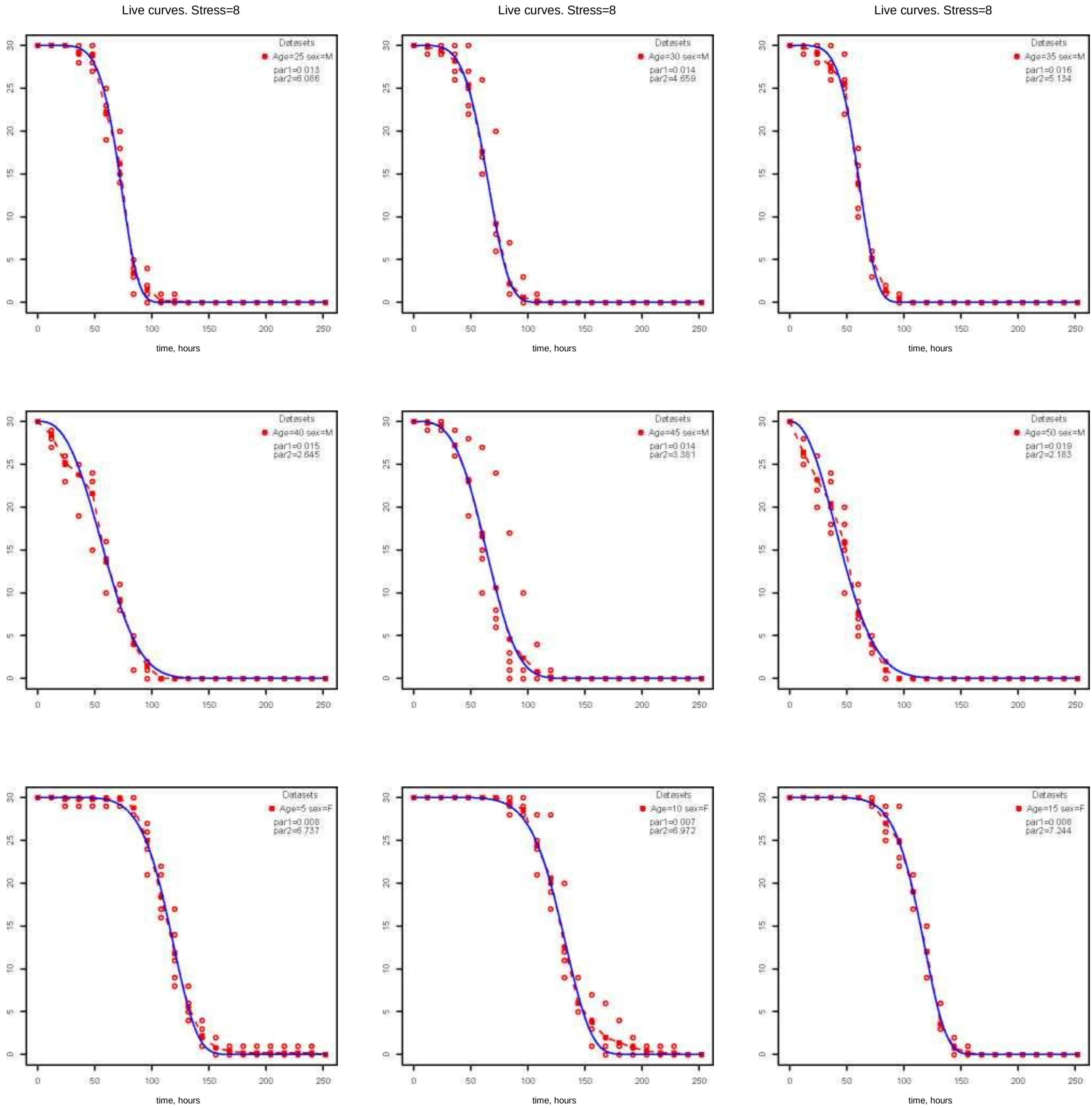
Live curves. Stress=8
Datasets
Age=25 sex=M
par1=0.013
par2=6.086
time, hours
Live curves. Stress=8
Datasets
Age=30 sex=M
par1=0.014
par2=4.659
time, hours
Live curves. Stress=8
Datasets
Age=35 sex=M
par1=0.016
par2=5.134
time, hours
Datasets
Age=40 sex=M
par1=0.015
par2=2.645
Datasets
Age=45 sex=M
par1=0.014
par2=3.381
Datasets
Age=50 sex=M
par1=0.019
par2=2.183
Datasets
Age=5 sex=F
par1=0.008
par2=6.737
time, hours
Datasets
Age=10 sex=F
par1=0.007
par2=6.972
time, hours
Datasets
Age=15 sex=F
par1=0.008
par2=7.244
time, hours

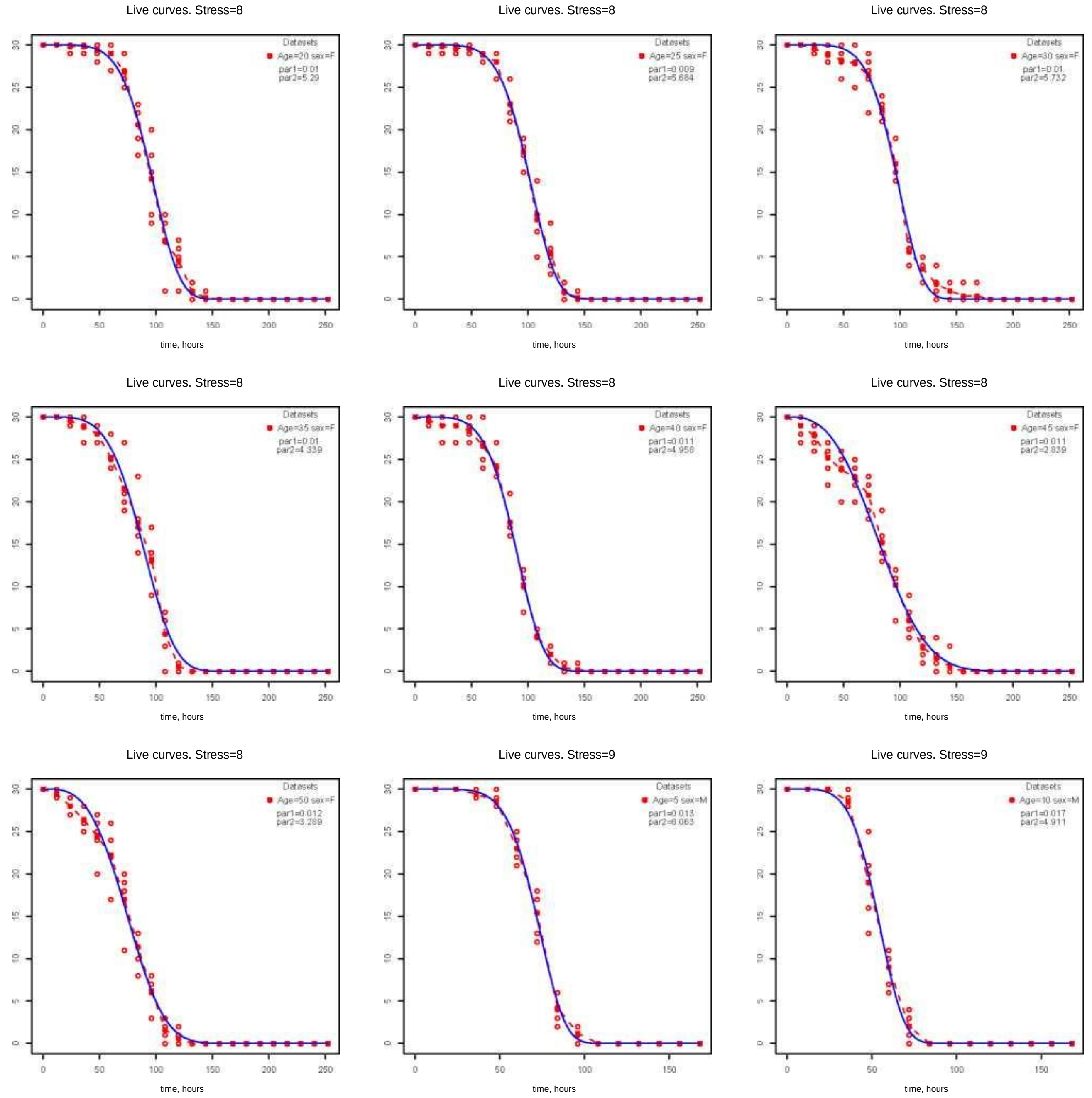
Live curves. Stress=8
Datasets
Age=20 sex=F
par1=0.01
par2=5.29
time, hours
Live curves. Stress=8
Datasets
Age=25 sex=F
par1=0.009
par2=5.684
time, hours
Live curves. Stress=8
Datasets
Age=30 sex=F
par1=0.01
par2=5.732
time, hours
Live curves. Stress=8
Datasets
Age=35 sex=F
par1=0.01
par2=4.339
time, hours
Live curves. Stress=8
Datasets
Age=40 sex=F
par1=0.011
par2=4.958
time, hours
Live curves. Stress=8
Datasets
Age=45 sex=F
par1=0.011
par2=2.839
time, hours
Live curves. Stress=8
Datasets
Age=50 sex=F
par1=0.012
par2=3.289
time, hours
Live curves. Stress=9
Datasets
Age=5 sex=M
par1=0.013
par2=6.063
time, hours
Live curves. Stress=9
Datasets
Age=10 sex=M
par1=0.017
par2=4.911
time, hours

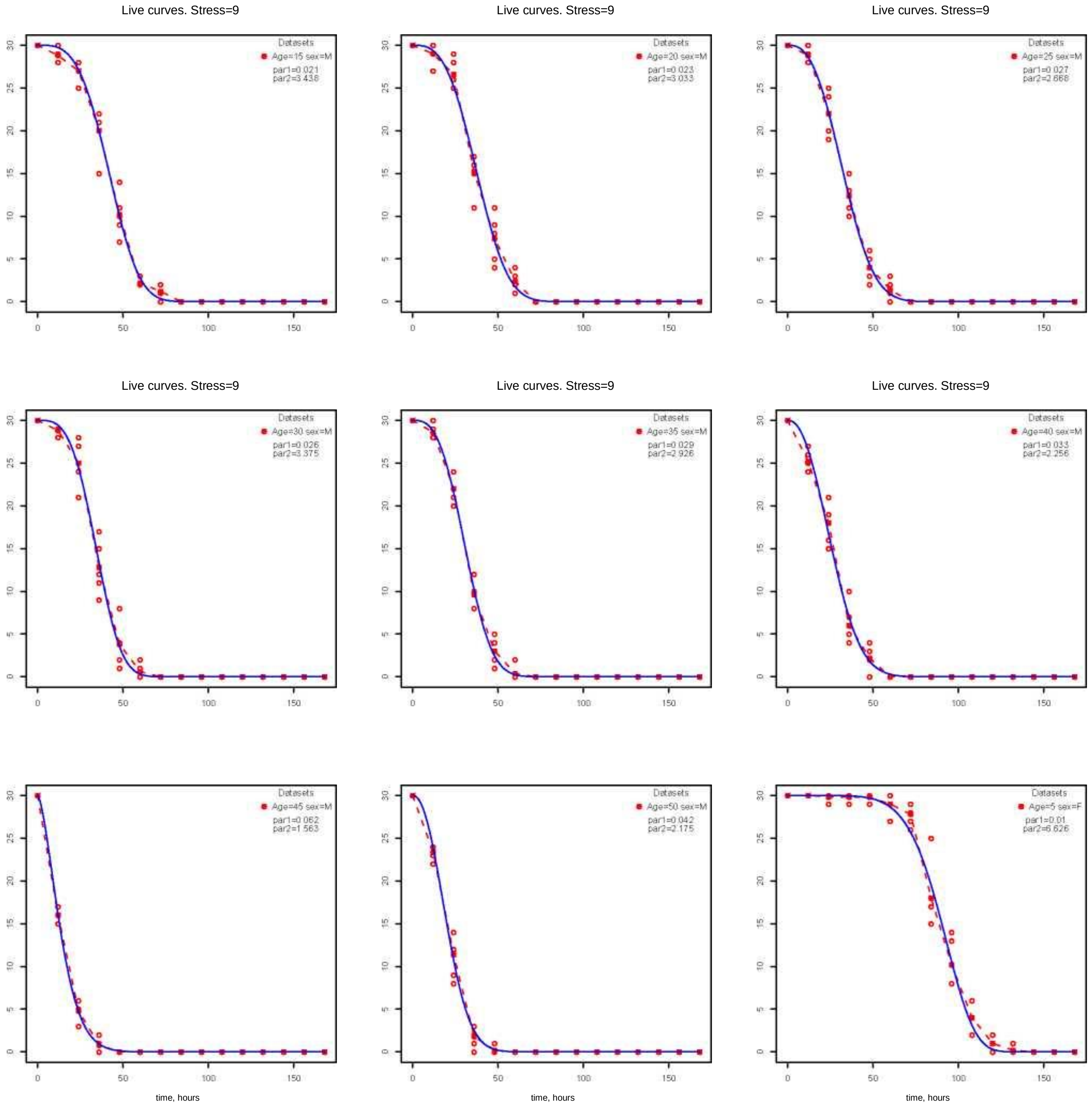
Live curves. Stress=9
Datasets
Age=15 sex=M
par1=0.021
par2=3.438
Live curves. Stress=9
Datasets
Age=20 sex=M
par1=0.023
par2=3.033
Live curves. Stress=9
Datasets
Age=25 sex=M
par1=0.027
par2=2.668
Live curves. Stress=9
Datasets
Age=30 sex=M
par1=0.026
par2=3.375
Live curves. Stress=9
Datasets
Age=35 sex=M
par1=0.029
par2=2.926
Live curves. Stress=9
Datasets
Age=40 sex=M
par1=0.033
par2=2.256
Datasets
Age=45 sex=M
par1=0.062
par2=1.563
Datasets
Age=50 sex=M
par1=0.042
par2=2.175
Datasets
Age=5 sex=F
par1=0.01
par2=6.626
time, hours
time, hours
time, hours

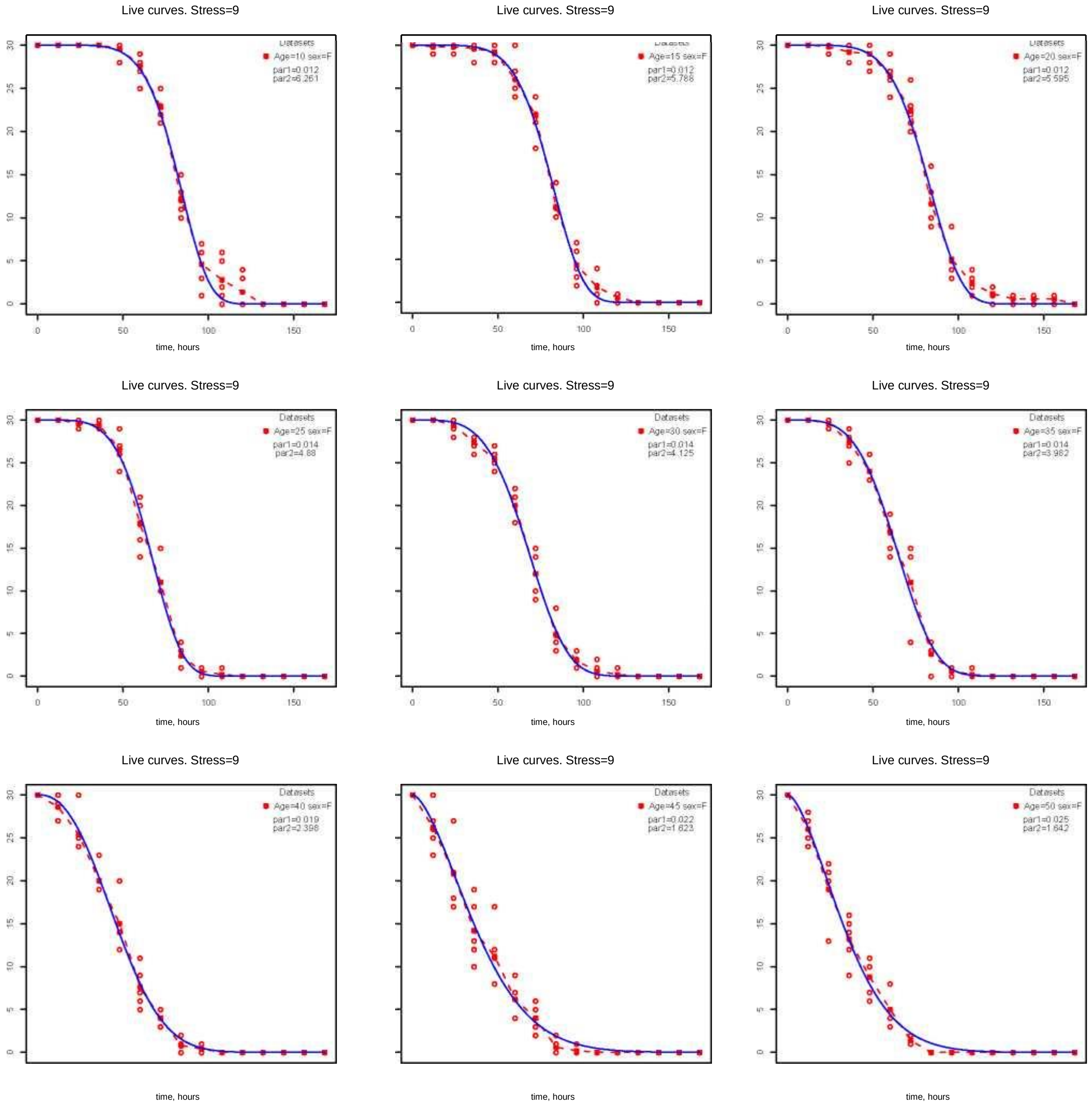
Live curves. Stress=9
Age=10 sex=F
par1=0.012
par2=6.261
time, hours
Live curves. Stress=9
Age=15 sex=F
par1=0.012
par2=5.788
time, hours
Live curves. Stress=9
Age=20 sex=F
par1=0.012
par2=5.595
time, hours
Live curves. Stress=9
Datasets
Age=25 sex=F
par1=0.014
par2=4.88
time, hours
Live curves. Stress=9
Datasets
Age=30 sex=F
par1=0.014
par2=4.125
time, hours
Live curves. Stress=9
Datasets
Age=35 sex=F
par1=0.014
par2=3.982
time, hours
Live curves. Stress=9
Datasets
Age=40 sex=F
par1=0.019
par2=2.398
time, hours
Live curves. Stress=9
Datasets
Age=45 sex=F
par1=0.022
par2=1.823
time, hours
Live curves. Stress=9
Datasets
Age=50 sex=F
par1=0.025
par2=1.642
time, hours

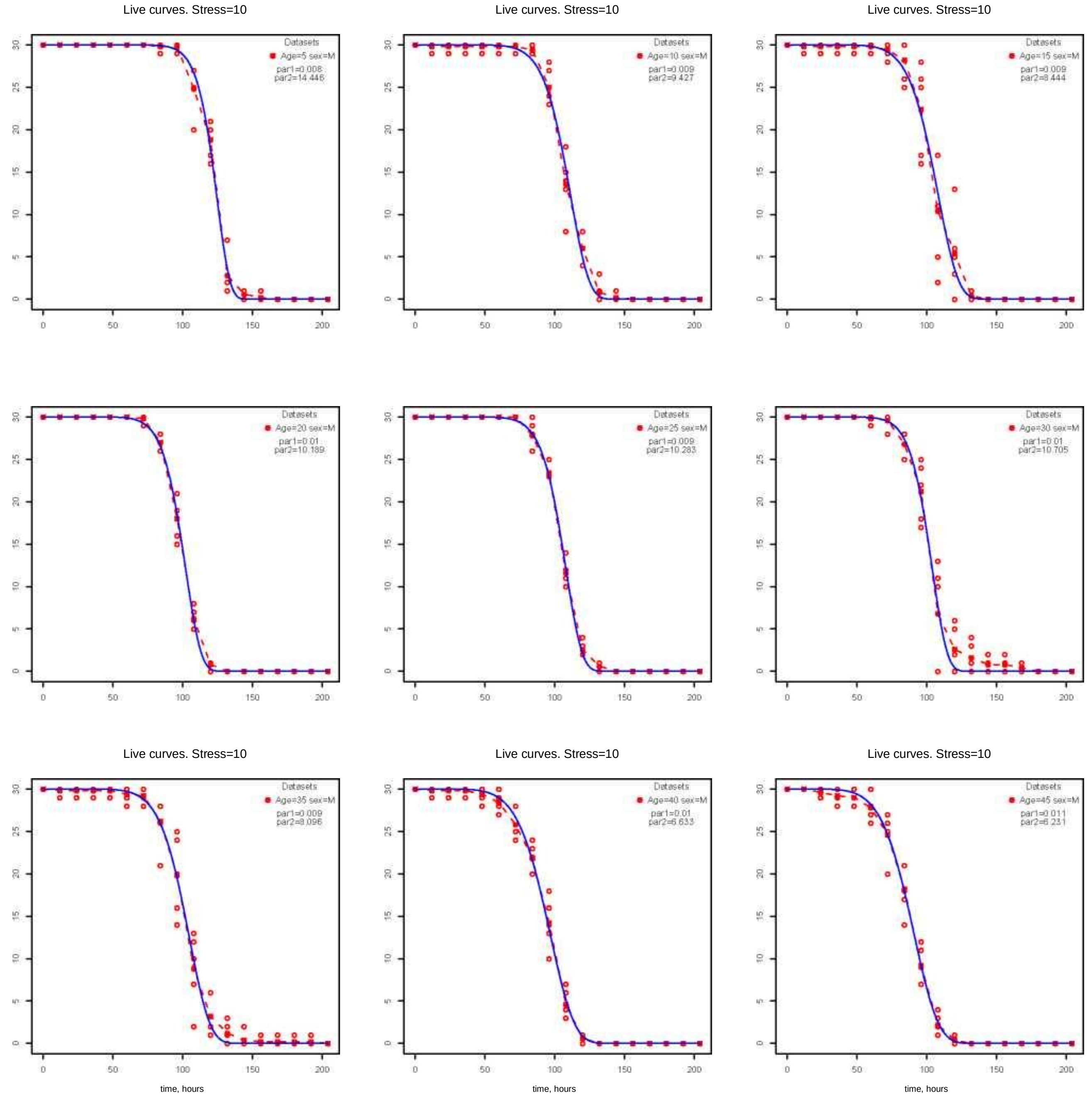

Live curves. Stress=10
Datasets
Age=5 sex=M
par1=0.008
par2=14.446
Live curves. Stress=10
Datasets
Age=10 sex=M
par1=0.009
par2=9.427
Live curves. Stress=10
Datasets
Age=15 sex=M
par1=0.009
par2=8.444
Datasets
Age=20 sex=M
par1=0.01
par2=10.189
Datasets
Age=25 sex=M
par1=0.009
par2=10.283
Datasets
Age=30 sex=M
par1=0.01
par2=10.705
Live curves. Stress=10
Datasets
Age=35 sex=M
par1=0.009
par2=8.096
time, hours
Live curves. Stress=10
Datasets
Age=40 sex=M
par1=0.01
par2=6.633
time, hours
Live curves. Stress=10
Datasets
Age=45 sex=M
par1=0.011
par2=6.231
time, hours

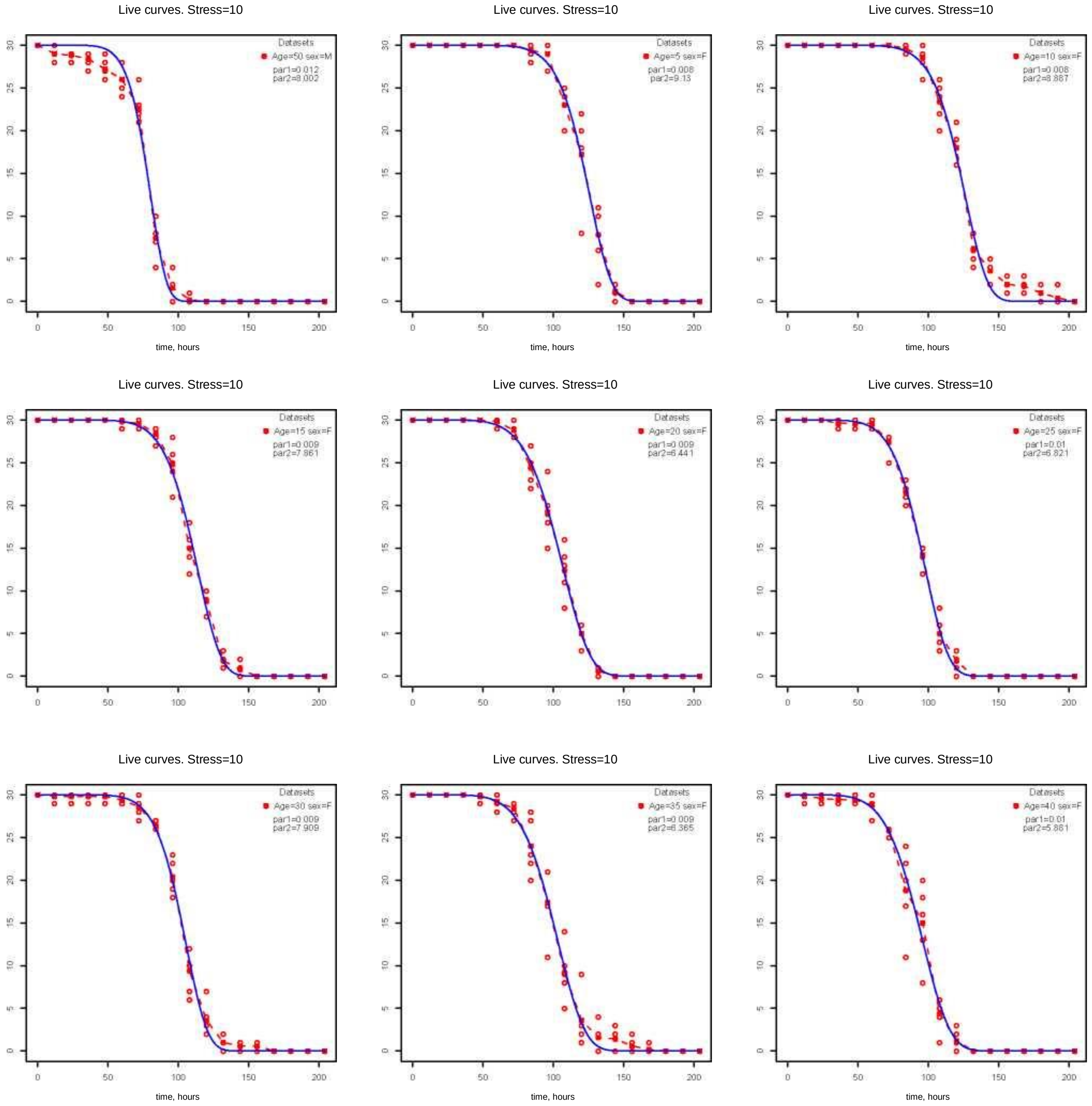

Live curves. Stress=10
Datasets
Age=50 sex=M
par1=0.012
par2=8.002
time, hours
Live curves. Stress=10
Datasets
Age=5 sex=F
par1=0.008
par2=9.13
time, hours
Live curves. Stress=10
Datasets
Age=10 sex=F
par1=0.008
par2=8.887
time, hours
Live curves. Stress=10
Datasets
Age=15 sex=F
par1=0.009
par2=7.861
Live curves. Stress=10
Datasets
Age=20 sex=F
par1=0.009
par2=6.441
Live curves. Stress=10
Datasets
Age=25 sex=F
par1=0.01
par2=6.821
Live curves. Stress=10
Datasets
Age=30 sex=F
par1=0.009
par2=7.909
time, hours
Live curves. Stress=10
Datasets
Age=35 sex=F
par1=0.009
par2=6.365
time, hours
Live curves. Stress=10
Datasets
Age=40 sex=F
par1=0.01
par2=5.881
time, hours

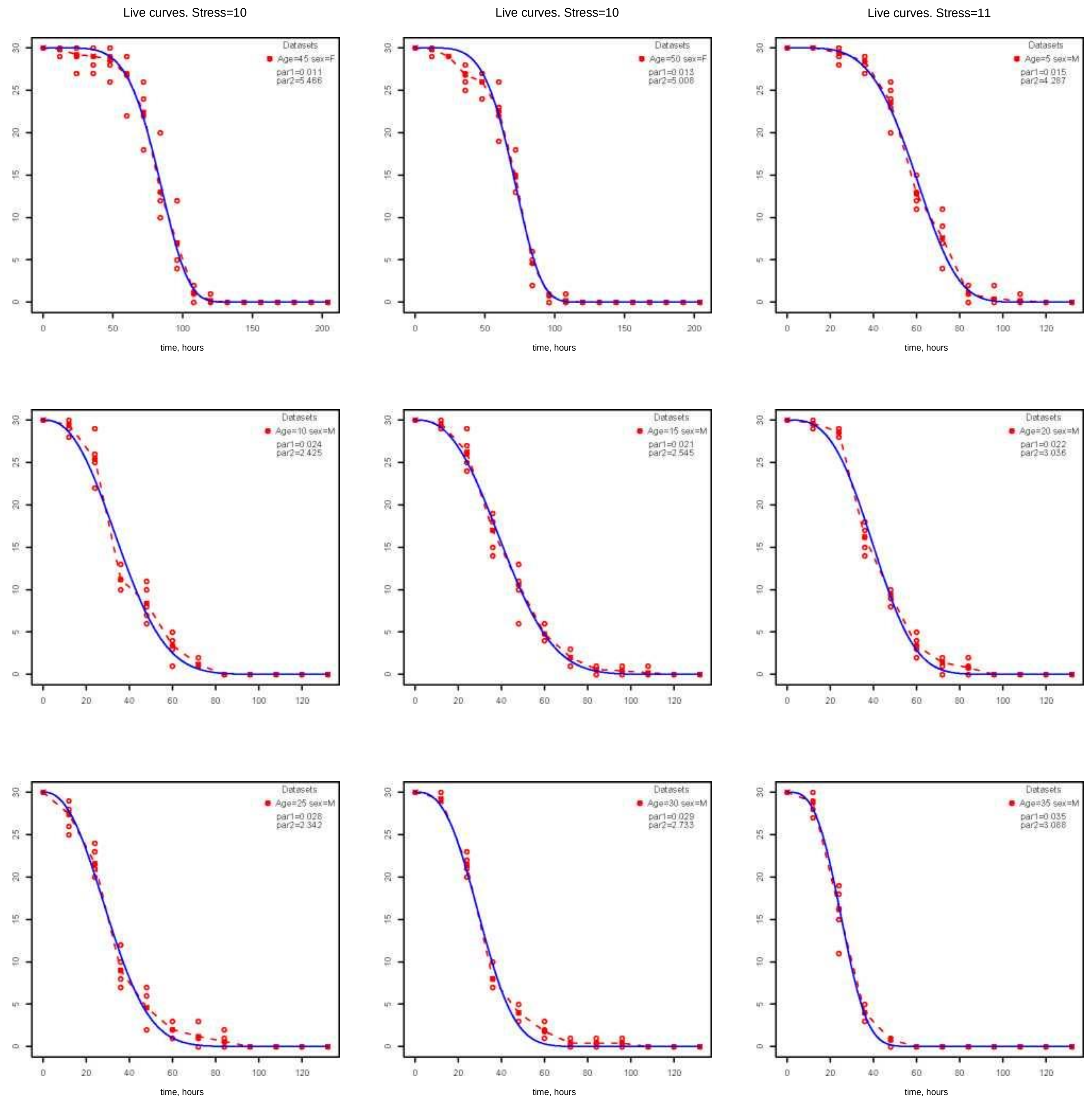
Live curves. Stress=10
Datasets
Age=45 sex=F
par1=0.011
par2=5.466
time, hours
Live curves. Stress=10
Datasets
Age=50 sex=F
par1=0.013
par2=5.008
time, hours
Live curves. Stress=11
Datasets
Age=5 sex=M
par1=0.015
par2=4.287
time, hours
Datasets
Age=10 sex=M
par1=0.024
par2=2.425
Datasets
Age=15 sex=M
par1=0.021
par2=2.545
Datasets
Age=20 sex=M
par1=0.022
par2=3.036
Datasets
Age=25 sex=M
par1=0.028
par2=2.342
time, hours
Datasets
Age=30 sex=M
par1=0.029
par2=2.733
time, hours
Datasets
Age=35 sex=M
par1=0.035
par2=3.088
time, hours

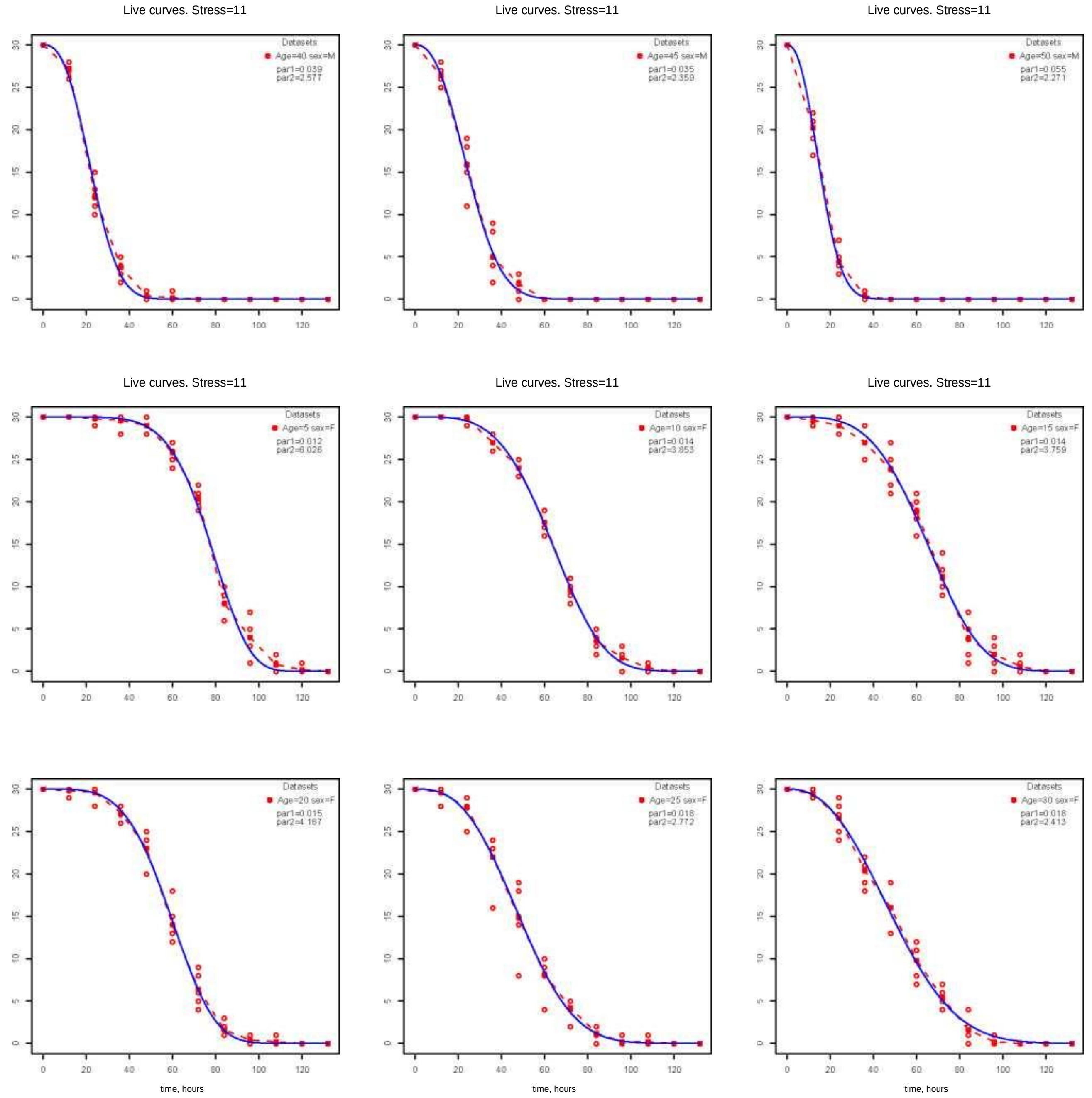
Live curves. Stress=11
Datasets
Age=40 sex=M
par1=0.039
par2=2.577
Live curves. Stress=11
Datasets
Age=45 sex=M
par1=0.035
par2=2.359
Live curves. Stress=11
Datasets
Age=50 sex=M
par1=0.055
par2=2.271
Live curves. Stress=11
Datasets
Age=5 sex=F
par1=0.012
par2=6.026
Live curves. Stress=11
Datasets
Age=10 sex=F
par1=0.014
par2=3.853
Live curves. Stress=11
Datasets
Age=15 sex=F
par1=0.014
par2=3.759
Datasets
Age=20 sex=F
par1=0.015
par2=4.167
time, hours
Datasets
Age=25 sex=F
par1=0.018
par2=2.772
time, hours
Datasets
Age=30 sex=F
par1=0.018
par2=2.413
time, hours

Live curves. Stress=11

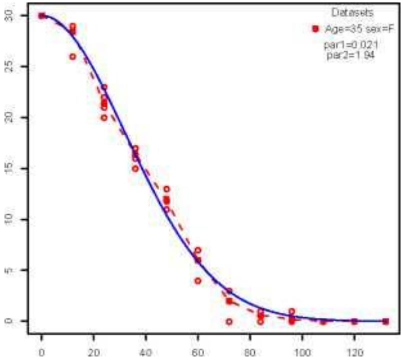

Live curves. Stress=11

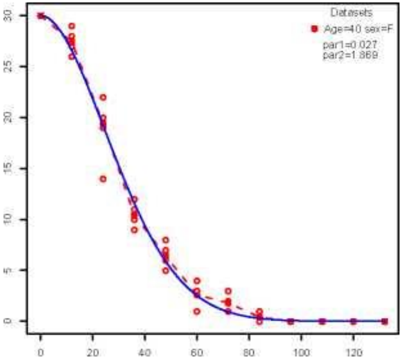

Live curves. Stress=11

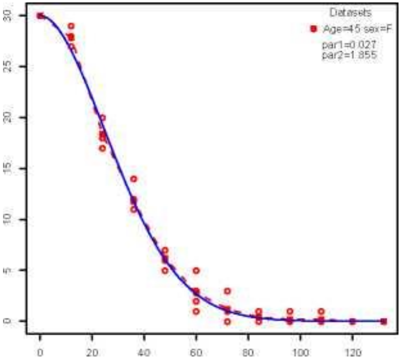

Live curves. Stress=11

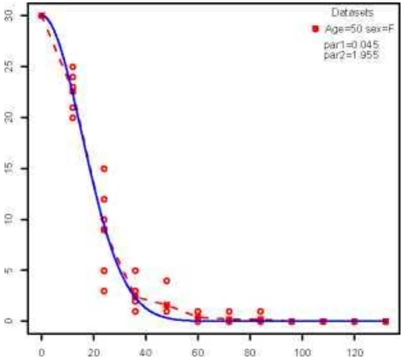

Live curves. Stress=12

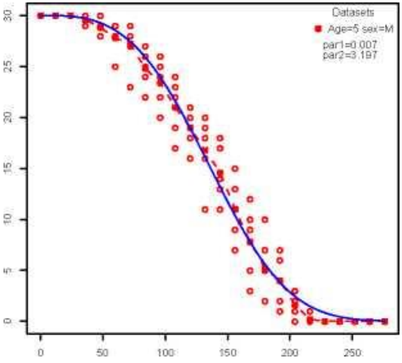

Live curves. Stress=12

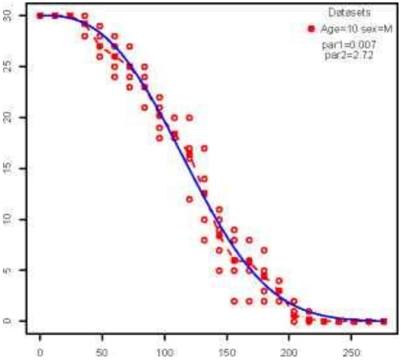

Live curves. Stress=12

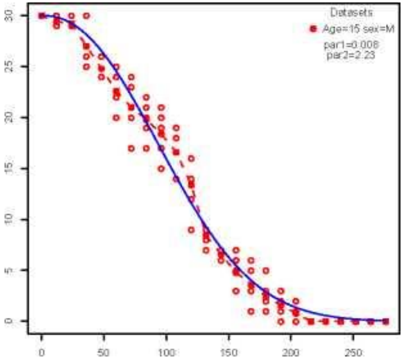

time, hours

Live curves. Stress=12

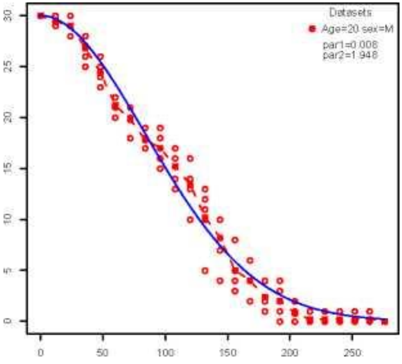

time, hours

Live curves. Stress=12

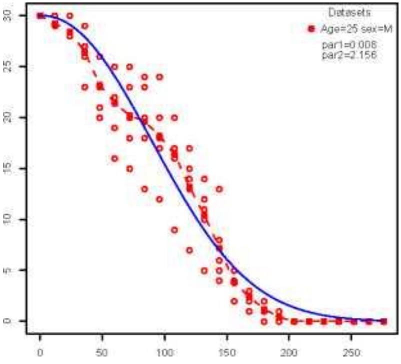

time, hours

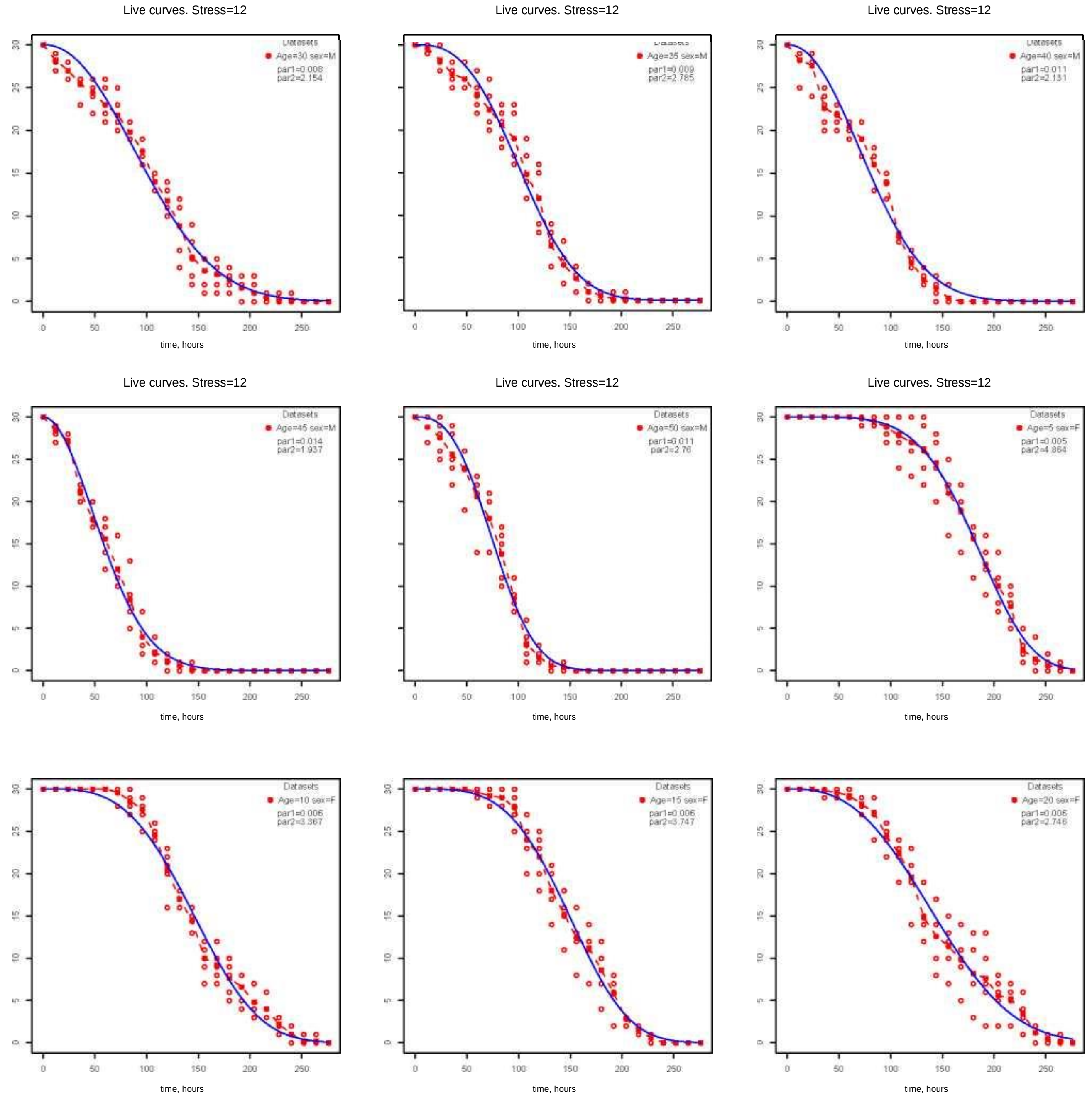
Live curves. Stress=12
Datasets
Age=30 sex=M
par1=0.008
par2=2.154
time, hours
Live curves. Stress=12
Datasets
Age=35 sex=M
par1=0.009
par2=2.785
time, hours
Live curves. Stress=12
Datasets
Age=40 sex=M
par1=0.011
par2=2.131
time, hours
Live curves. Stress=12
Datasets
Age=45 sex=M
par1=0.014
par2=1.937
time, hours
Live curves. Stress=12
Datasets
Age=50 sex=M
par1=0.011
par2=2.76
time, hours
Live curves. Stress=12
Datasets
Age=5 sex=F
par1=0.005
par2=4.864
time, hours
Datasets
Age=10 sex=F
par1=0.006
par2=3.367
time, hours
Datasets
Age=15 sex=F
par1=0.006
par2=3.747
time, hours
Datasets
Age=20 sex=F
par1=0.006
par2=2.746
time, hours

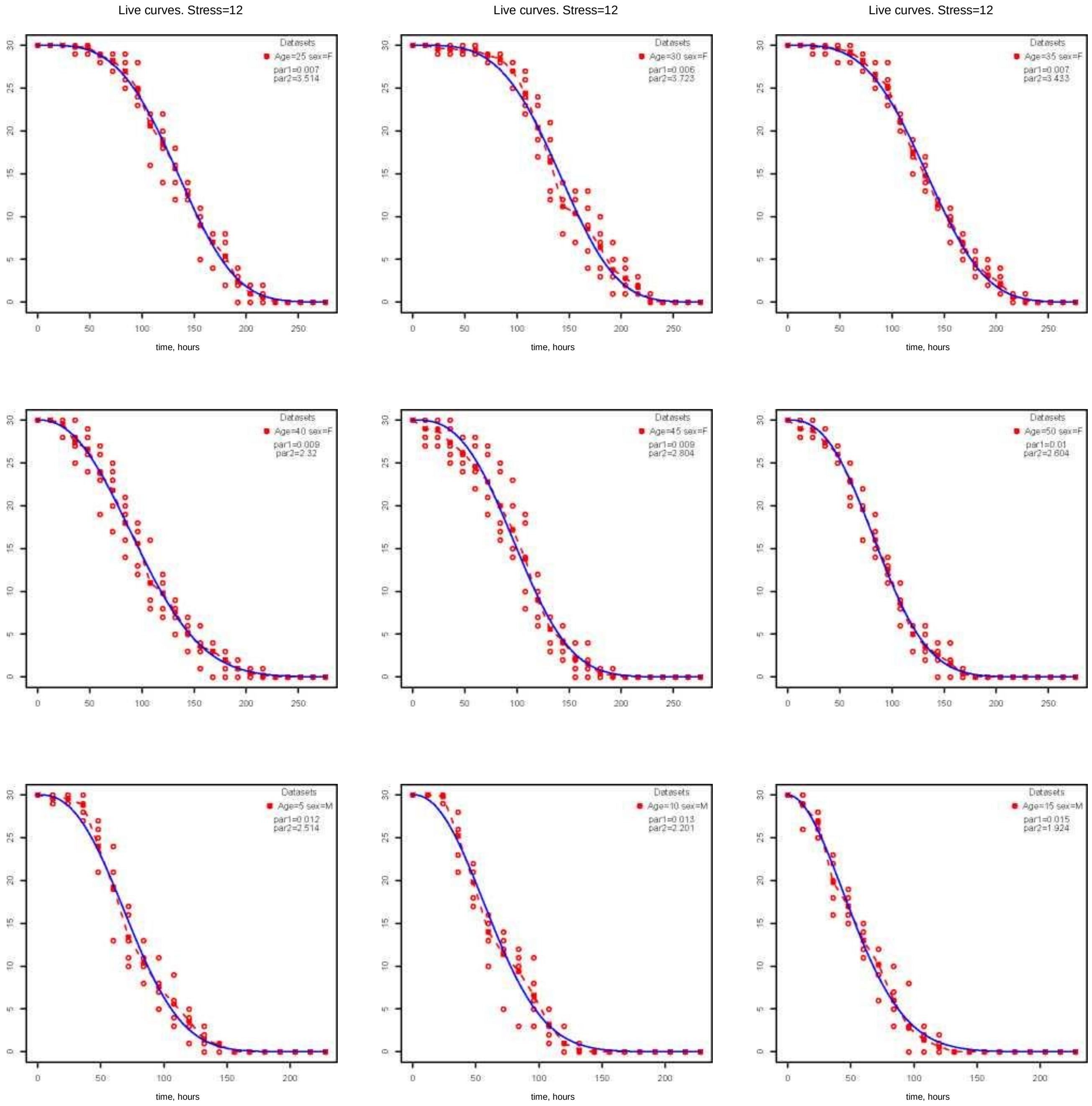
Live curves. Stress=12
Live curves. Stress=12
Live curves. Stress=12
Datasets
Age=25 sex=F
par1=0.007
par2=3.514
Datasets
Age=30 sex=F
par1=0.006
par2=3.723
Datasets
Age=35 sex=F
par1=0.007
par2=3.433
Datasets
Age=40 sex=F
par1=0.009
par2=2.32
Datasets
Age=45 sex=F
par1=0.009
par2=2.804
Datasets
Age=50 sex=F
par1=0.01
par2=2.604
Datasets
Age=5 sex=M
par1=0.012
par2=2.514
Datasets
Age=10 sex=M
par1=0.013
par2=2.201
Datasets
Age=15 sex=M
par1=0.015
par2=1.924
time, hours

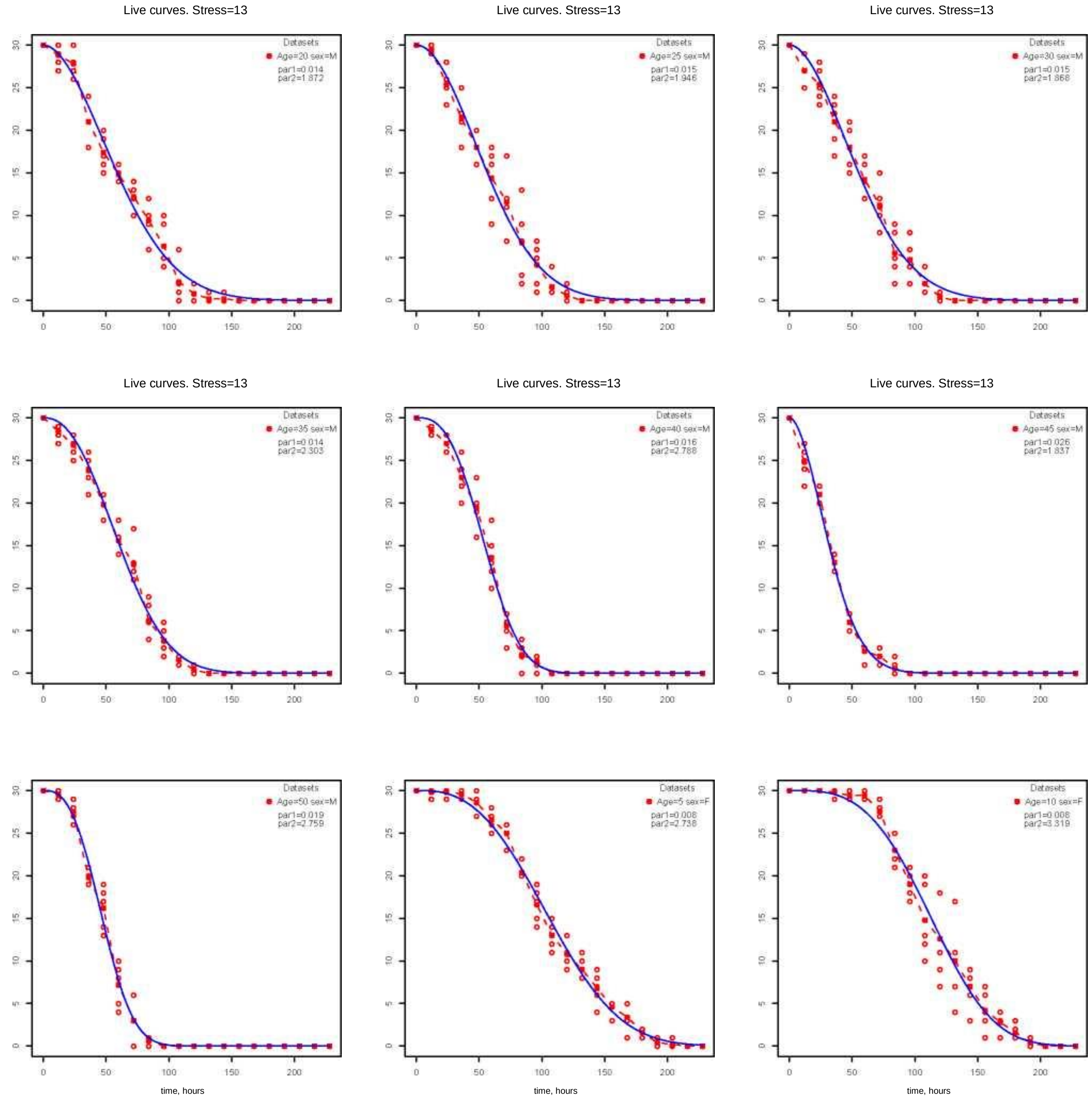
Live curves. Stress=13
Datasets
Age=20 sex=M
par1=0.014
par2=1.872
Live curves. Stress=13
Datasets
Age=25 sex=M
par1=0.015
par2=1.946
Live curves. Stress=13
Datasets
Age=30 sex=M
par1=0.015
par2=1.868
Live curves. Stress=13
Datasets
Age=35 sex=M
par1=0.014
par2=2.303
Live curves. Stress=13
Datasets
Age=40 sex=M
par1=0.016
par2=2.788
Live curves. Stress=13
Datasets
Age=45 sex=M
par1=0.026
par2=1.837
Datasets
Age=50 sex=M
par1=0.019
par2=2.759
time, hours
Datasets
Age=5 sex=F
par1=0.008
par2=2.738
time, hours
Datasets
Age=10 sex=F
par1=0.008
par2=3.319
time, hours

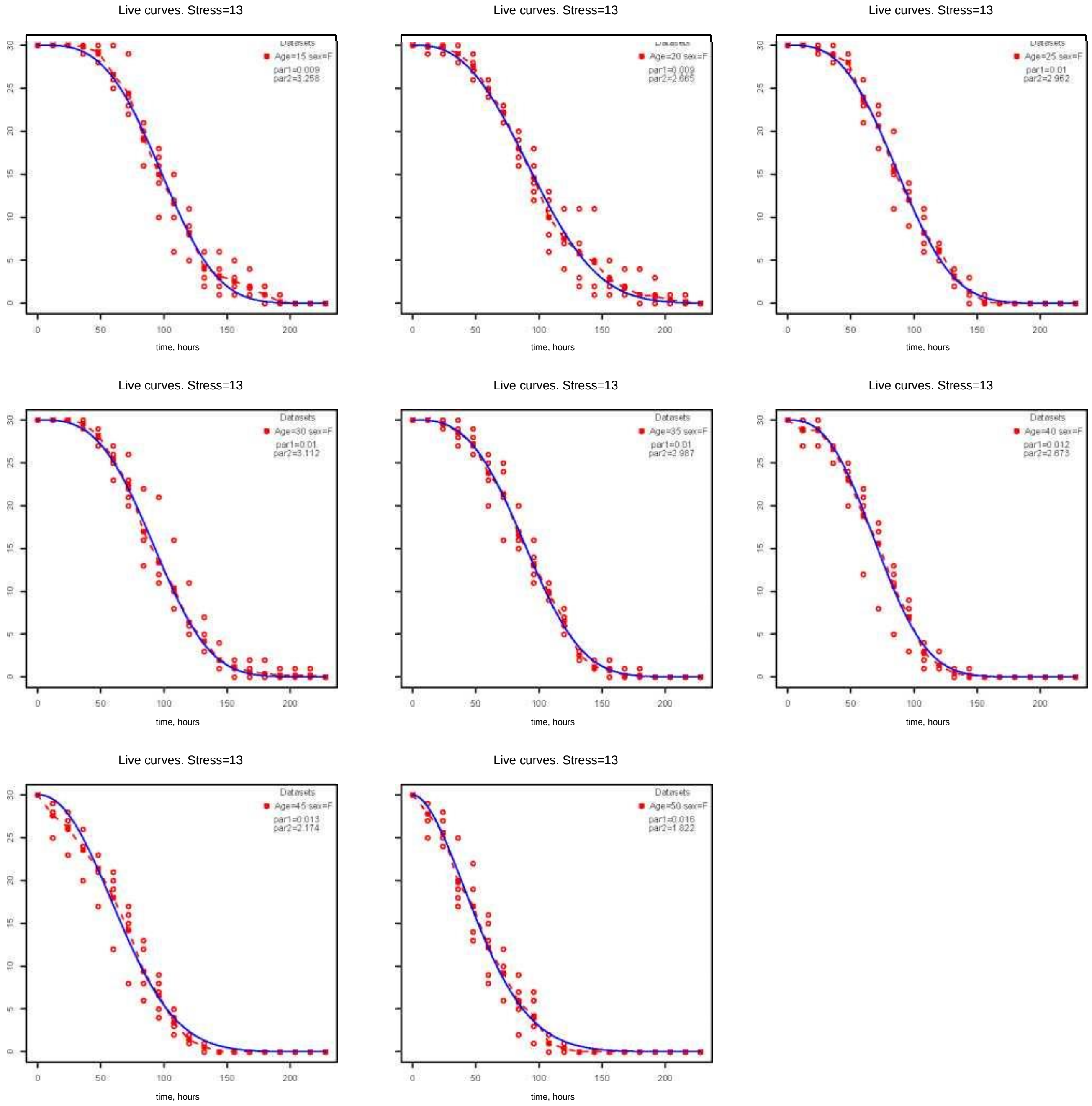
Live curves. Stress=13
Datasets
Age=15 sex=F
par1=0.009
par2=3.258
time, hours
Live curves. Stress=13
Datasets
Age=20 sex=F
par1=0.009
par2=2.665
time, hours
Live curves. Stress=13
Datasets
Age=25 sex=F
par1=0.01
par2=2.962
time, hours
Live curves. Stress=13
Datasets
Age=30 sex=F
par1=0.01
par2=3.112
time, hours
Live curves. Stress=13
Datasets
Age=35 sex=F
par1=0.01
par2=2.987
time, hours
Live curves. Stress=13
Datasets
Age=40 sex=F
par1=0.012
par2=2.673
time, hours
Live curves. Stress=13
Datasets
Age=45 sex=F
par1=0.013
par2=2.174
time, hours
Live curves. Stress=13
Datasets
Age=50 sex=F
par1=0.016
par2=1.822
time, hours
