# Supplementary material for: The Resistance of Drosophila melanogaster to Oxidative, Genotoxic, Proteotoxic, Osmotic Stress, Infection, and Starvation Depends on Age According to the Stress Factor
Source: Antioxidants (Basel). 2020 Dec 7;9(12):1239. doi: 10.3390/antiox9121239 (PMC7762242; doi:10.3390/antiox9121239)
Supplement: Supplementary file 1 [file antioxidants-09-01239-s001.zip › Online Resource 2.pdf]

The resistance of *Drosophila melanogaster* to oxidative, genotoxic, proteotoxic, osmotic stress, infection and starvation depends on age according to the stress factor

Alexei A. Belyi, Alexey A. Alekseev, Alexander Y. Fedintsev, Stepan N. Balybin, Ekaterina N. Proshkina, Mikhail V. Shaposhnikov and Alexey A. Moskalev

Online Resource 2

Description of the approach of empirical modeling of survival curves, as well as the theoretical method of predicting a priori an unknown age group of individuals, according to the results of stress experiments.

Preprocessing of raw data. Obtained in the experiment raw data sets for each stress factor and sex (13 stressors, 2 sex, total 26 datasets containing experimental data for different ages 10 - 5, 10, 15, ..., 50 days), were converted into data sets, reflecting the number of live specimens in each time of experiment according to the formula:

$$N_{live}(T) = N_{full} - \sum_{t=0}^{t=T} N_{death}(t)$$

where N_{live} - the number of live specimens N_{full} = 30 - the total number of specimens taken for one series of experiments, T - the time at which we calculate the number of live animals.

We call variant - the results (i.e., the number of live specimens - N_{live}) conducted five experiments to certain stress factors (identifier of stress reported in Table A1.), Sex and age specimens. We call series - the results of every single experiment. That is, each variant in our definition consists of 5 series. We call cases - the set of variants obtained for ten ages (5 to 50 days), which corresponds to a particular sex and stress. The sex in the calculations indicated as 1 (or M) - male and 2 (or F) - female. Thus, we received the raw data that we used to construct an empirical model.

Table A1. Identifiers of used stress.

The identifier of stress	Stress
1	Paraquat (20 mM)
2	Starvation
3	Hyperthermia (35 °C)
4	Infection by *Beauveria Bassiana*

5	NaCl (400 mM)
6	$ZnCl_2$ (5 mM)
7	$ZnCl_2$ (10 mM)
8	$CuSO_4$ (10 mM)
9	$CuSO_4$ (15 mM)
10	$CdCl_2$ (1 mM)
11	$CdCl_2$ (5 mM)
12	$FeCl_3$ (10 mM)
13	$FeCl_3$ (15 mM)

The calculation mean values. For each variant, we calculate the arithmetic mean N_{live} values at each time point of the experiment. That is, we averaged 5 series of each variant:

$$\overline{N_{live}(t = t, i, j, k)} = \frac{1}{5}\sum_{m=1}^{5} N_{live}(t = t, i, j, k, m)$$

where $i = \overline{1{,}13}$ - the index of stress, $j = \overline{1{,}2}$ - sex index, $k = \overline{1{,}10}$ - age index, $m = \overline{1{,}5}$ - number of series. Although, in the future, when fitting the data, we used the entire set of points for each variant the average values allowed visualizing data in comparison with the model.

Data fitting. The function for the extinction curve we have chosen - $f(t) = e^{-(\lambda * t)^{\nu}}$, at t = 0 is set to 1, and with these restrictions on the parameters, asymptotically approaches zero as $t \to \infty$. This implies the need to select the normalization of the function, equal to the $N_{full} = 30$:

$$\widehat{N_{live}} = N_{full} * f(t)$$

where $\widehat{N_{live}}$ - model value of survivors number at time t in a certain variant.

For each variant, we made the fitting of the model to experimental data. To obtain parameter was used function “optim” of standard “stats” package in the environment of language R. The methods of optimization used in this function is proposed in the article (Nelder and Mead 1965). It use to optimize only the values of the function itself. All the curves obtained, in conjunction with the corresponding experimental points are shown in Online Resource 1. Figures in Online Resource 1 show the fitting of the original data. Red line shows the original reference points, the red bar line connects mean values, the blue dotted line - the model curve. In the program, and the graphs parameters ν and λ of the model are indicated as p1 and p2 respectively. As can be seen in the diagrams, the theoretical curves for the found parameters, quite well describe the survival curves, and are near the mean values for the different series.

Next, we constructed the dependencies of each of the obtained parameters on age (Fig. A1).

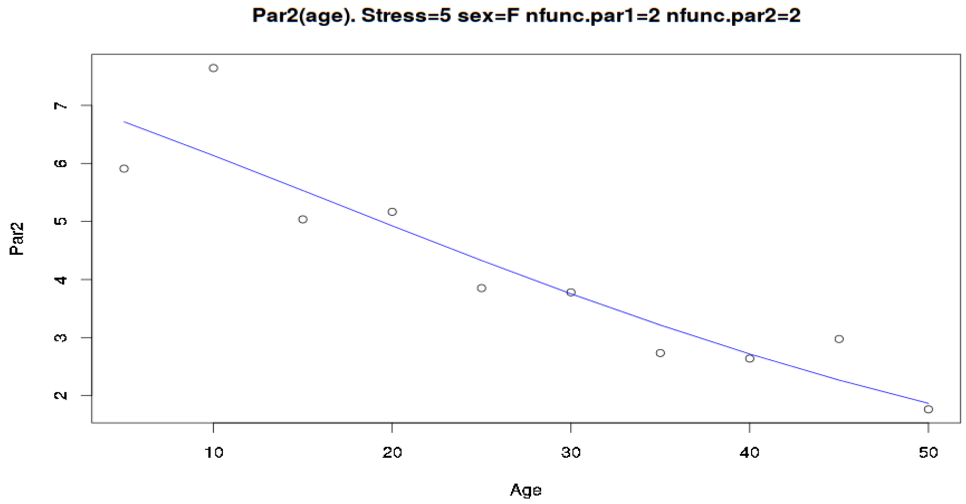

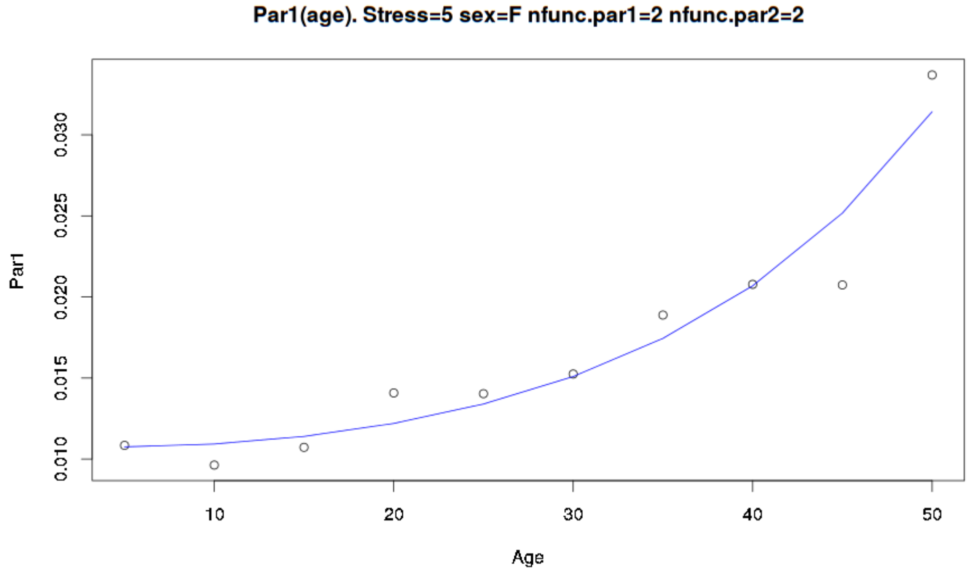

Fig. A1 Dependences of the obtained parameters par1 and par2 on age for variant 5F.Points - the obtained values of the parameters, curves - empirical curves

As can be seen in the Fig. A1, the obtained dependences of the model parameters on the age of the individuals are complex and nonmonotonic, which makes it difficult to directly construct explicit models for the dependence of the parameters of the model chosen by us from the age. We attempted to describe these dependencies explicit functions, however, the model error is quite significant, which sharply reduced the predictive power of the model, that is, the accuracy of estimating the unknown age from a pair of parameters (par1, par2), obtained as a result of fitting data for this age.

We carried out the bootstrapping procedure in order to clarify the stability of the determination of model parameters during fittings and to determine the areas in which the data of different variants are well separated (with the same stress and field, but of different ages).

Bootstrapping of following algorithm was used:

1) Select one variant (5 series, the same stress, sex, age).

2) Choose a time, which correspond to 5 different series points.

3) Make a random sample with 5 possible values iterations of these 5 points.

4) Repeat step 3 for all the moments of time of this variant. We get the "simulated" variant, which number of dots coincides with the number of points in the original variant.

5) Fitting the resulting variant of our model. We get a couple of parameters (par1, par2).

6) Repeat step 2-5 for 50 times. We get the 50 pairs of parameter values (par1, par2).

7) Repeat steps 1-6 for the other variants.

Thus, instead of one pair of the parameters for each variant (one point on the plane par1-par2) we obtain a "cloud" of 50 points (Online Resource 3). Figures in Online Resource 3 show the results of the original data bootstrapping on the plane (par1, par2). par1 and par2 - model parameters obtained by fitting the data for a certain age. The dots indicate the results of the original data bootstrapping for different ages (Age = 5, 10, ... 50 days) when exposed to stress (stress factors transcript notation is provided in Table A1). M - male, A - female.

The configurations of these "clouds" make it possible to conclude in which areas the model well, and in which - bad separates the various variants (ie, data sets for different ages). In this regard, we wondered how for the variant of a priori unknown age, we could estimate age and determine the accuracy of that assessment. Obviously, the first step should be to make data bootstrapping for investigated unknown variant, and get a "cloud" of points on the plane (par1, par2) for it. Correlation of the shape and location of the "cloud" in reference to the "cloud" for variants with known ages, allow us, as will be shown below, to make the required assessment of the age for unknown variant.

Obtaining data for intermediate ages ("virtual" data). To assess the accuracy of the predictions for each stress-sex at all examined ages (i.e., for a particular case) the method of multiple generation virtual data was used based on the distribution parameters of adjacent ages. Intermediate ages (a_v) is the arithmetic mean:

$$a_v(i) = 2.5 + 5 * i; i \in (1; 9)$$

The algorithm is the following:

1. For the moment of time t_i from the time grid of given stress-sex select the point corresponding to the two adjacent age, $a_{left} = a_v - 2.5$ and $a_{right} = a_v + 2.5$, that is, for example, for a virtual age $a_v = 7.5$ we take two sets of points $a_{left} = 5$ and $a_{right} = 10$ for the moment of time t_i.

2. Separate for the left and right cases calculate the arithmetic mean (M) and the dispersion (D) of a set of 5 points distribution. Obtain a value of M (a_{left} = a_{left}, t = t_i), D (a_{left} = a_{left}, t = t_i), M (a_{right} = a_{right}, t = t_i), D (a_{right} = a_{right}, t = t_i).

3. As a mean and dispersion values for a_v and t_i takes the value of the geometric means from the corresponding left and right values:

$$M_{virt}(a_v, t_i) = \sqrt{M(a_{left} = a_{left}, t = t_i) \cdot M(a_{right} = a_{right}, t = t_i)} \quad (1)$$

$$D_{virt}(a_v, t_i) = \sqrt{D(a_{left} = a_{left}, t = t_i) \cdot D(a_{right} = a_{right}, t = t_i)} \quad (2)$$

4. Next, we use obtained by formulas (1) and (2) the values as parameters of normal distribution for obtaining random points.

5. Repeat steps 1-4 for all the moments of time t_i from the time grid of the given stress-sex.

To determine the distribution parameters for generating virtual data, in addition to the geometric mean, we tried to take the arithmetic mean of the adjacent variants distributions parameters. In this case, the obtained point cloud for the virtual data do not located between clouds of actual data.

Virtual data for the case of infection by a fungus *B. bassiana* of males and females are shown in Fig. A2.

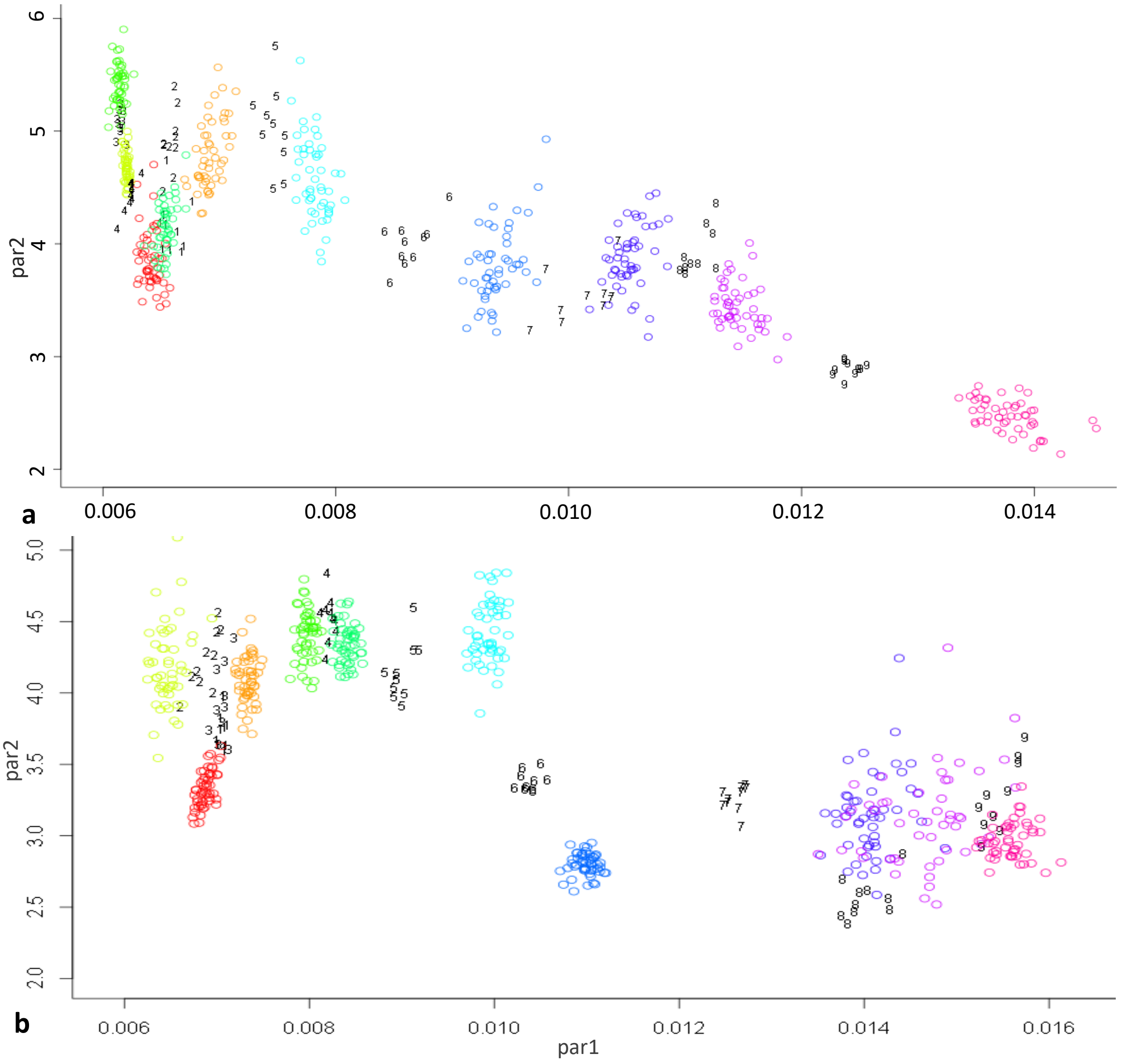

Fig. A2 Virtual data for the case of *B. bassiana* infection of male (a) and female (b) on the plane (par1, par2). par1 and par2 - model parameters obtained by fitting the data for a certain age. Colored dots indicate the results of the original data bootstrapping, the black symbols - bootstrapping the virtual data (for adjacent age)

Age estimation algorithm for survival data of unknown age flies:

1) Perform bootstrapping of unknown variants and receive 50 pairs of values for the parameters (par1, par2).

2) Circumscribe the cloud of points by an ellipse. That is, to find such an ellipse, which contains the maximum number of points of the cloud, but limited by the size of the cloud. In other words, the objective is to obtain five ellipse parameters - coordinates of the center, the value of the major and minor semi-axes, and rotation angle of the ellipse relative to the x-axis.

3) Circumscribe by the ellipses each "cloud" obtained by bootstrapping pairs (par1, par2) for variants with known ages.

4) Receive the parameters of so-called intermediate ellipses for each variant and for each combination of two adjacent age variants. In our calculations, we took the number of ellipses, equal to 10, for each of these combinations. All parameters of these ellipses are obtained by linear interpolation of the corresponding parameters of adjacent ellipses.

That is, each of the 5 parameters of the ellipse is defined as the point on the segment of a linear function, and at the ends of this interval the value of function equals to the values of this parameter for adjacent variants (Fig. A3). Thus, for each variant, and in all range of ages from 5 to 50 days we get a set of 10 * 9 = 90 ellipses, each of which is specified by quintet parameters. To each of these ellipses, we compared some intermediate age, which varies linearly in a series of ellipses. Moreover, among these ellipses, there are those that correspond to the original data.

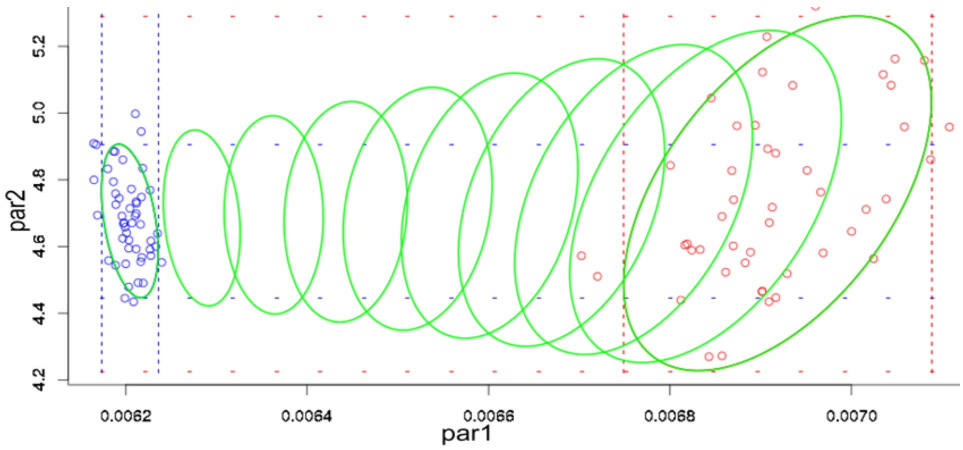

Fig. A3 Obtaining of the intermediate ellipses in the field model parameters. par1 and par2 - model parameters obtained by fitting the data. The dots indicate the results of bootstrapping of the original data for the age of 10 days (blue dots) and 15 days (red dots) under infection of female by fungus B. Bassiana

5) For the variant with the same stress and sex, as unknown variant, go through the obtained 90 ellipses and for each ellipse determine the area of intersection with the ellipse obtained by bootstrapping points of unknown variant (step 2). Thus, we obtain the dependence of the area of the intersection from the "age" of corresponding ellipses.

6) We approximate the resulting dependence of the intersection area of the ellipses from the "age" using Gaussian function. As an estimate for the age unknown variant (aestim), we took the average value of the distribution. With the dispersion of the Gaussian function, we evaluate the prediction accuracy index.

Next, we take a closer look at some aspects of this algorithm.

The fitting of each cloud by an ellipse points. Consider the solution of the problem of circumscribing by an ellipse of some two-dimensional set of points in the plane. That is, the determination of the ellipse, which contains the maximum number of points of the cloud, but limited by size of the cloud. In other words, the objective is to obtain five ellipse parameters - coordinates of the center, the value of the major and minor semi-axes, and rotation angle of the ellipse relative to the x-

axis. To our delight, previously, the problem has been solved (Nesterov 2013). We summarize the main ideas of that article, underlying proposed there algorithm, which we have used in this work.

1. We write the equation of some ellipse in matrix form (in two-dimensional homogeneous coordinates):

$$(x, y, 1)\begin{pmatrix} a & b & c \\ d & e & f \\ g & h & i \end{pmatrix}(x, y, 1)^T = 0 \qquad (3)$$

Performing matrix multiplication (3) and determine an ellipse:

$$ax^2 + (d + b)xy + ey^2 + (g + c)x + (h + f)y + i = 0 \qquad (4)$$

Compare the formula (4) with the Mahalanobis squared-distance d (x, μ) from the random vector x to the multiplicity with a mean value μ and covariance matrix Σ:

$$d(\vec{x}, \vec{\mu}) = \sqrt{(\vec{x} - \vec{\mu})^T \Sigma^{-1} (\vec{x} - \vec{\mu})} \qquad (5)$$

$$d^2(\vec{x}, \vec{\mu}) = (\vec{x} - \vec{\mu})^T \Sigma^{-1} (\vec{x} - \vec{\mu}) \qquad (6)$$

As you can see, the representation of a Mahalanobis squared-distance (6) is identical to the record of the ellipse equation in matrix form (3). Thus, we have seen that the Mahalanobis distance describes an ellipse in the Euclidean space. Mahalanobis distance - it is simply the distance between the target point and the center of mass, divided by the width of the ellipse in the direction of a given point.

Next, remember that the chi-square distribution - the distribution of the sum of squares of k independent standard normal random variables (this distribution is parameterized by the number of degrees of freedom k). Note that this corresponds exactly to the Mahalanobis squared-distance (6). Thus, the probability that $\vec{x}$ lies within the ellipse is expressed by the following formula:

$$(\vec{x} - \vec{\mu})^T \Sigma^{-1} (\vec{x} - \vec{\mu}) \sim \chi^2(k) \qquad (7)$$

Now we can determine the ellipse parameters based on chi-square distribution - using quantile of this distribution.

The idea of obtaining the contour of the ellipse is as follows: take a series of points on the unit circle, move the circle in the center of mass of the dataset, then scale and stretch this circle in the right direction. Consider the geometric interpretation of the multivariate Gaussian distribution. It is an ellipse the major axis direction of which are the set of the covariance matrix eigenvalues and the relative length of the principal axes defined by the square roots of the corresponding eigenvalues.

We present an algorithm:

1. Choose a quantile values q. In our calculations, we have taken the value of q = 0.8.
2. We calculate the value of the quantile; the degree of freedom is equal to 2.
3. Scale the found earlier covariance matrix for a given set of points to the value of the quantile.
4. Calculate the eigenvalues of the obtained covariance matrix.

5. Scale the unit circle so the length the semi-axes become equal to the square roots of the eigenvalues.

6. Turn the resulting ellipse at an angle determined by the eigenvalues.

7. Shift the center of the rotated ellipse in the center of the original distribution.

Thus, for each variant of the model parameters distribution (par1, par2), obtained during bootstrapping, we can define the parameters of the ellipse that covers this set of points (Fig. A4).

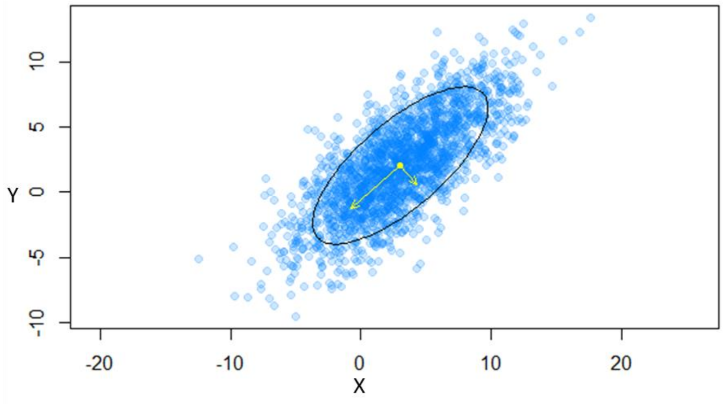

Fig. A4 The contours of ellipses for different q. Drawing from the article (Nesterov 2013). Ellipse shows the results of finding the contours at different q in the range of 0.1 to 0.95. Yellow arrows shows the eigenvectors of the covariance matrix, scaled to the square roots of the corresponding eigenvalues. They determine the direction of the main axes of the ellipses

The algorithm for finding the area of the intersection of ellipses. The algorithm is based on a Monte Carlo approach. For an ellipse defined by five parameters vertical and horizontal tangents are initially found that define a rectangle in which the ellipse is inscribed. Further, according to the Monte Carlo method we randomly "cast" point in this rectangle, and determines whether each of these points in both the first and second compared ellipses. The area of intersection of the ellipses defined by formula:

$$S_{intersect} = S_{rect} \frac{N_i}{N_{all}} \quad (8)$$

where $S_{intersect}$ - the area of intersection of two ellipses, N_i - the number of points that lie inside the first and second ellipses, N_{all} - the total number of "casted" points.

The criterion of finding the point inside the ellipse $(x/a)^2 + (y/b)^2 \leqslant 1$ where the coordinates x_1 and y_1 are calculated using trivial transformations:

$$x_1 = ((x - x_0) \cdot cos(\phi) + (y - y_0) \cdot sin(\phi))$$
$$y_1 = (-(x - x_0) \cdot sin(\phi) + (y - y_0) \cdot cos(\phi)) \quad (9)$$

where (x, y) - coordinates of the determined point in the original coordinate system, (x1, y1) - the coordinates of a vector in the shifted (x0, y0), and rotated at an angle φ coordinate system. x0, y0 - the coordinates of the center of the ellipse, the angle φ - angle of rotation of the ellipse relative to the axis OX. The diagram of intersection ellipse is shown in Fig. A5.

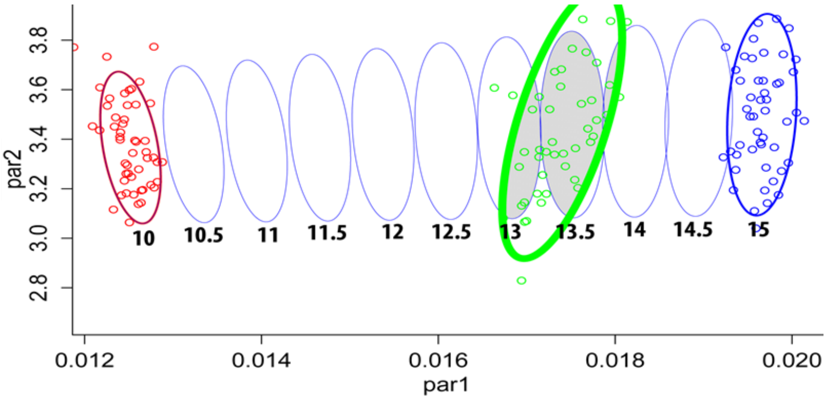

Fig. A5 Determination of the intersection area of the estimated ellipses and ellipse constructed with the virtual data for the case of paraquat exposure on females on the plane (par1, par2). par1 and par2 - model parameters obtained by fitting the data for a certain age. The red and blue dots indicate the initial data bootstrapping results for age 10 and 15 days respectively, the green dots - bootstrapping virtual data (for "intermediate" age)

The next step was the calculation of the ellipses intersection area and obtaining an average of the dependence of the ellipse intersection area, built on cloud-based virtual data bootstrapping, from the estimated age a_{estim}.

According to the selected algorithm is possible to define the area of intersection of the ellipse for the point cloud obtained by bootstrapping of the original data with the ellipse obtained in the same manner but for the data set of unknown age.

Method for determining the predictions accuracy

1. Generates 100 sets of virtual data for each stress, gender and 9 time points (from 7.5 to 47.5 in steps of 1). The total number of sets is 100 * 2 * 13 * 9 = 23400

2. For each set, a bootstrapping procedure is performed to obtain the corresponding "point cloud" on the plane of the two parameters par1 and par2. We describe the given cloud of points by an ellipse, and we obtain 5 parameters that characterize the ellipse.

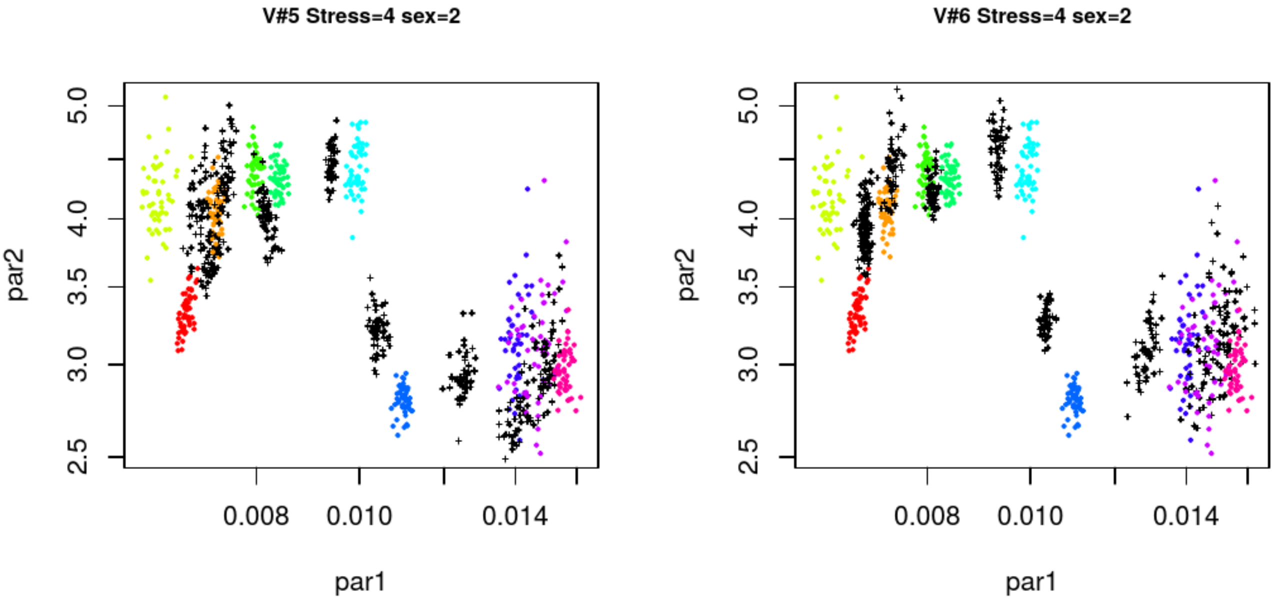

Fig. A6 Bootstrapping for virtual interim data. Even virtual crosses are marked with virtual data, color circles are the results of basecreping the original data. The example shows the variants 5 and 6 for stress 2 and sex 2 (females)

3. With the previously described "sliding ellipse" method, we determine the intersection of the resulting ellipse with ellipses constructed from bootstrapping data for the initial data set (only 10 ellipses for ages from 5 to 50 days for each stress and sex).

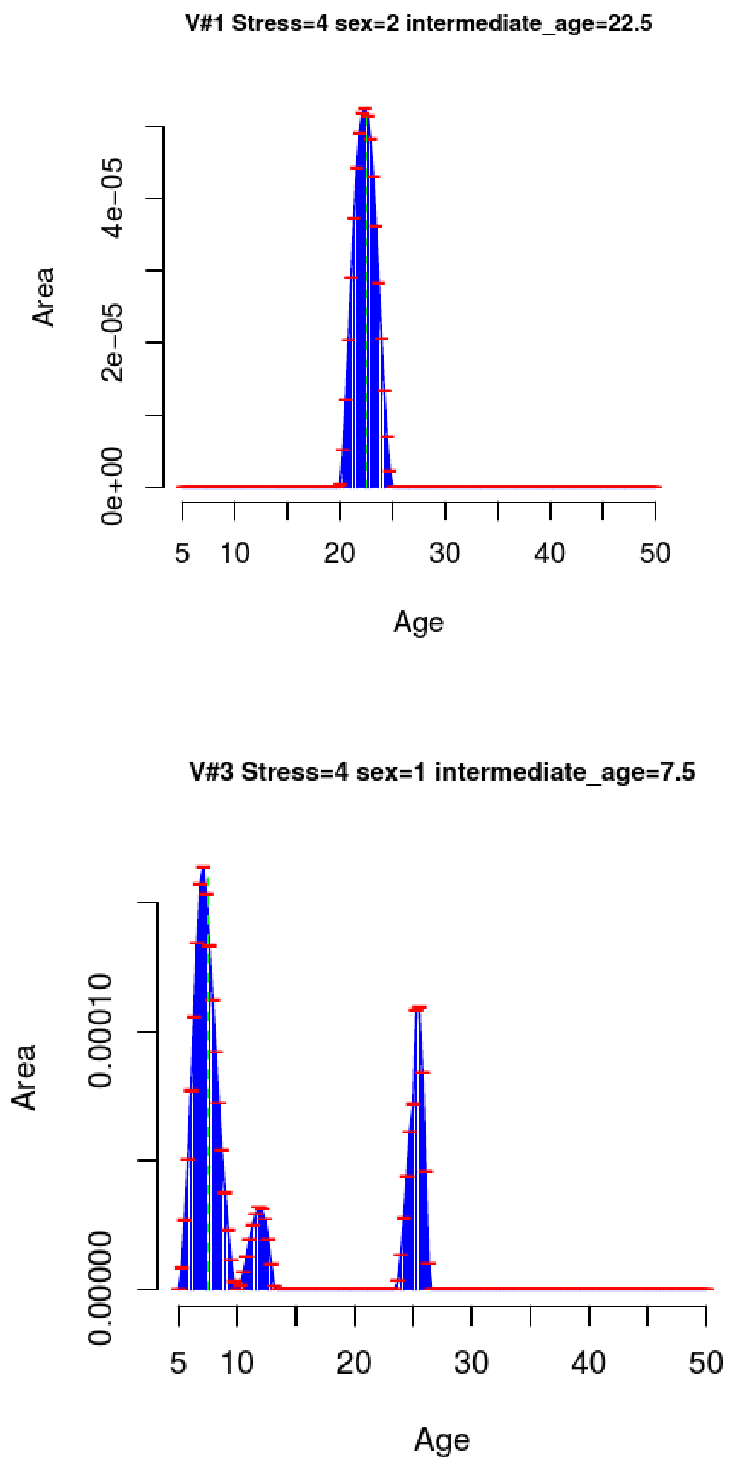

Fig. A7 Graphs for the intersections of the "sliding ellipse" intersections with the ellipse being considered, corresponding to the "intermediate" age.

4. For the obtained distribution of intersection area from age, we find the first two moments of the distribution (mathematical expectation and variance) using standard functions of the language R (sd and var, respectively). The expectation in this case is the sense of estimating the age for a given set of virtual data. Graphs for intersection areas of ellipses are presented in Online Resource 5.

5. The operations from paragraphs 2-4 of this algorithm repeat 100 sets of virtual data, the corresponding stress, sex and time point. Thus, we obtain 100 values for the estimation of age. Next,

calculate the mean and standard deviation for this set. The difference between the average and the building of the time point for which 100 sets of virtual data were generated shows an aspect of accuracy that reflects the nonlinearity of a given age interval. The standard deviation of these 100 values reflects the degree of linearity for estimates in a given age range, and the standard deviation is variations. Figure A8 shows the results of this step. For a full set of graphs, see Online Resource 5.

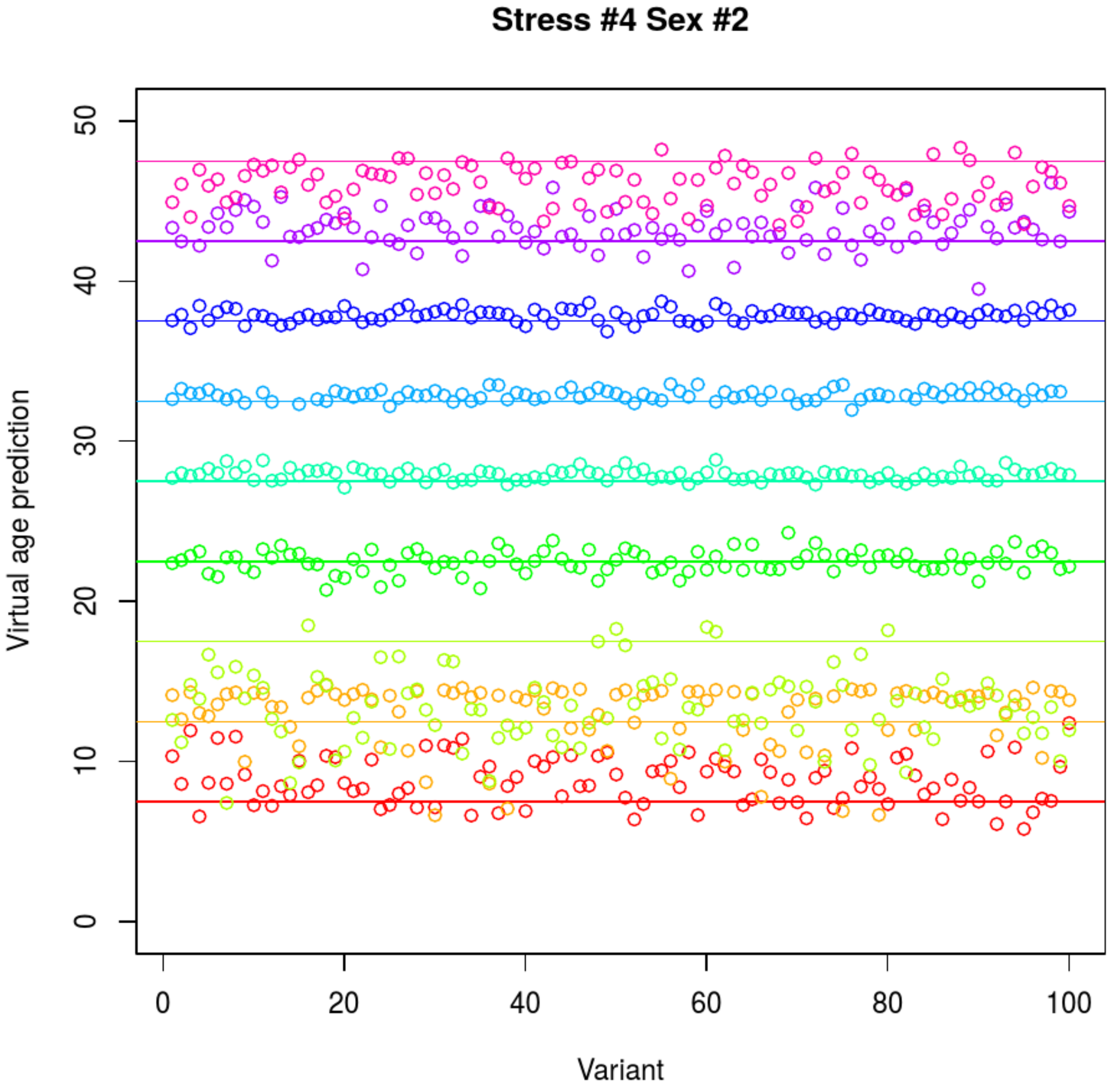

Fig. A8 For stress 4 (females), the results of estimates of the ages of previous data are presented. Colored circles marked the results of the assessment for each of the ~ 100 options, horizontal lines - expected estimates of age (values of intermediate ages 7.5, 12.5 ... 47.5 days)

6. Thus, for each age interval, gender, and stress, we obtain estimates for the accuracy, in which quality we take the variance of the estimates.

7. It should be noted that not completely considered variants are observed the intersection of the "sliding ellipse" and the ellipse of virtual data. In this case, the number of variants with intersections ("good" variants) is not equal to 100. (Fig. A9)

Number of good variants

	7.5	12.5	17.5	22.5	27.5	32.5	37.5	42.5	47.5
1–1	99	100	100	98	97	97	100	98	99
1–2	85	98	100	98	91	100	100	100	30
2–1	60	100	100	99	100	100	100	100	10
2–2	100	100	99	100	100	100	100	100	100
3–1	100	100	100	98	100	100	100	100	100
3–2	99	100	100	99	100	100	99	100	100
4–1	100	100	100	100	100	100	100	100	100
4–2	100	100	100	100	100	91	100	99	100
5–1	100	100	95	91	100	100	100	100	98
5–2	100	94	91	100	99	86	100	100	98
6–1	100	100	100	100	100	100	100	88	100
6–2	99	100	100	98	100	99	100	100	100
7–1	99	29	100	99	100	99	100	100	100
7–2	100	100	100	100	100	100	100	100	100
8–1	98	65	100	100	100	100	100	100	100
8–2	100	99	86	100	100	100	99	100	100
9–1	98	98	100	100	100	100	100	86	100
9–2	100	100	100	99	100	100	89	92	100
10–1	62	98	100	100	100	100	100	98	97
10–2	100	100	100	100	100	100	100	100	99
11–1	3	100	100	100	100	99	100	100	100
11–2	93	100	100	95	100	100	100	100	100
12–1	100	100	100	100	100	100	99	95	100
12–2	41	100	100	100	100	100	78	100	100
13–1	100	100	100	100	100	100	100	60	99
13–2	100	100	100	100	100	100	99	100	100

Fig. A9 The number of "good" options. Vertical labels (stress number-sex number), horizontal labels - intermediate ages.

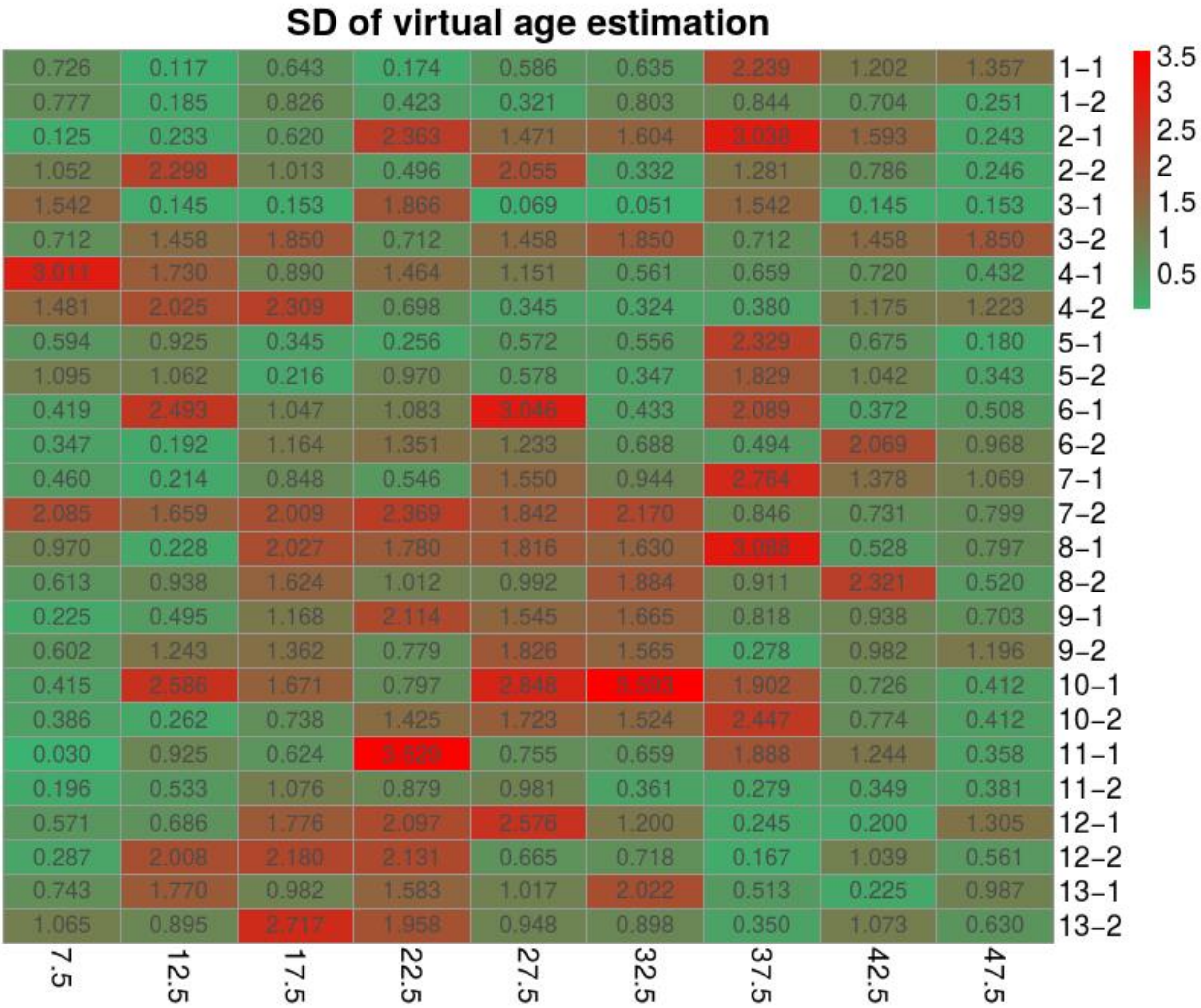

Fig. A10 Estimates for the standard deviation, the method accuracy characteristic for each cress, gender and age range, varies in days.

Deviation of virtual age estimation (days)

	7.5	12.5	17.5	22.5	27.5	32.5	37.5	42.5	47.5
1−1	0.021	1.458	0.242	0.574	0.410	0.008	1.785	0.554	0.426
1−2	0.026	0.689	0.436	0.170	0.372	0.701	0.662	0.914	1.909
2−1	1.044	0.894	5.964	2.321	3.240	0.522	1.423	0.273	1.856
2−2	0.149	1.627	0.971	0.835	10.282	0.546	1.421	0.046	0.377
3−1	11.005	1.842	3.169	2.293	12.721	17.619	11.005	1.842	3.169
3−2	0.714	10.676	5.475	0.714	10.676	5.475	0.714	10.676	5.475
4−1	5.567	0.364	0.976	3.381	0.596	0.101	0.251	0.346	0.169
4−2	1.253	0.472	4.116	0.026	0.413	0.392	0.356	0.710	1.476
5−1	0.777	0.156	0.284	0.255	0.290	0.529	3.531	0.274	0.060
5−2	1.477	0.999	0.569	0.127	0.030	0.193	1.634	0.389	0.085
6−1	0.166	5.121	2.370	2.181	0.856	0.927	1.232	0.112	0.764
6−2	0.008	0.196	1.088	0.445	0.170	0.200	0.250	0.579	0.315
7−1	0.401	0.465	0.280	0.312	2.067	1.102	4.551	1.875	0.075
7−2	3.388	0.378	0.745	0.445	0.188	0.207	3.247	1.969	5.283
8−1	0.770	0.684	1.575	0.206	2.014	0.509	3.152	0.849	0.286
8−2	1.651	2.315	1.201	1.078	3.545	0.384	0.385	1.568	0.667
9−1	0.405	0.226	0.257	2.355	0.847	1.770	0.096	0.611	2.619
9−2	0.256	2.042	2.519	0.365	1.039	0.608	0.210	0.252	0.393
10−1	0.248	2.211	9.968	4.063	2.996	5.207	0.478	0.541	0.623
10−2	0.067	0.144	0.250	10.195	2.957	1.883	2.348	0.297	0.404
11−1	7.518	0.085	2.084	4.069	0.419	0.628	2.112	0.166	0.308
11−2	0.131	1.119	0.076	0.507	0.079	0.042	0.036	0.556	0.314
12−1	0.184	0.097	3.583	0.114	1.980	0.168	0.069	0.186	0.806
12−2	0.385	1.201	1.514	2.423	1.443	3.772	0.001	0.144	0.025
13−1	0.203	11.538	6.478	0.295	3.773	7.816	0.507	0.184	2.977
13−2	0.691	0.037	0.867	5.516	1.732	2.774	0.280	0.001	0.006

Colour scale: 5, 10, 15

Fig. A11 The shift in the mean estimate relative to the expected for the mid-adult range, the linearity of the method for each cress, gender and age range varies in days.

Nelder JA, Mead R (1965) A simplex method for function minimization The computer journal 7:308-313

Nesterov P (2013) Visualization of a two-dimensional Gaussian on a plane. https://habrahabr.ru. https://habrahabr.ru/post/199060/. 2016
