# Supplementary material for: The Resistance of Drosophila melanogaster to Oxidative, Genotoxic, Proteotoxic, Osmotic Stress, Infection, and Starvation Depends on Age According to the Stress Factor
Source: Antioxidants (Basel). 2020 Dec 7;9(12):1239. doi: 10.3390/antiox9121239 (PMC7762242; doi:10.3390/antiox9121239)

The resistance of *Drosophila melanogaster* to oxidative, genotoxic, proteotoxic, osmotic stress, infection and starvation depends on age according to the stress factor

Alexei A. Belyi, Alexey A. Alekseev, Alexander Y. Fedintsev, Stepan N. Balybin, Ekaterina N. Proshkina, Mikhail V. Shaposhnikov and Alexey A. Moskalev

Online Resource 3

Figures in Online Resource 3 show the results of the original data bootstrapping on the plane (par1, par2). par1 and par2 - model parameters obtained by fitting the data for a certain age.

The dots indicate the results of the original data bootstrapping for different ages (Age = 5, 10, ... 50 days) when exposed to stress (stress factors transcript notation is provided in Online Resource. 4, Table A1). M - male, A - female.

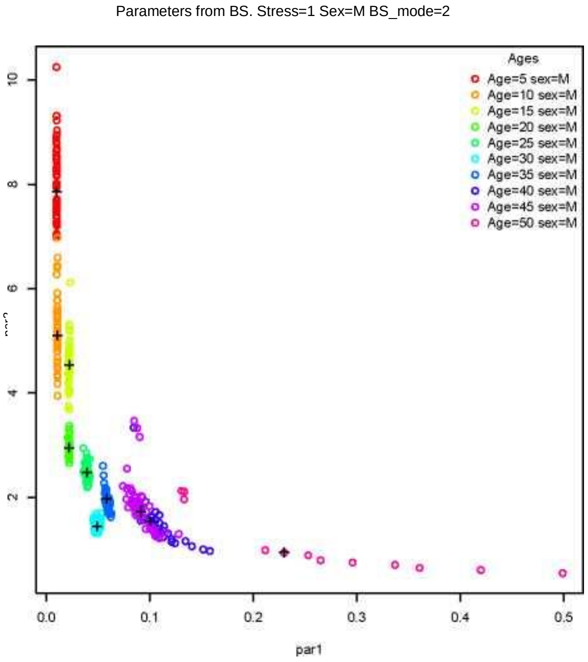
Parameters from BS. Stress=1 Sex=M BS_mode=2
Ages
Age=5 sex=M
Age=10 sex=M
Age=15 sex=M
Age=20 sex=M
Age=25 sex=M
Age=30 sex=M
Age=35 sex=M
Age=40 sex=M
Age=45 sex=M
Age=50 sex=M
10
8
6
4
2
0.0
0.1
0.2
0.3
0.4
0.5
par1

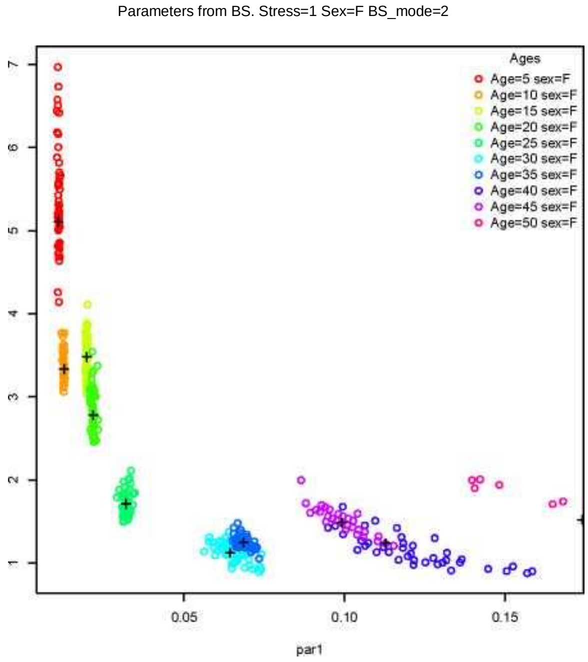
Parameters from BS. Stress=1 Sex=F BS_mode=2
Ages
Age=5 sex=F
Age=10 sex=F
Age=15 sex=F
Age=20 sex=F
Age=25 sex=F
Age=30 sex=F
Age=35 sex=F
Age=40 sex=F
Age=45 sex=F
Age=50 sex=F
7
6
5
4
3
2
1
0.05
0.10
0.15
par1

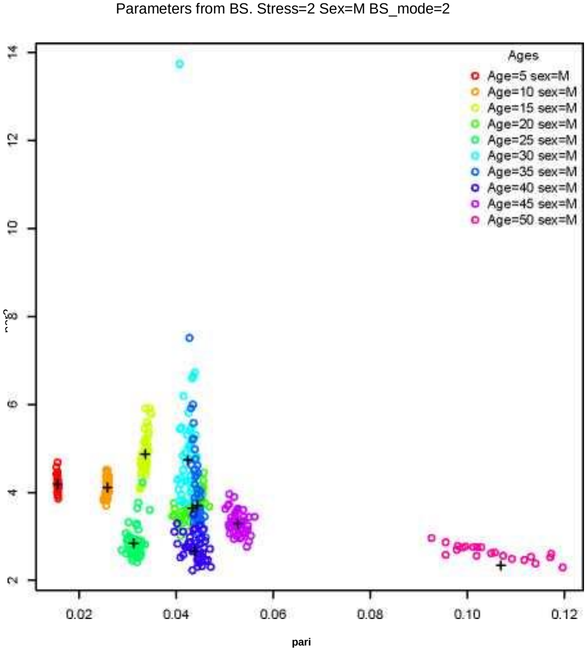
Parameters from BS. Stress=2 Sex=M BS_mode=2
Ages
Age=5 sex=M
Age=10 sex=M
Age=15 sex=M
Age=20 sex=M
Age=25 sex=M
Age=30 sex=M
Age=35 sex=M
Age=40 sex=M
Age=45 sex=M
Age=50 sex=M
14
12
10
8
6
4
2
0.02
0.04
0.06
0.08
0.10
0.12
pari

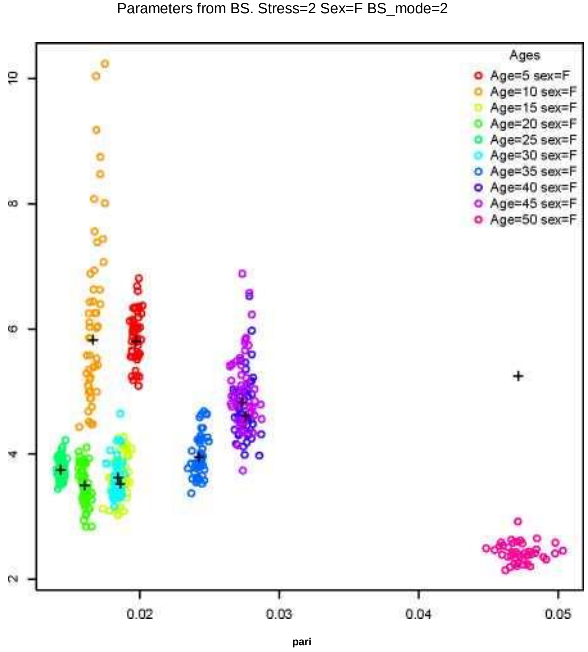
Parameters from BS. Stress=2 Sex=F BS_mode=2
Ages
Age=5 sex=F
Age=10 sex=F
Age=15 sex=F
Age=20 sex=F
Age=25 sex=F
Age=30 sex=F
Age=35 sex=F
Age=40 sex=F
Age=45 sex=F
Age=50 sex=F
10
8
6
4
2
0.02
0.03
0.04
0.05
pari

Parameters from BS. Stress=3 Sex=M BS_mode=2

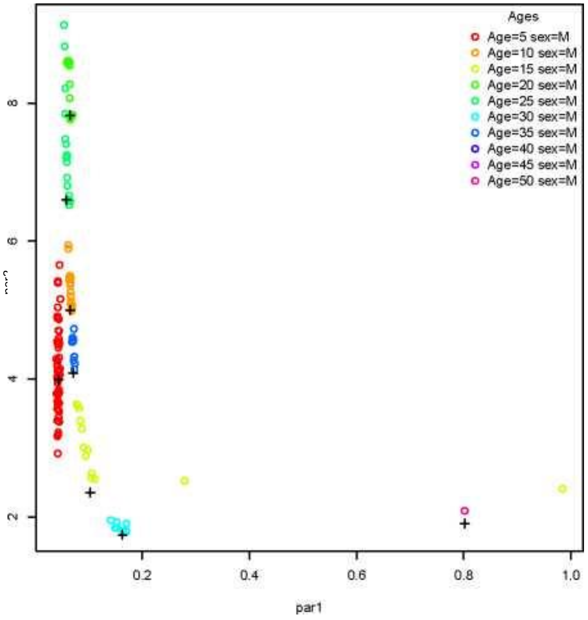

Parameters from BS. Stress=3 Sex=F BS_mode=2

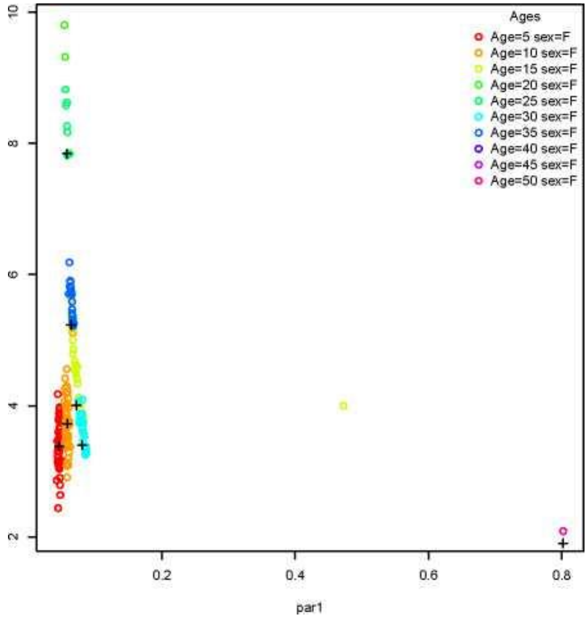

Parameters from BS. Stress=4 Sex=M BS_mode=2

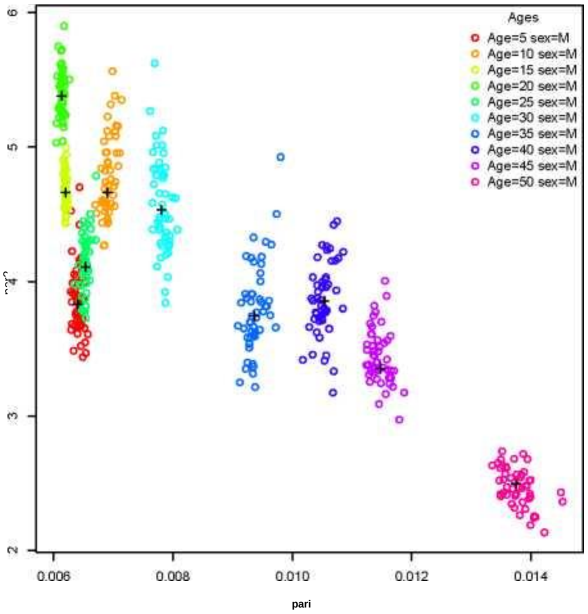

Parameters from BS. Stress=4 Sex=F BS_mode=2

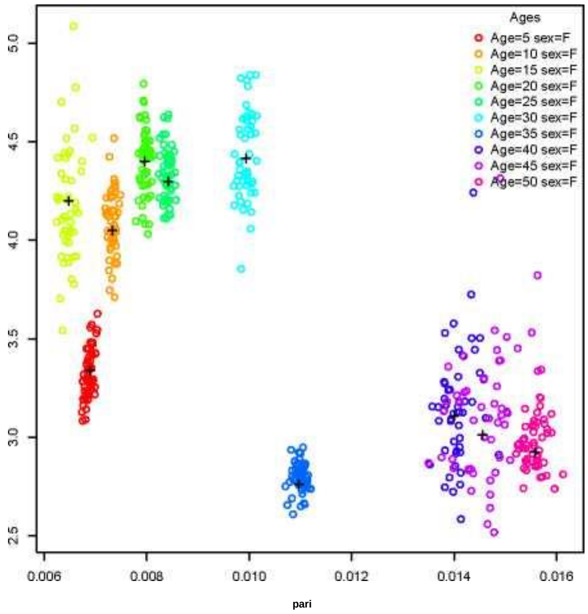

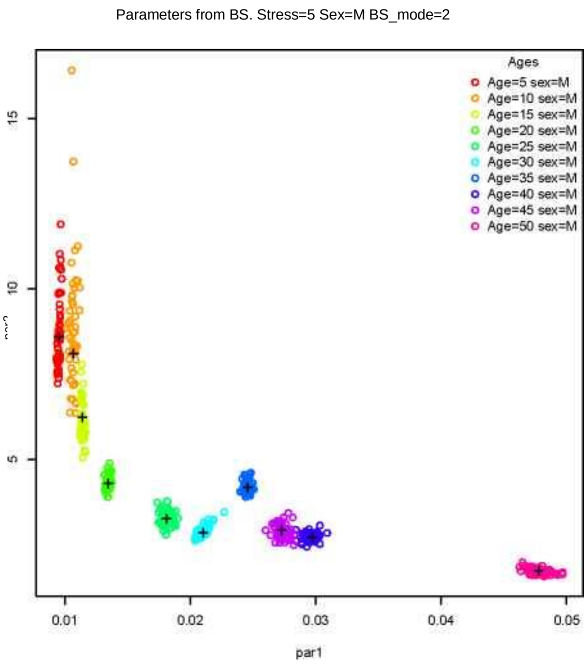
Parameters from BS. Stress=5 Sex=M BS_mode=2
Ages
Age=5 sex=M
Age=10 sex=M
Age=15 sex=M
Age=20 sex=M
Age=25 sex=M
Age=30 sex=M
Age=35 sex=M
Age=40 sex=M
Age=45 sex=M
Age=50 sex=M
5
10
15
0.01
0.02
0.03
0.04
0.05
par1

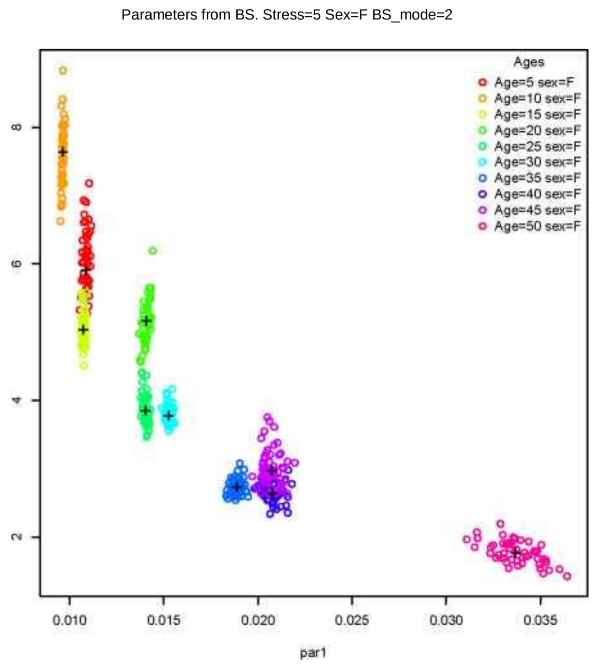
Parameters from BS. Stress=5 Sex=F BS_mode=2
Ages
Age=5 sex=F
Age=10 sex=F
Age=15 sex=F
Age=20 sex=F
Age=25 sex=F
Age=30 sex=F
Age=35 sex=F
Age=40 sex=F
Age=45 sex=F
Age=50 sex=F
2
4
6
8
0.010
0.015
0.020
0.025
0.030
0.035
par1

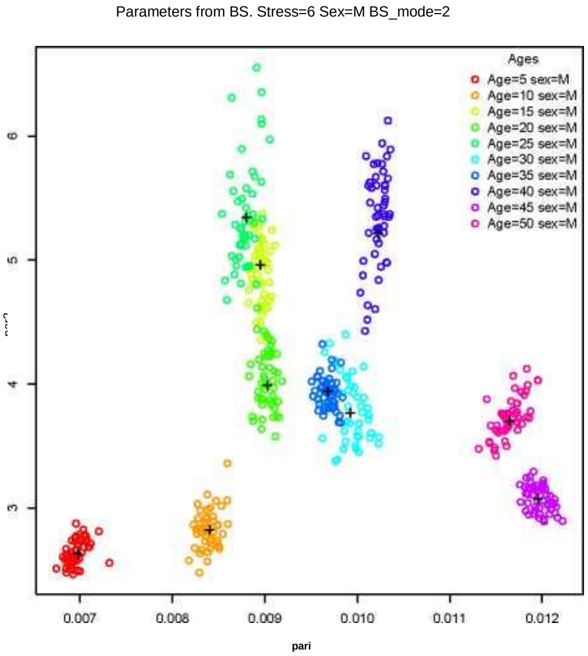
Parameters from BS. Stress=6 Sex=M BS_mode=2
Ages
Age=5 sex=M
Age=10 sex=M
Age=15 sex=M
Age=20 sex=M
Age=25 sex=M
Age=30 sex=M
Age=35 sex=M
Age=40 sex=M
Age=45 sex=M
Age=50 sex=M
3
4
5
6
0.007
0.008
0.009
0.010
0.011
0.012
pari

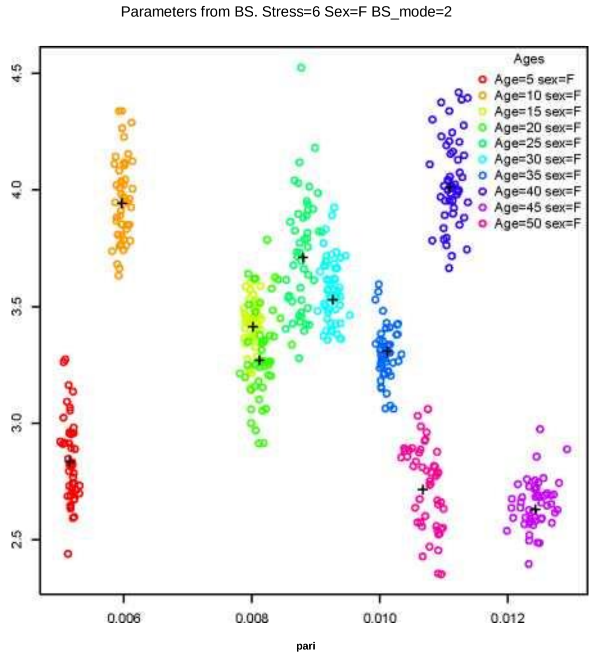
Parameters from BS. Stress=6 Sex=F BS_mode=2
Ages
Age=5 sex=F
Age=10 sex=F
Age=15 sex=F
Age=20 sex=F
Age=25 sex=F
Age=30 sex=F
Age=35 sex=F
Age=40 sex=F
Age=45 sex=F
Age=50 sex=F
2.5
3.0
3.5
4.0
4.5
0.006
0.008
0.010
0.012
pari

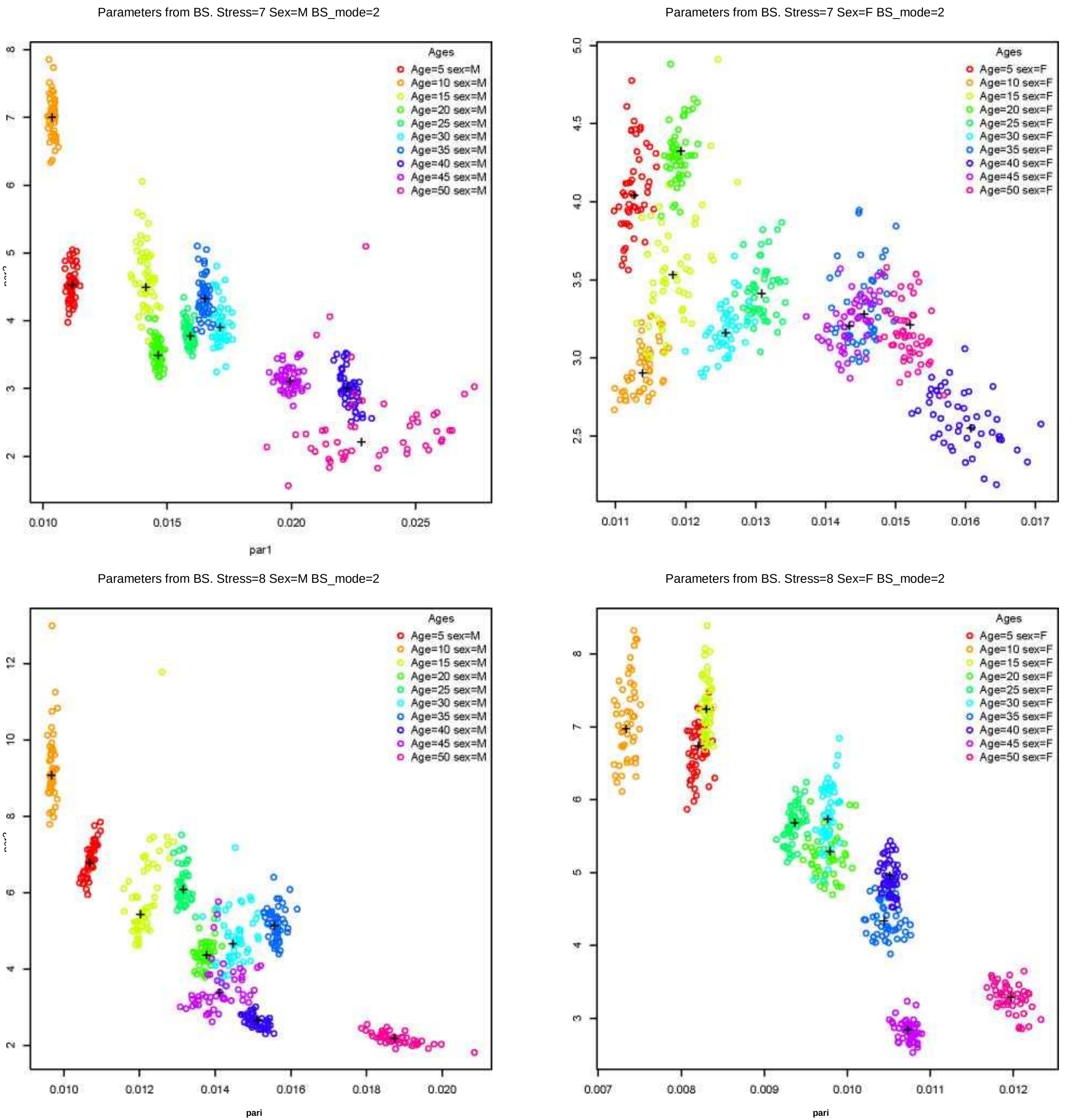
Parameters from BS. Stress=7 Sex=M BS_mode=2
Ages
Age=5 sex=M
Age=10 sex=M
Age=15 sex=M
Age=20 sex=M
Age=25 sex=M
Age=30 sex=M
Age=35 sex=M
Age=40 sex=M
Age=45 sex=M
Age=50 sex=M
par1
Parameters from BS. Stress=7 Sex=F BS_mode=2
Ages
Age=5 sex=F
Age=10 sex=F
Age=15 sex=F
Age=20 sex=F
Age=25 sex=F
Age=30 sex=F
Age=35 sex=F
Age=40 sex=F
Age=45 sex=F
Age=50 sex=F
Parameters from BS. Stress=8 Sex=M BS_mode=2
Ages
Age=5 sex=M
Age=10 sex=M
Age=15 sex=M
Age=20 sex=M
Age=25 sex=M
Age=30 sex=M
Age=35 sex=M
Age=40 sex=M
Age=45 sex=M
Age=50 sex=M
par1
Parameters from BS. Stress=8 Sex=F BS_mode=2
Ages
Age=5 sex=F
Age=10 sex=F
Age=15 sex=F
Age=20 sex=F
Age=25 sex=F
Age=30 sex=F
Age=35 sex=F
Age=40 sex=F
Age=45 sex=F
Age=50 sex=F
par1

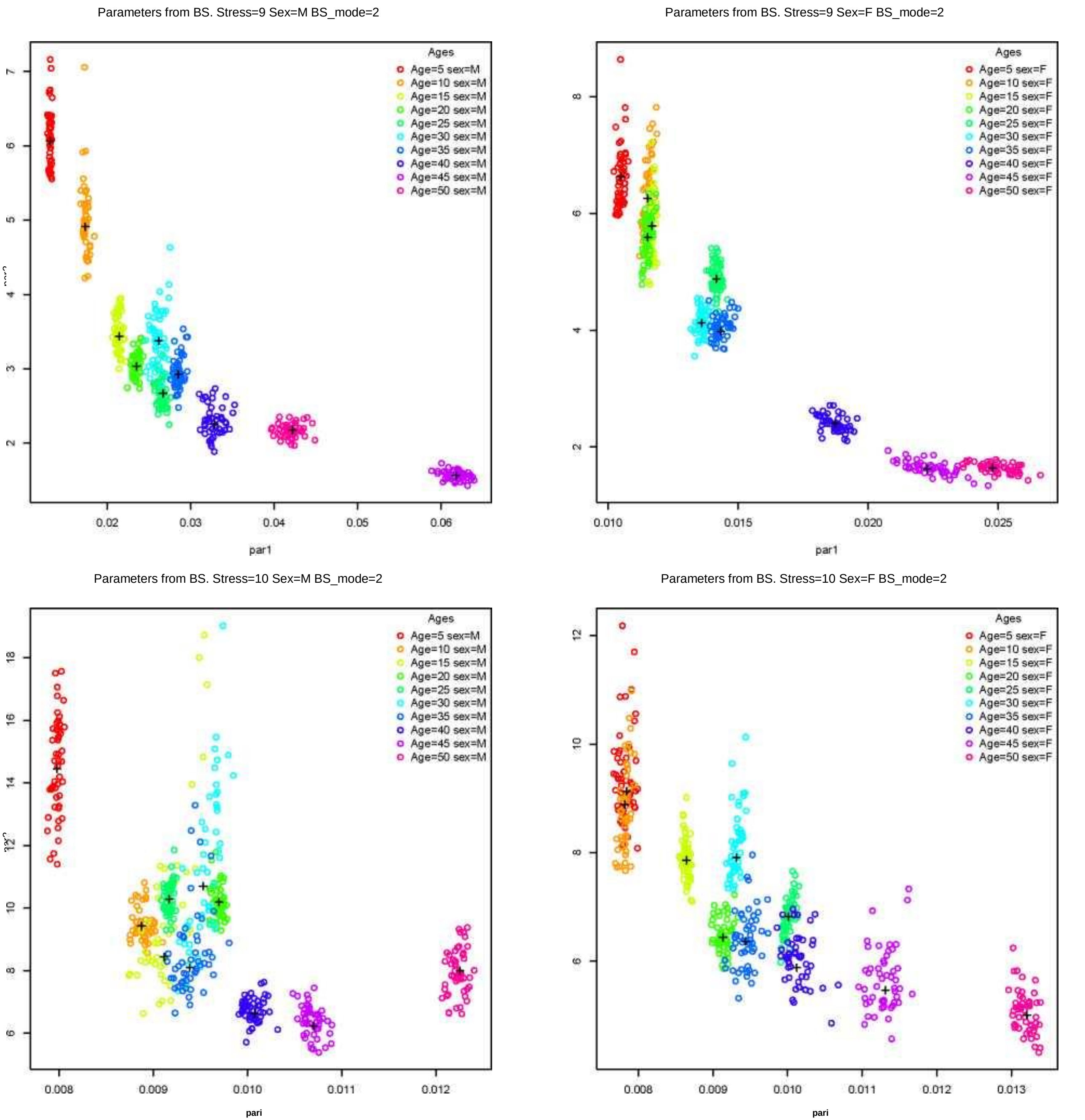
Parameters from BS. Stress=9 Sex=M BS_mode=2
Ages
Age=5 sex=M
Age=10 sex=M
Age=15 sex=M
Age=20 sex=M
Age=25 sex=M
Age=30 sex=M
Age=35 sex=M
Age=40 sex=M
Age=45 sex=M
Age=50 sex=M
par1
Parameters from BS. Stress=9 Sex=F BS_mode=2
Ages
Age=5 sex=F
Age=10 sex=F
Age=15 sex=F
Age=20 sex=F
Age=25 sex=F
Age=30 sex=F
Age=35 sex=F
Age=40 sex=F
Age=45 sex=F
Age=50 sex=F
par1
Parameters from BS. Stress=10 Sex=M BS_mode=2
Ages
Age=5 sex=M
Age=10 sex=M
Age=15 sex=M
Age=20 sex=M
Age=25 sex=M
Age=30 sex=M
Age=35 sex=M
Age=40 sex=M
Age=45 sex=M
Age=50 sex=M
Parameters from BS. Stress=10 Sex=F BS_mode=2
Ages
Age=5 sex=F
Age=10 sex=F
Age=15 sex=F
Age=20 sex=F
Age=25 sex=F
Age=30 sex=F
Age=35 sex=F
Age=40 sex=F
Age=45 sex=F
Age=50 sex=F

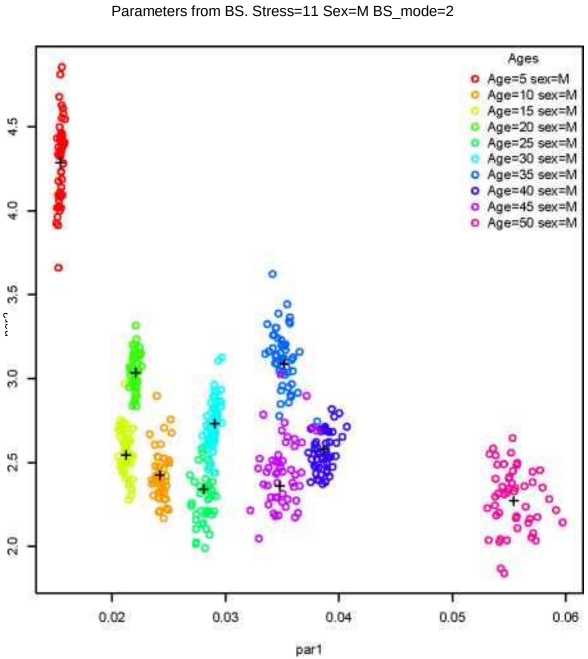
Parameters from BS. Stress=11 Sex=M BS_mode=2
Ages
Age=5 sex=M
Age=10 sex=M
Age=15 sex=M
Age=20 sex=M
Age=25 sex=M
Age=30 sex=M
Age=35 sex=M
Age=40 sex=M
Age=45 sex=M
Age=50 sex=M
par1

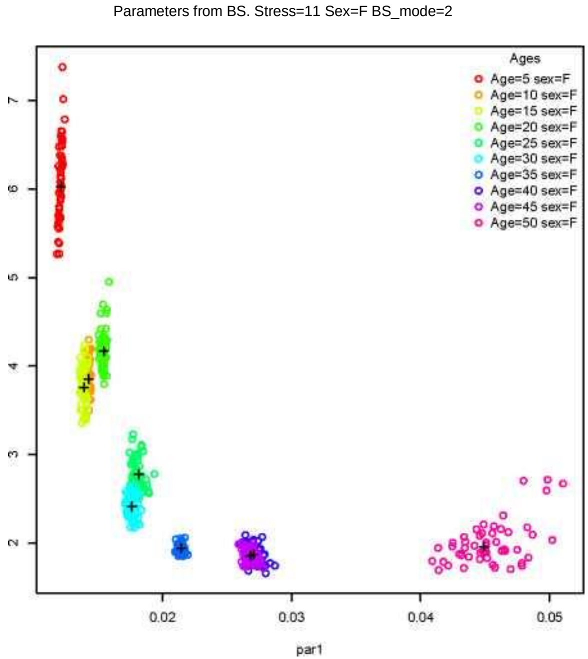
Parameters from BS. Stress=11 Sex=F BS_mode=2
Ages
Age=5 sex=F
Age=10 sex=F
Age=15 sex=F
Age=20 sex=F
Age=25 sex=F
Age=30 sex=F
Age=35 sex=F
Age=40 sex=F
Age=45 sex=F
Age=50 sex=F
par1

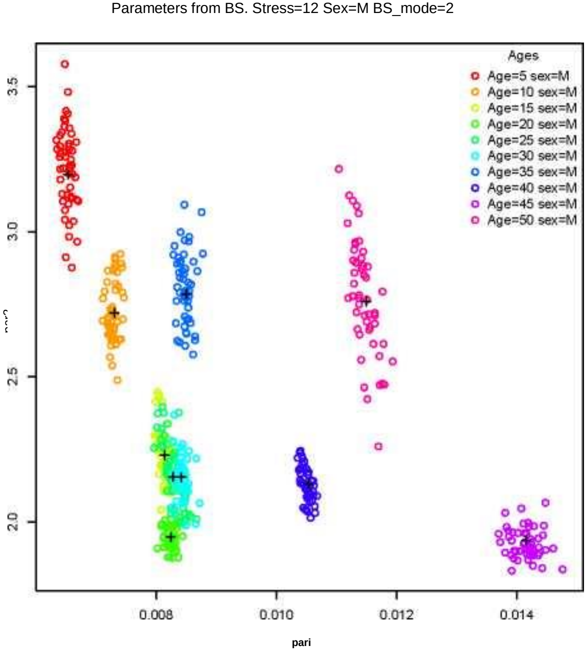
Parameters from BS. Stress=12 Sex=M BS_mode=2
Ages
Age=5 sex=M
Age=10 sex=M
Age=15 sex=M
Age=20 sex=M
Age=25 sex=M
Age=30 sex=M
Age=35 sex=M
Age=40 sex=M
Age=45 sex=M
Age=50 sex=M

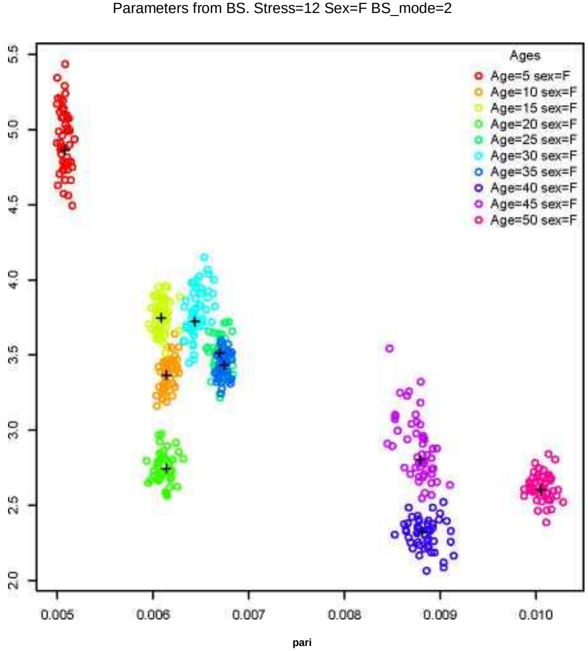
Parameters from BS. Stress=12 Sex=F BS_mode=2
Ages
Age=5 sex=F
Age=10 sex=F
Age=15 sex=F
Age=20 sex=F
Age=25 sex=F
Age=30 sex=F
Age=35 sex=F
Age=40 sex=F
Age=45 sex=F
Age=50 sex=F

Parameters from BS. Stress=13 Sex=M BS_mode=2

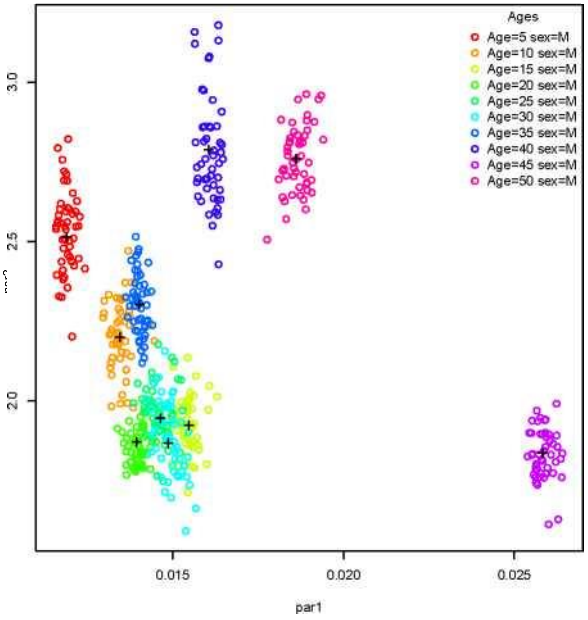

Parameters from BS. Stress=13 Sex=F BS_mode=2

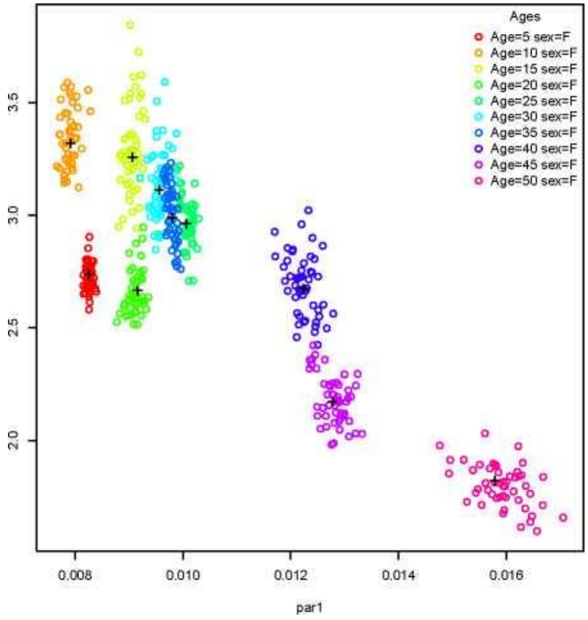

Supplement: Supplementary file 1 [file antioxidants-09-01239-s001.zip › Online Resource 3.pdf]
