# Supplementary material for: The Resistance of Drosophila melanogaster to Oxidative, Genotoxic, Proteotoxic, Osmotic Stress, Infection, and Starvation Depends on Age According to the Stress Factor
Source: Antioxidants (Basel). 2020 Dec 7;9(12):1239. doi: 10.3390/antiox9121239 (PMC7762242; doi:10.3390/antiox9121239)
Supplement: Supplementary file 1 [file antioxidants-09-01239-s001.zip › Online Resource 4.pdf]

The resistance of *Drosophila melanogaster* to oxidative, genotoxic, proteotoxic, osmotic stress, infection and starvation depends on age according to the stress factor

Alexei A. Belyi, Alexey A. Alekseev, Alexander Y. Fedintsev, Stepan N. Balybin, Ekaterina N. Proshkina, Mikhail V. Shaposhnikov and Alexey A. Moskalev

Online Resource 4

The data on *Drosophila melanogaster* stress resistance at different ages are presented below.

The effect of age on the survival rate of *Drosophila* after exposure to different stresses.

Variant of experiment		Sex					
Stressor	Age (days)	Male			Female		
		Mean ±SE (hours)	M (hours)	90% (hours)	Mean ±SE (hours)	M (hours)	90% (hours)
Paraquat	5	96.1 ±1.3	96	120	87 ±1.9	96	120
Paraquat	10	67.8 ±1.8	72	96	77.3 ±1.9	72	108
Paraquat	15	46.7 ±0.9	48	72	51.7 ±1.3	60	84
Paraquat	20	46.2 ±1.2	48	72	47 ±1.4	48	72
Paraquat	25	28.2 ±0.8	36	60	34.5 ±1.6	36	60
Paraquat	30	24.2 ±1	24	48	21.7 ±1.1	24	48
Paraquat	35	21.1 ±0.7	24	48	20.4 ±1	24	48
Paraquat	40	15.4 ±0.5	24	48	15.2 ±0.5	24	48
Paraquat	45	15.9 ±0.5	24	48	15.6 ±0.5	24	48
Paraquat	50	13 ±0.3	24	48	12.6 ±0.2	24	48
Starvation	5	64 ±1.3	60	84	52.7 ±0.8	60	84
Starvation	10	40.6 ±0.8	48	72	62.6 ±1	72	96
Starvation	15	32.7 ±0.7	36	60	55 ±1.3	60	84
Starvation	20	26.9 ±0.6	36	60	60.9 ±1.5	60	84
Starvation	25	34.5 ±0.9	36	60	68.2 ±1.7	72	96
Starvation	30	27.6 ±0.5	36	60	54.3 ±1.3	60	84
Starvation	35	26.2 ±0.6	36	60	43 ±1	48	72
Starvation	40	25.8 ±0.7	36	60	38.3 ±0.9	48	72
Starvation	45	23 ±0.5	36	60	38.9 ±0.8	48	72
Starvation	50	14 ±0.4	24	48	24.5 ±0.7	36	60
Hyperthermia	5	26.7 ±0.6	36	48	25.4 ±0.6	36	48
Hyperthermia	10	20.9 ±0.4	24	48	21.7 ±0.5	36	48
Hyperthermia	15	14.3 ±0.4	24	48	18.9 ±0.5	24	48
Hyperthermia	20	22.4 ±0.3	36	48	23.4 ±0.2	36	48
Hyperthermia	25	22.9 ±0.3	36	48	23.4 ±0.2	36	48
Hyperthermia	30	12.5 ±0.2	24	48	17 ±0.5	24	48
Hyperthermia	35	19.1 ±0.5	24	48	21.4 ±0.4	36	48
Hyperthermia	40	12 ±0	24	48	12 ±0	24	48
Hyperthermia	45	12 ±0	24	48	12 ±0	24	48
Hyperthermia	50	12 ±0	24	48	12 ±0	24	48
5 mM $ZnCl_2$	5	140.6 ±5.3	120	204	184.5 ±6.5	180	252
5 mM $ZnCl_2$	10	117.6 ±3.8	108	204	162.5 ±3.9	156	228

5 mM $ZnCl_2$	15	108.4 ±2.1	108	144	120.6 ±2.9	120	168
5 mM $ZnCl_2$	20	107 ±2.6	108	144	120 ±3.3	108	180
5 mM $ZnCl_2$	25	110.1 ±2	108	144	109.8 ±2.4	108	156
5 mM $ZnCl_2$	30	94.8 ±2.6	96	132	103.7 ±2.6	108	144
5 mM $ZnCl_2$	35	99.3 ±2.3	96	132	96.1 ±2.6	96	132
5 mM $ZnCl_2$	40	95.6 ±1.9	96	120	88.8 ±2	84	120
5 mM $ZnCl_2$	45	79 ±2.3	84	108	78.1 ±2.7	72	120
5 mM $ZnCl_2$	50	83.7 ±2.1	84	108	90.7 ±3	84	132
10 mM $ZnCl_2$	5	87.7 ±1.7	84	120	87 ±1.8	84	120
10 mM $ZnCl_2$	10	96.3 ±1.3	96	120	85.8 ±2.6	84	132
10 mM $ZnCl_2$	15	71 ±1.4	72	96	87.3 ±2.3	84	120
10 mM $ZnCl_2$	20	66.6 ±1.7	72	96	82.8 ±1.7	84	108
10 mM $ZnCl_2$	25	61.9 ±1.5	60	84	74.5 ±1.9	72	108
10 mM $ZnCl_2$	30	58.7 ±1.3	60	84	77.1 ±2	72	108
10 mM $ZnCl_2$	35	60.7 ±1.3	60	84	67.8 ±1.7	72	96
10 mM $ZnCl_2$	40	44.7 ±1.3	48	72	62.8 ±2	60	96
10 mM $ZnCl_2$	45	50.6 ±1.4	60	84	66.9 ±1.9	72	96
10 mM $ZnCl_2$	50	39 ±1.3	48	72	64 ±1.9	72	96
10 mM $FeCl_3$	5	141.1 ±3.8	144	204	185.6 ±3.5	192	240
10 mM $FeCl_3$	10	126.5 ±3.9	132	204	155.2 ±3.8	144	228
10 mM $FeCl_3$	15	112.2 ±4.1	120	180	155 ±3.4	156	204
10 mM $FeCl_3$	20	111 ±4.5	120	180	152.3 ±4.4	132	240
10 mM $FeCl_3$	25	108.6 ±4.1	120	168	140.5 ±3.4	144	192
10 mM $FeCl_3$	30	107.9 ±4.3	108	180	147.4 ±3.4	144	204
10 mM $FeCl_3$	35	107.4 ±3.5	108	156	140.1 ±3.4	132	204
10 mM $FeCl_3$	40	86.5 ±3.3	96	132	106.6 ±3.7	108	168
10 mM $FeCl_3$	45	67.5 ±2.6	72	108	105.2 ±3.4	108	156
10 mM $FeCl_3$	50	81 ±2.6	84	120	93.6 ±3.1	96	144
15 mM $FeCl_3$	5	81.6 ±2.5	72	132	114.5 ±3.4	108	180
15 mM $FeCl_3$	10	72.2 ±2.4	60	120	120.6 ±2.9	108	168
15 mM $FeCl_3$	15	62.6 ±2.4	60	96	106.8 ±2.8	96	156
15 mM $FeCl_3$	20	68.5 ±2.7	60	108	104.4 ±3.3	96	156
15 mM $FeCl_3$	25	65.4 ±2.5	60	108	94.9 ±2.6	96	144
15 mM $FeCl_3$	30	63.8 ±2.6	60	108	100.7 ±2.7	96	144
15 mM $FeCl_3$	35	67.7 ±2.4	72	108	96.7 ±2.7	96	132
15 mM $FeCl_3$	40	60.3 ±1.9	60	84	77.7 ±2.4	84	108
15 mM $FeCl_3$	45	40 ±1.7	36	60	73 ±2.8	72	120
15 mM $FeCl_3$	50	53.5 ±1.5	60	84	61.2 ±2.5	60	108
10 mM $CuSO_4$	5	93.9 ±1.3	96	120	121.3 ±1.9	120	144
10 mM $CuSO_4$	10	104.2 ±1.3	108	132	136.2 ±2	132	168
10 mM $CuSO_4$	15	83.3 ±1.5	84	108	118.7 ±1.5	120	144
10 mM $CuSO_4$	20	71.4 ±1.6	72	96	100.8 ±1.7	96	132
10 mM $CuSO_4$	25	76.7 ±1.2	84	108	104.7 ±1.7	108	132
10 mM $CuSO_4$	30	69 ±1.4	72	96	100.8 ±2.1	108	132
10 mM $CuSO_4$	35	65.1 ±1.3	72	96	91.6 ±1.9	96	120
10 mM $CuSO_4$	40	60.6 ±2	60	96	92.6 ±1.9	96	120
10 mM $CuSO_4$	45	65.4 ±1.4	60	96	86.3 ±2.8	96	120
10 mM $CuSO_4$	50	51.4 ±1.9	60	84	78.9 ±2.2	84	108
15 mM $CuSO_4$	5	76.7 ±1.2	84	108	95.8 ±1.4	96	120
15 mM $CuSO_4$	10	59.4 ±1	60	84	88.4 ±1.4	96	120
15 mM $CuSO_4$	15	47.8 ±1.2	48	72	85.7 ±1.5	84	108
15 mM $CuSO_4$	20	44.2 ±1.2	48	72	87.6 ±1.8	84	108
15 mM $CuSO_4$	25	39.4 ±1.1	36	60	71 ±1.3	72	96
15 mM $CuSO_4$	30	40.4 ±1	48	72	72.6 ±1.6	72	96
15 mM $CuSO_4$	35	37.4 ±1	36	60	68.9 ±1.5	72	96

15 mM $CuSO_4$	40	32.6±1.1	36	60	52.7±1.7	48	84
15 mM $CuSO_4$	45	20.6±0.8	24	48	45.4±1.9	36	84
15 mM $CuSO_4$	50	26.7±0.9	36	60	41.4±1.7	36	72
400 mM NaCl	5	105.4±1.3	108	132	91.7±1.4	96	120
400 mM NaCl	10	93.8±1.2	96	120	104.6±1.4	108	132
400 mM NaCl	15	86±1.5	96	120	92.2±1.9	96	120
400 mM NaCl	20	73.1±1.6	72	96	71.8±1.4	72	96
400 mM NaCl	25	55.8±1.4	60	84	70.3±1.6	72	96
400 mM NaCl	30	49.1±1.4	48	72	64.8±1.5	60	96
400 mM NaCl	35	43.6±1	48	72	53.7±1.5	48	84
400 mM NaCl	40	35.7±1	36	60	49.4±1.5	48	72
400 mM NaCl	45	38.6±1.1	48	72	#ЗНАЧ!	48	72
400 mM NaCl	50	24.3±0.9	24	48	31.8±1.2	36	60
1 mM $CdCl_2$	5	128.8±1.1	132	156	127±1.3	132	156
1 mM $CdCl_2$	10	113.5±1.3	120	144	129.8±1.7	132	156
1 mM $CdCl_2$	15	110.1±1.3	108	132	115.6±1.4	120	144
1 mM $CdCl_2$	20	104.7±1	108	132	108.1±1.5	108	132
1 mM $CdCl_2$	25	110.4±1.1	120	144	99.5±1.4	96	120
1 mM $CdCl_2$	30	108.2±1.4	108	132	107.7±1.6	108	132
1 mM $CdCl_2$	35	107.3±1.6	108	132	106.2±1.7	108	132
1 mM $CdCl_2$	40	97.7±1.5	96	120	96.9±1.6	96	120
1 mM $CdCl_2$	45	92.1±1.5	96	120	86.9±1.6	84	108
1 mM $CdCl_2$	50	80.5±1.6	96	120	73.8±1.6	84	108
5 mM $CdCl_2$	5	65.4±1.3	72	96	83±1.4	84	108
5 mM $CdCl_2$	10	43.6±1.3	48	72	69.4±1.6	72	96
5 mM $CdCl_2$	15	48.6±1.5	48	72	70.3±1.7	72	96
5 mM $CdCl_2$	20	47.8±1.3	48	72	64.9±1.4	72	96
5 mM $CdCl_2$	25	38.6±1.4	36	60	55.3±1.6	48	84
5 mM $CdCl_2$	30	38.2±1.3	36	60	55.8±1.8	60	84
5 mM $CdCl_2$	35	31.9±0.8	36	60	46.7±1.7	48	72
5 mM $CdCl_2$	40	29.5±0.9	36	60	39.4±1.6	36	60
5 mM $CdCl_2$	45	31.6±1	36	60	39.6±1.6	36	60
5 mM $CdCl_2$	50	22±0.7	24	48	26.6±1.1	36	60
Infection	5	147.6±3.3	144	204	138.6±3.4	132	204
Infection	10	139.6±2.9	144	180	131.7±2.9	132	180
Infection	15	153.3±3.1	156	192	147.3±3	144	192
Infection	20	158.2±2.7	156	216	122.4±2.4	120	156
Infection	25	146.2±3.2	144	204	115.6±2.5	108	144
Infection	30	122±2.6	120	156	98.2±2	96	132
Infection	35	103.5±2.7	108	144	88.6±2.6	84	132
Infection	40	92.2±2.3	96	132	68.4±1.9	72	96
Infection	45	83.6±2.3	84	120	68.5±1.9	60	96
Infection	50	69±2.2	72	108	63.9±1.8	60	96

Table A1 The effect of age on the survival rate of *Drosophila* after exposure to different stresses. Mean±SE- mean survival, M - median survival, 90% - time of death of 90% of the specimen in the sample

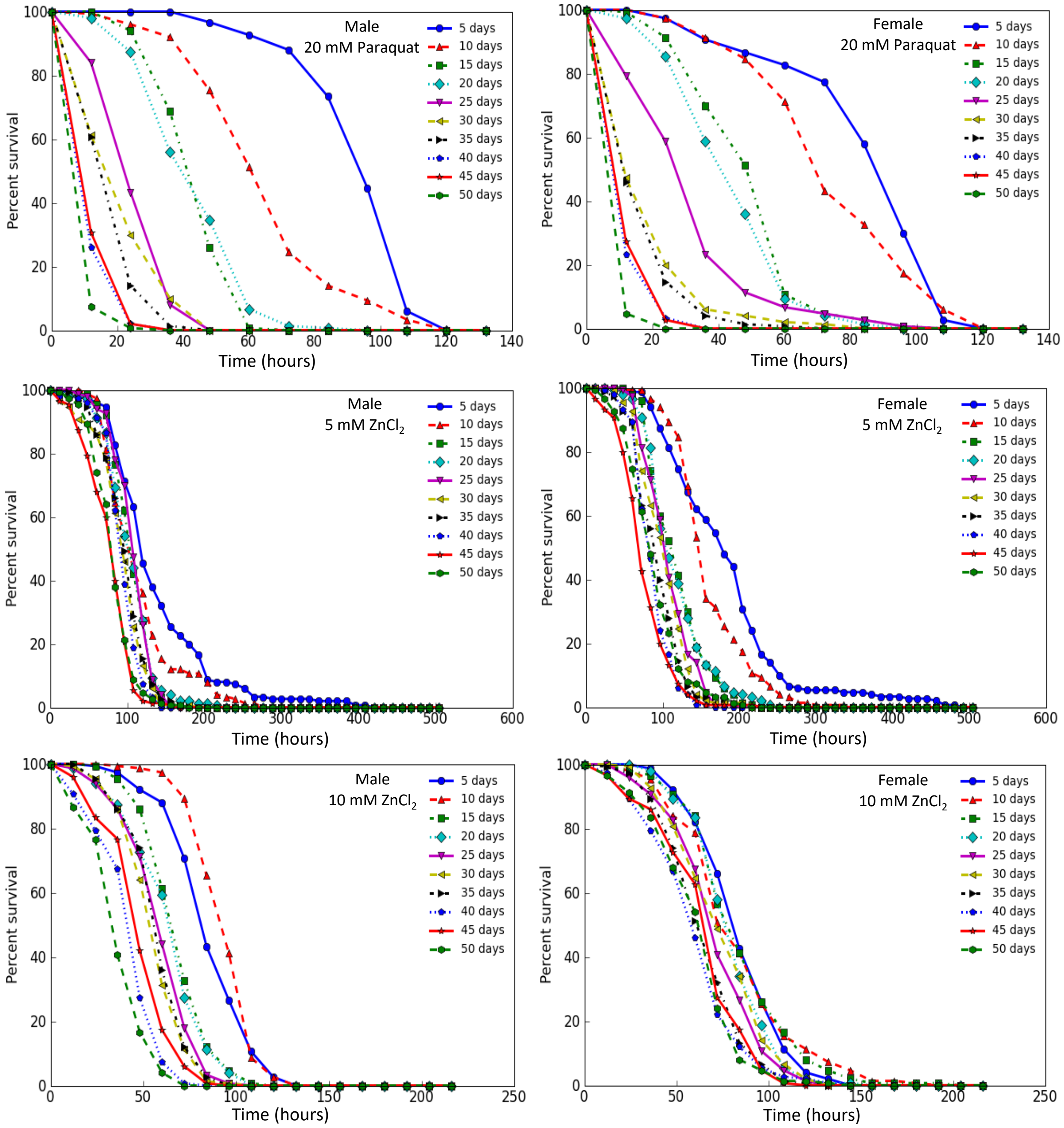

Fig. A1 Survival curves of *D. melanogaster* at different ages under exposure of oxidative stress. The graphs show the change in survival with increasing age of flies (5, 10, ..., 50 days) under expose of the stresses

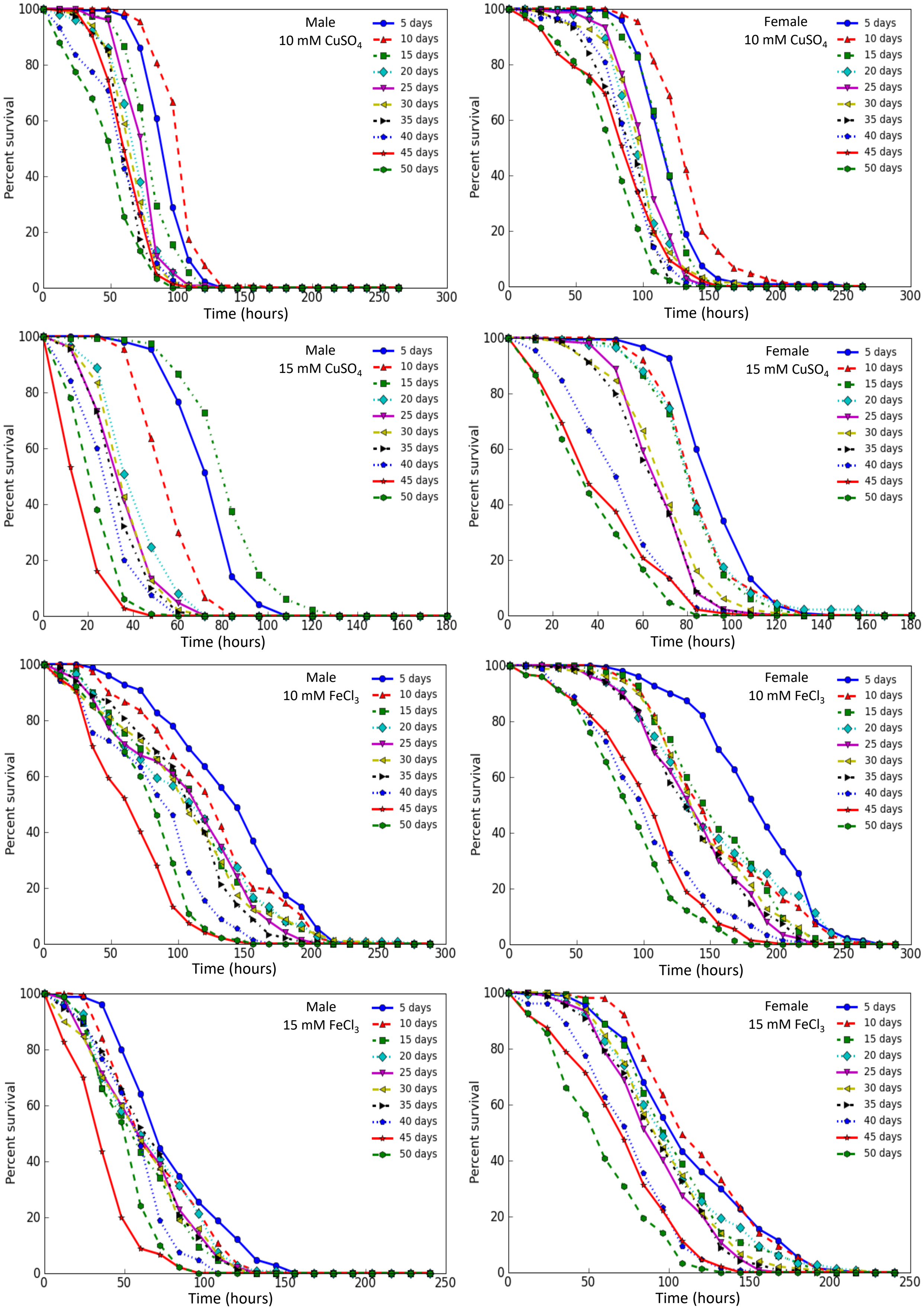

Fig. A2 Survival curves of *D. melanogaster* at different ages under exposure of oxidative stress. The graphs show the change in survival with increasing age of flies (5, 10, ..., 50 days) under expose of the stresses

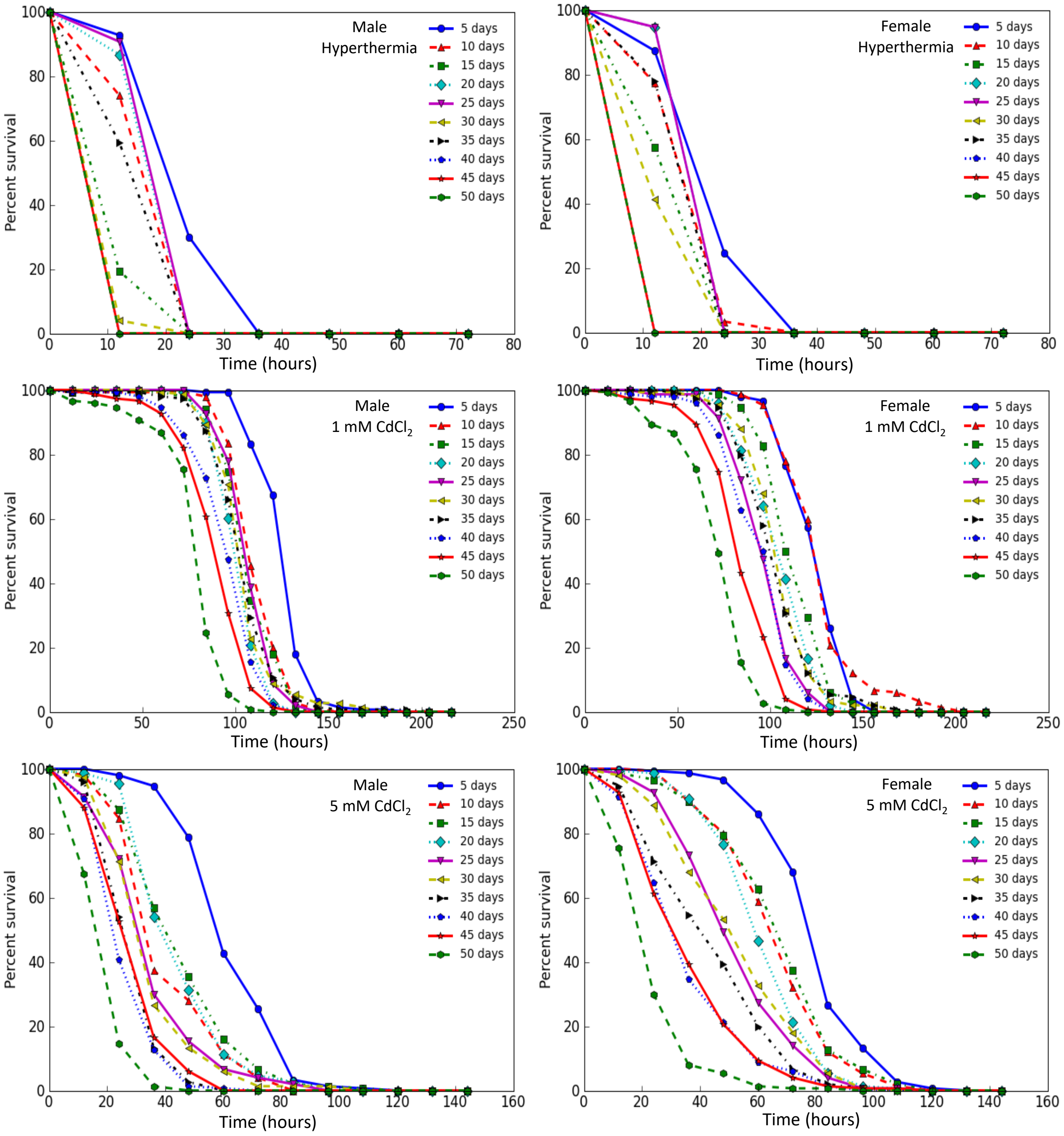

Fig. A3 Survival curves of *D. melanogaster* at different ages under exposure of oxidative stress. The graphs show the change in survival with increasing age of flies (5, 10, ..., 50 days) under expose of the stresses

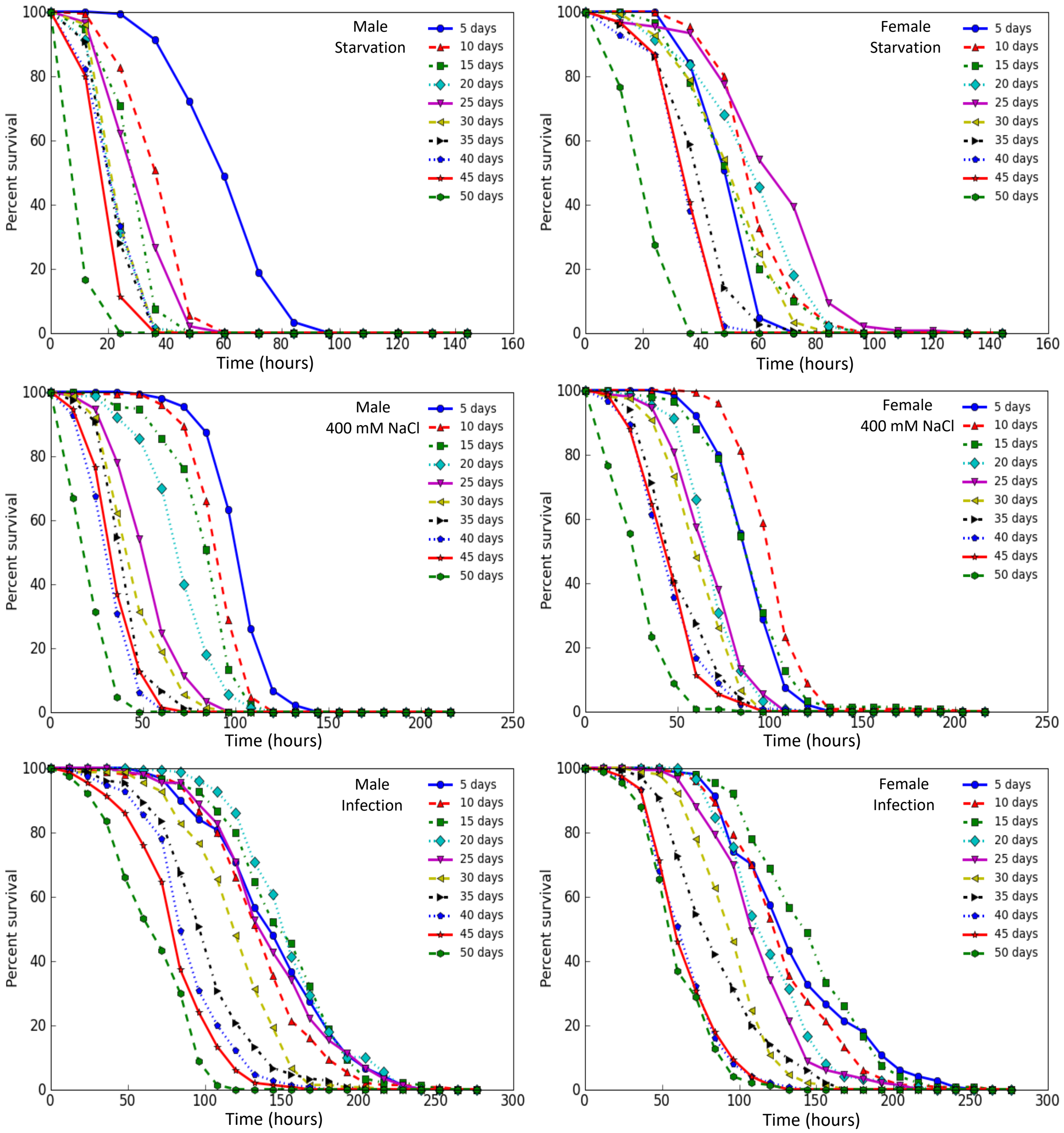

Fig. A4 Survival curves of *D. melanogaster* at different ages under exposure of oxidative stress. The graphs show the change in survival with increasing age of flies (5, 10, ..., 50 days) under expose of the following stresses: starvation (a – male, b - female), 400 mM NaCl (c – male, d - female), infection by *B. bassiana* (e – male, f - female)
